# Supplementary material for: Immediate early splicing controls translation in activated T-cells and is mediated by hnRNPC2 phosphorylation
Source: EMBO J. 2025 Feb 13;44(6):1692–723. doi: 10.1038/s44318-025-00374-8 (PMC11914300; doi:10.1038/s44318-025-00374-8)
Supplement: Supplementary file 1 — Appendix [file 44318_2025_374_MOESM1_ESM.pdf]

Appendix for

Immediate early splicing controls translation in activated T-cells and is mediated by hnRNPC2 phosphorylation

Mateusz Dróżdż[1], Luíza Zuvanov[1], Gopika Sasikumar[2], Debojit Bose[1], Franziska Bruening[3], Maria S. Robles[3], Marco Preußner[1], Markus Wahl[2], Florian Heyd[1*]

[1] Institute of Chemistry and Biochemistry, Laboratory of RNA Biochemistry, Freie Universität Berlin, Takustr. 6, 14195 Berlin, Germany

[2] Institute of Chemistry and Biochemistry, Laboratory of Structural Biochemistry, Freie Universität Berlin, Takustr. 6, 14195 Berlin, Germany

[3] Institute of Medical Psychology and Biomedical Center, Faculty of Medicine, LMU, Munich, Germany

[*]Corresponding author: Takustr. 6, 14195 Berlin, Germany, ++49-30-83862938,

florian.heyd@fu-berlin.de

Table of contests:

Appendix Fig. S1: 1-2

Appendix Fig. S2: 3-4

Appendix Fig. S3: 5-6

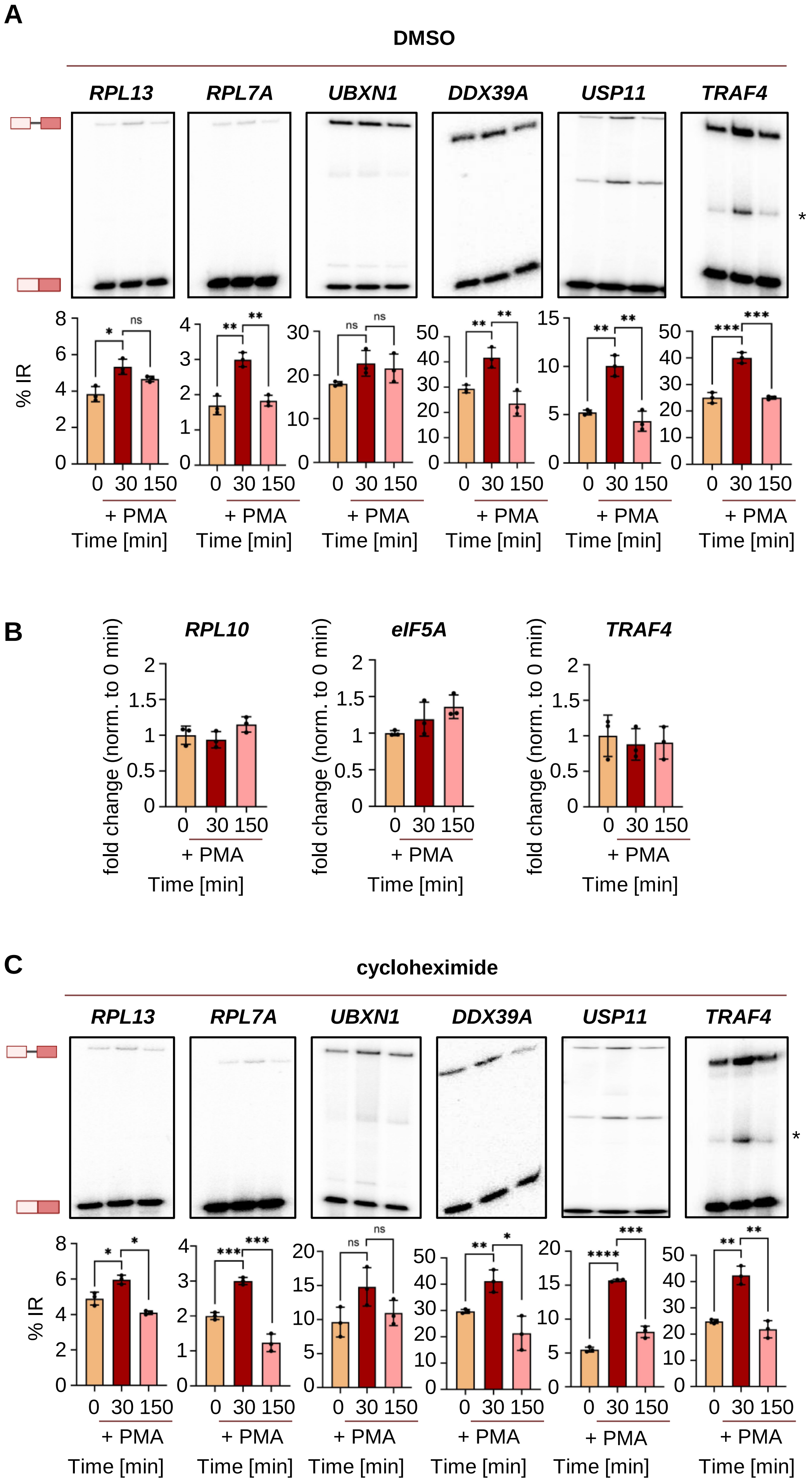
A
DMSO
RPL13
RPL7A
UBXN1
DDX39A
USP11
TRAF4
*
% IR
0 30 150
+ PMA
Time [min]
B
RPL10
eIF5A
TRAF4
fold change (norm. to 0 min)
0 30 150
+ PMA
Time [min]
C
cycloheximide
RPL13
RPL7A
UBXN1
DDX39A
USP11
TRAF4
*
% IR
0 30 150
+ PMA
Time [min]

Appendix Fig. S1. IES validation in T cells upon PMA stimulation

A. Jurkat cells were stimulated with PMA for the indicated times. Cells were harvested and chromatin-associated RNA was extracted and selected IES events were analyzed by radioactive, splicing-sensitive RT-PCR (top; * - degradation product) and quantified (bottom, % IR).

B. Constant expression of target genes upon PMA stimulation. Jurkat T cells were stimulated with PMA at the indicated time points, after which total RNA was extracted. Target gene expression was determined by RT-qPCR. mRNA expression levels are quantified relative to hHPRT (fold change normalized to 0 min).

C. Jurkat cells were stimulated and analyzed as in A but cycloheximide was present during PMA stimulation.

Data information: data was presented as mean ± SD, n = 3, ns: non-significant, *p<0.05, **p<0.01, ***p<0.001, ****p<0.0001 (unpaired student's t-test).

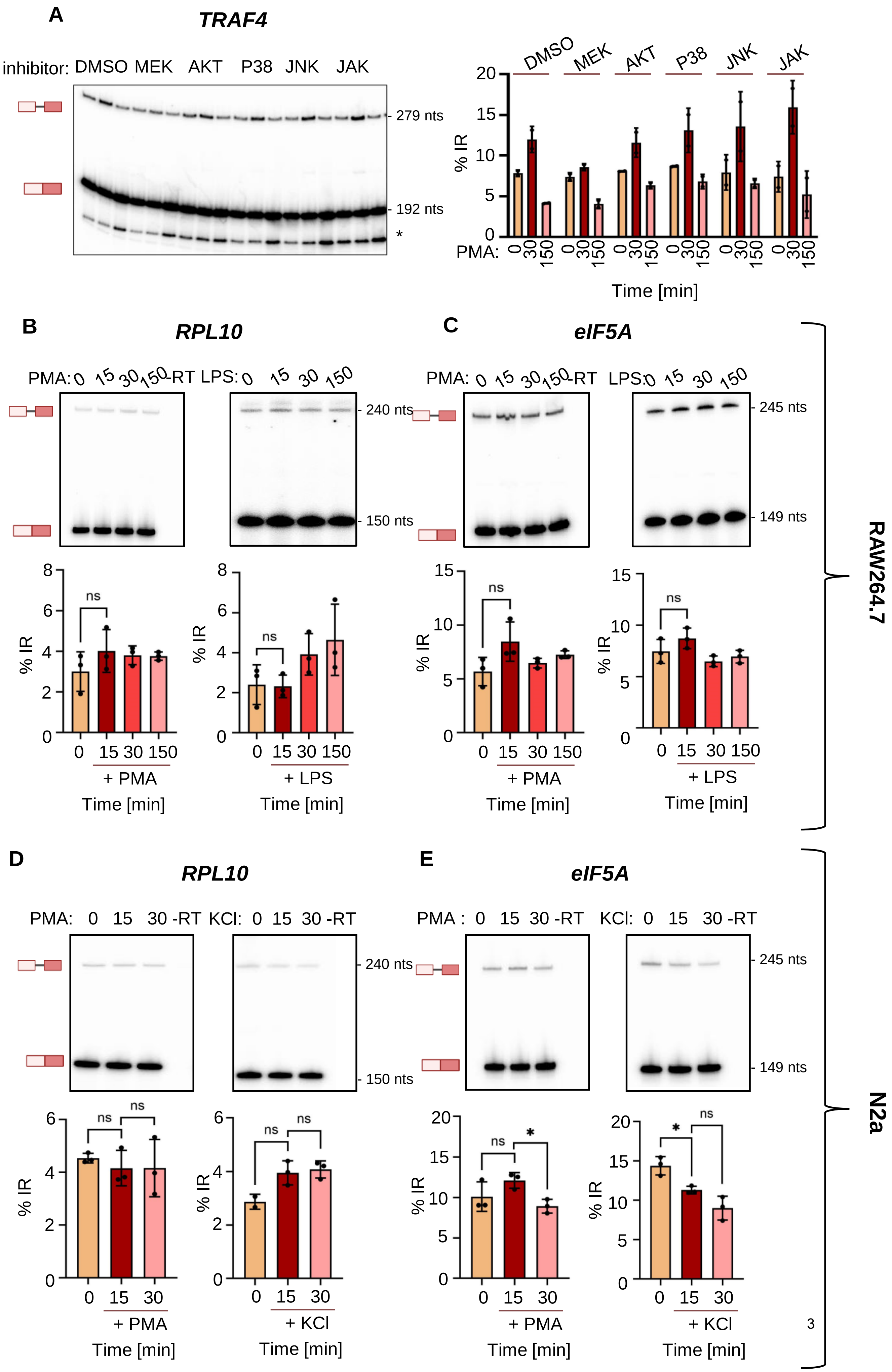
A
TRAF4
inhibitor: DMSO MEK AKT P38 JNK JAK
279 nts
192 nts
*
DMSO MEK AKT P38 JNK JAK
% IR
PMA: 0 30 150
Time [min]
B
RPL10
PMA: 0 15 30 150 -RT
LPS: 0 15 30 150
240 nts
150 nts
C
eIF5A
245 nts
149 nts
ns
% IR
+ PMA
+ LPS
Time [min]
RAW264.7
D
RPL10
PMA: 0 15 30 -RT
KCl: 0 15 30 -RT
E
eIF5A
*
+ KCl
N2a

3

Appendix Fig. S2. IES is specific to T cells

A. MEK inhibition prevents IES in *TRAF4*. Experiment as in Figs. 4A, B, * - degradation product

B, C. RAW264.7 cells were stimulated with either 20 ng/ml of PMA (left) or 0.1 µg/ml of LPS for the indicated time. Analysis as in Fig. 1D (mean ± SD, n = 3, -RT; without reverse transcriptase).

D, E. N2a cells were stimulated by either 20ng/ml of PMA (left) or 60mM of KCl (right) for indicated times (D) *RPL10*, (E) *eIF5A*. Analysis as in Fig. 1D (mean ± SD, n = 3, -RT; without reverse transcriptase).

Data information: Data in B-E are presented as mean ± SD, n = 3, ns: non-significant, *$p<0.05$ (student's unpaired t-test).

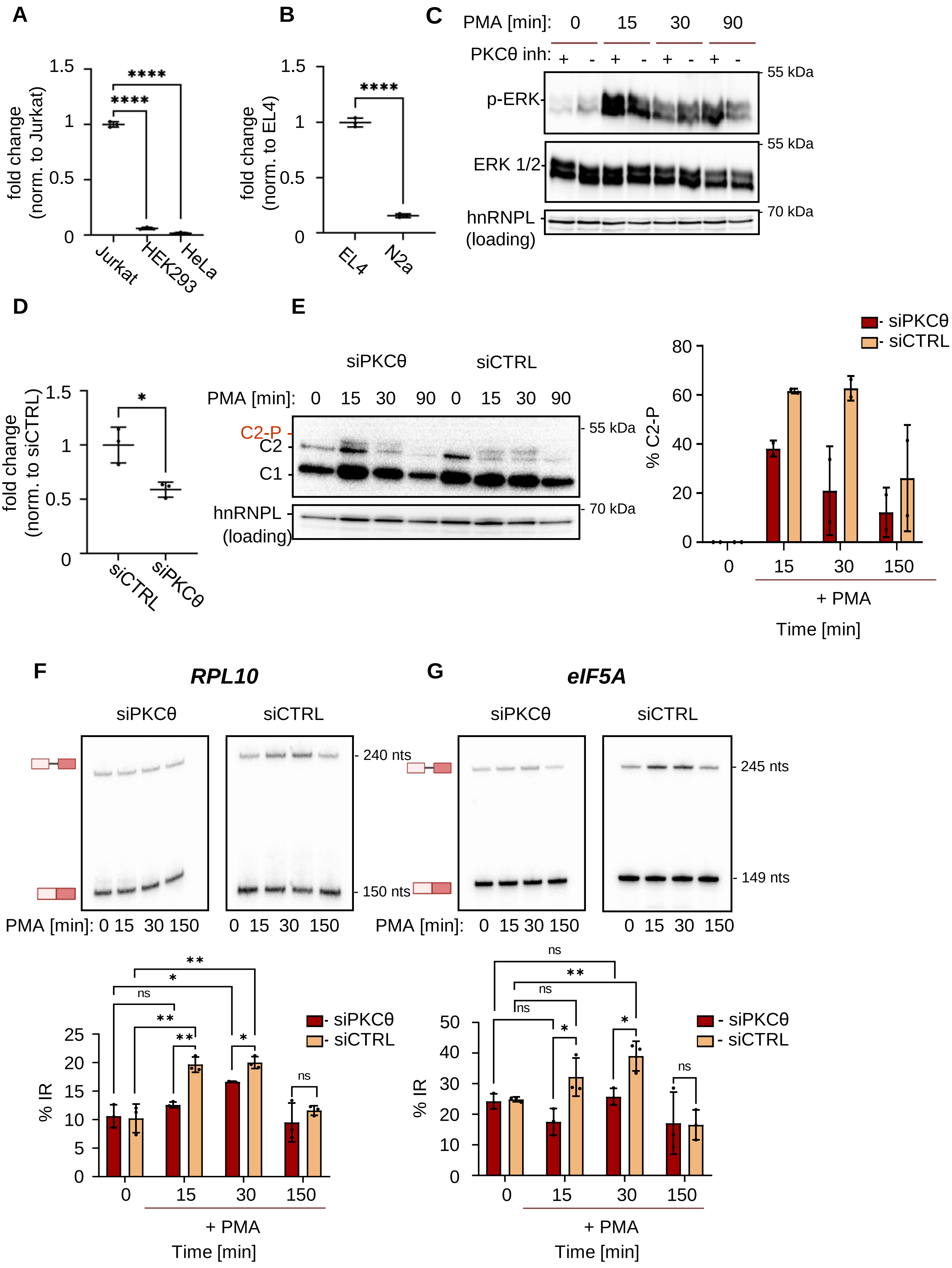
A
fold change (norm. to Jurkat)
Jurkat
HEK293
HeLa
B
fold change (norm. to EL4)
EL4
N2a
C
PMA [min]: 0 15 30 90
PKCθ inh: + - + - + - + -
p-ERK
ERK 1/2
hnRNPL (loading)
55 kDa
55 kDa
70 kDa
D
fold change (norm. to siCTRL)
siCTRL
siPKCθ
E
siPKCθ
siCTRL
PMA [min]: 0 15 30 90 0 15 30 90
C2-P
C2
C1
hnRNPL (loading)
55 kDa
70 kDa
% C2-P
siPKCθ
siCTRL
+ PMA
Time [min]
F
RPL10
siPKCθ
siCTRL
240 nts
150 nts
PMA [min]: 0 15 30 150
% IR
+ PMA
Time [min]
G
eIF5A
siPKCθ
siCTRL
245 nts
149 nts
PMA [min]: 0 15 30 150
% IR
+ PMA
Time [min]

Appendix Fig. S3. PKCθ regulates hnRNPC2-mediated IES

A. PKCθ expression in human Jurkat cells, HEK293, and HeLa cells was analyzed by RT-qPCR. mRNA expression is relative to hHPRT. PKCθ is highly expressed in Jurkat cells and is barely expressed in HEK293 and HeLa cells. Statistical significance is indicated by asterisks.

B. PKCθ expression in mouse EL4 cells and N2a cells was analyzed by RT-qPCR. mRNA expression is relative to mHPRT. PKCθ is highly expressed in EL4 cells, and is barely expressed in N2a. Statistical significance is indicated by asterisks.

C. Jurkat cells were PMA-stimulated for the indicated time. Cells were treated with PKCθ inhibitor 30 minutes before PMA-treatment. Total protein was extracted. A representative blot shows PKCθ did not block ERK1/2 activation after PMA stimulation. hnRNPL serves as loading control, n = 3.

D. Knockdown of PKCθ by siRNA was investigated by RT-qPCR. mRNA expression is relative to hHPRT. Statistical significance is indicated by asterisks.

E. PKCθ knockdown reduces hnRNPC2 phosphorylation. Jurkat cells as in E, F were analyzed by Western blot (top). hnRNPL serves as loading control; Bottom: quantification of blots as above (% hnRNPC2 phosphorylation of total hnRNPC2; mean ± SD, n = 2).

F, G. PKCθ knockdown abolishes IES. Jurkat cells were treated with siRNA against PKCθ and siCTRL. 48 hours post transfection cells were stimulated with PMA, chromatin-associated RNA was investigated for *RPL10* (F) and *eIF5A* (G) IES by radioactive, splicing-sensitive RT-PCR. Bottom: quantification of above data, % IR).

Data information: Data in A, B, D-F are presented as mean ± SD, n = 3, ns: non-significant, *p<0.05; **p<0.01, ***p<0.001, ****p<0.0001 (student's unpaired t-test).
