# Supplementary figures and images for: Immediate early splicing controls translation in activated T-cells and is mediated by hnRNPC2 phosphorylation
Source: EMBO J. 2025 Feb 13;44(6):1692–723. doi: 10.1038/s44318-025-00374-8 (PMC11914300; doi:10.1038/s44318-025-00374-8)

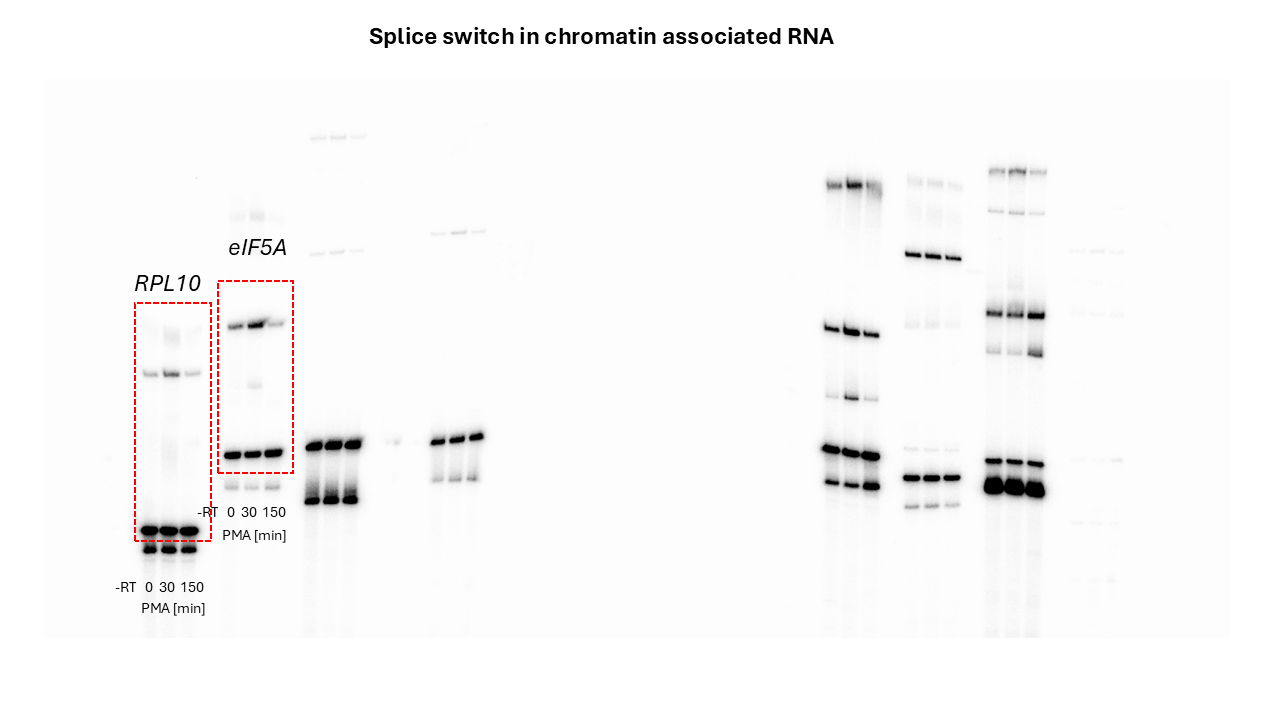

Supplement: Supplementary file 5 — Source data Fig. 1 [file 44318_2025_374_MOESM5_ESM.zip › EMBOJ-2024-118552_Source data_Fig. 1/1D/1D.tif]

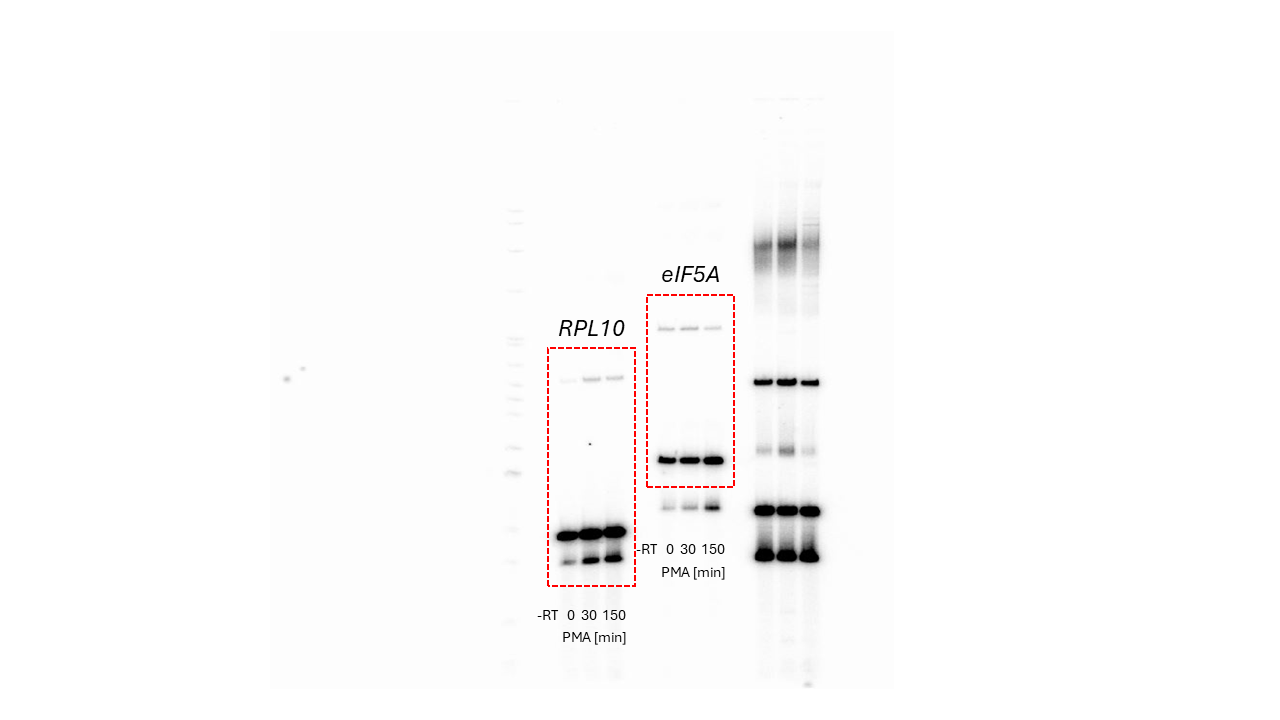

Supplement: Supplementary file 5 — Source data Fig. 1 [file 44318_2025_374_MOESM5_ESM.zip › EMBOJ-2024-118552_Source data_Fig. 1/1G/1G.tif]

## Splice switch in chromatin associated RNA (in presence of Chx)

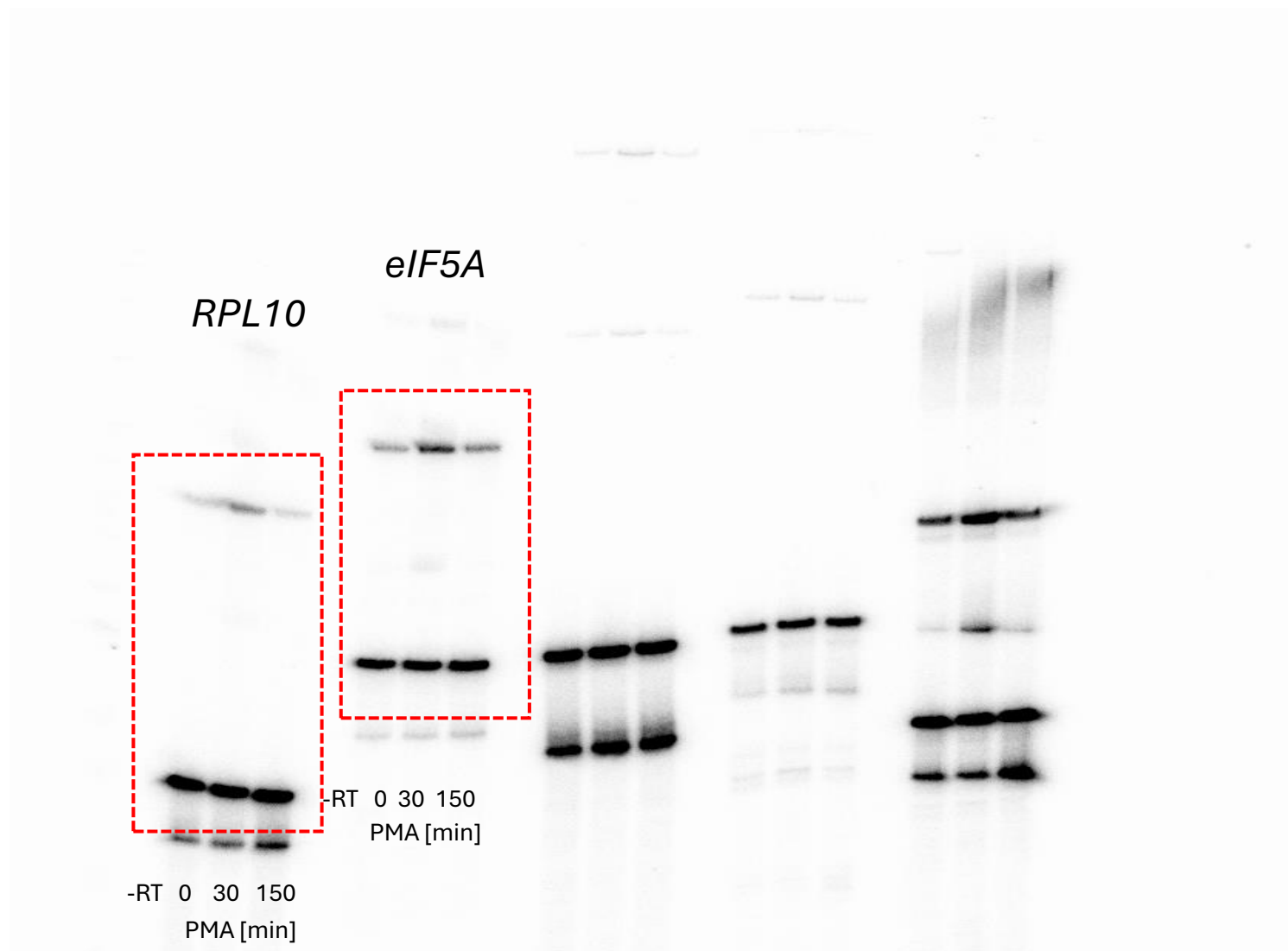

Supplement: Supplementary file 5 — Source data Fig. 1 [file 44318_2025_374_MOESM5_ESM.zip › EMBOJ-2024-118552_Source data_Fig. 1/1E/1E.pdf]

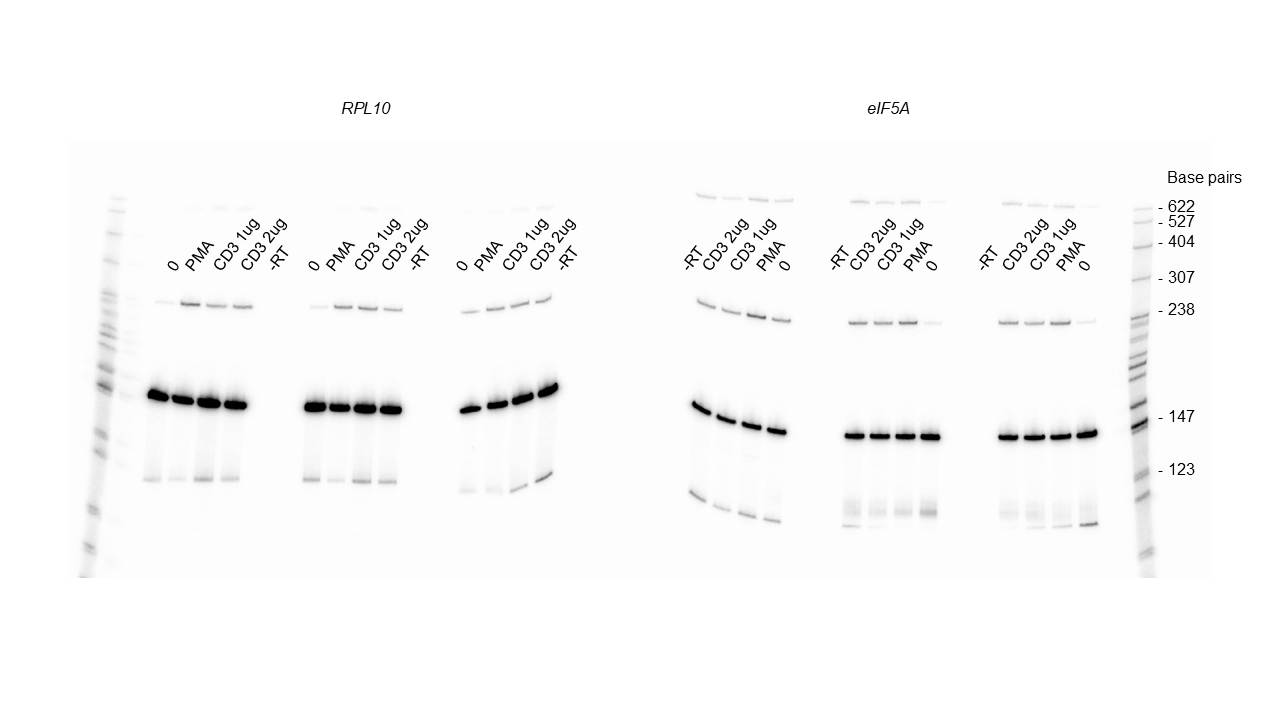

Supplement: Supplementary file 5 — Source data Fig. 1 [file 44318_2025_374_MOESM5_ESM.zip › EMBOJ-2024-118552_Source data_Fig. 1/1F/1F.tif]

*RPL10*

*eIF5A*

Base pairs

- 622  
- 527  
- 404  
- 307  
- 238  
- 147  
- 123

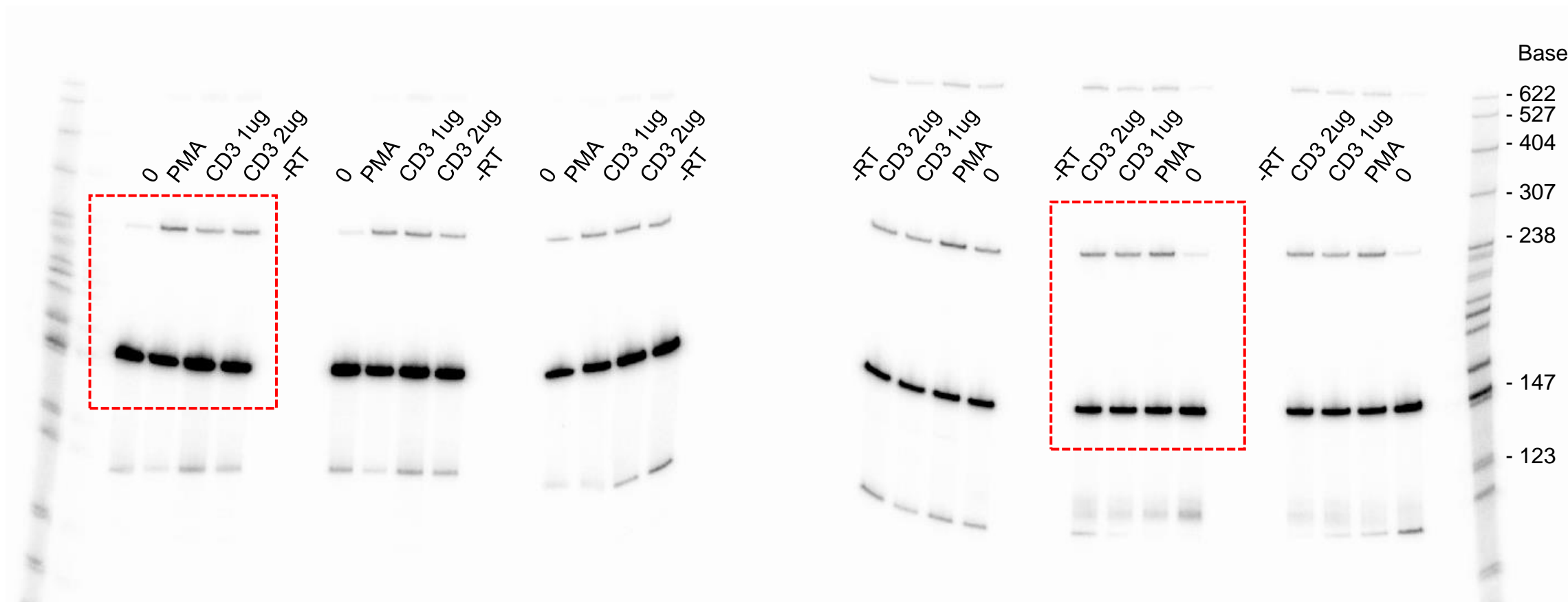

Supplement: Supplementary file 5 — Source data Fig. 1 [file 44318_2025_374_MOESM5_ESM.zip › EMBOJ-2024-118552_Source data_Fig. 1/1F/1F.pdf]

## Splice switch in chromatin associated RNA

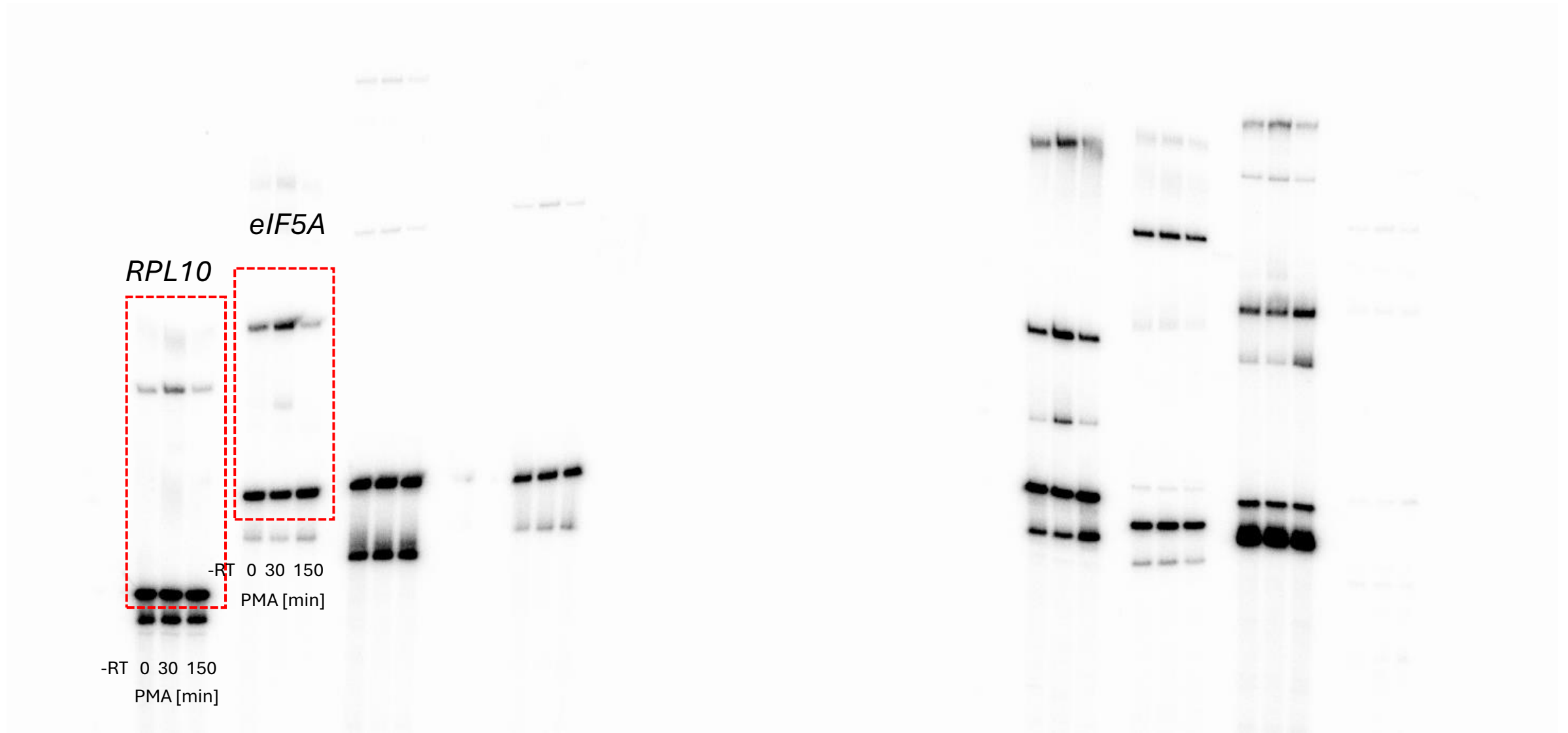

Supplement: Supplementary file 5 — Source data Fig. 1 [file 44318_2025_374_MOESM5_ESM.zip › EMBOJ-2024-118552_Source data_Fig. 1/1D/1D.pdf]

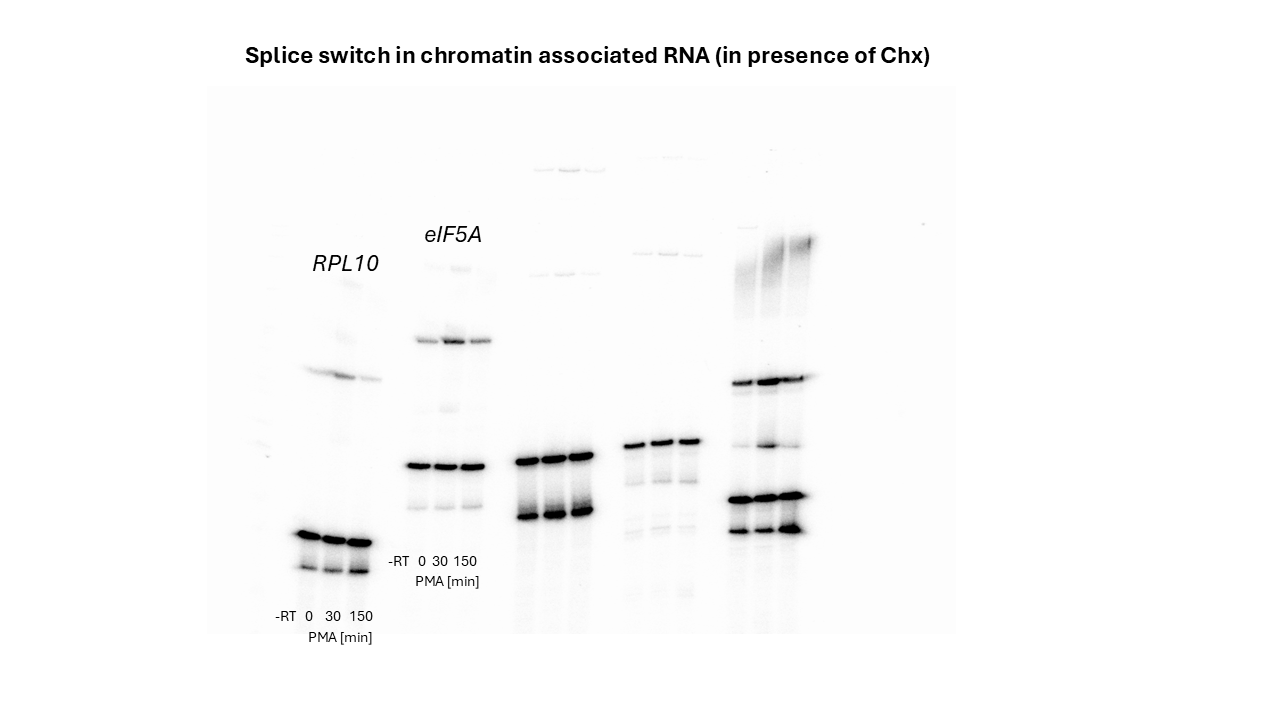

Supplement: Supplementary file 5 — Source data Fig. 1 [file 44318_2025_374_MOESM5_ESM.zip › EMBOJ-2024-118552_Source data_Fig. 1/1E/1E.tif]

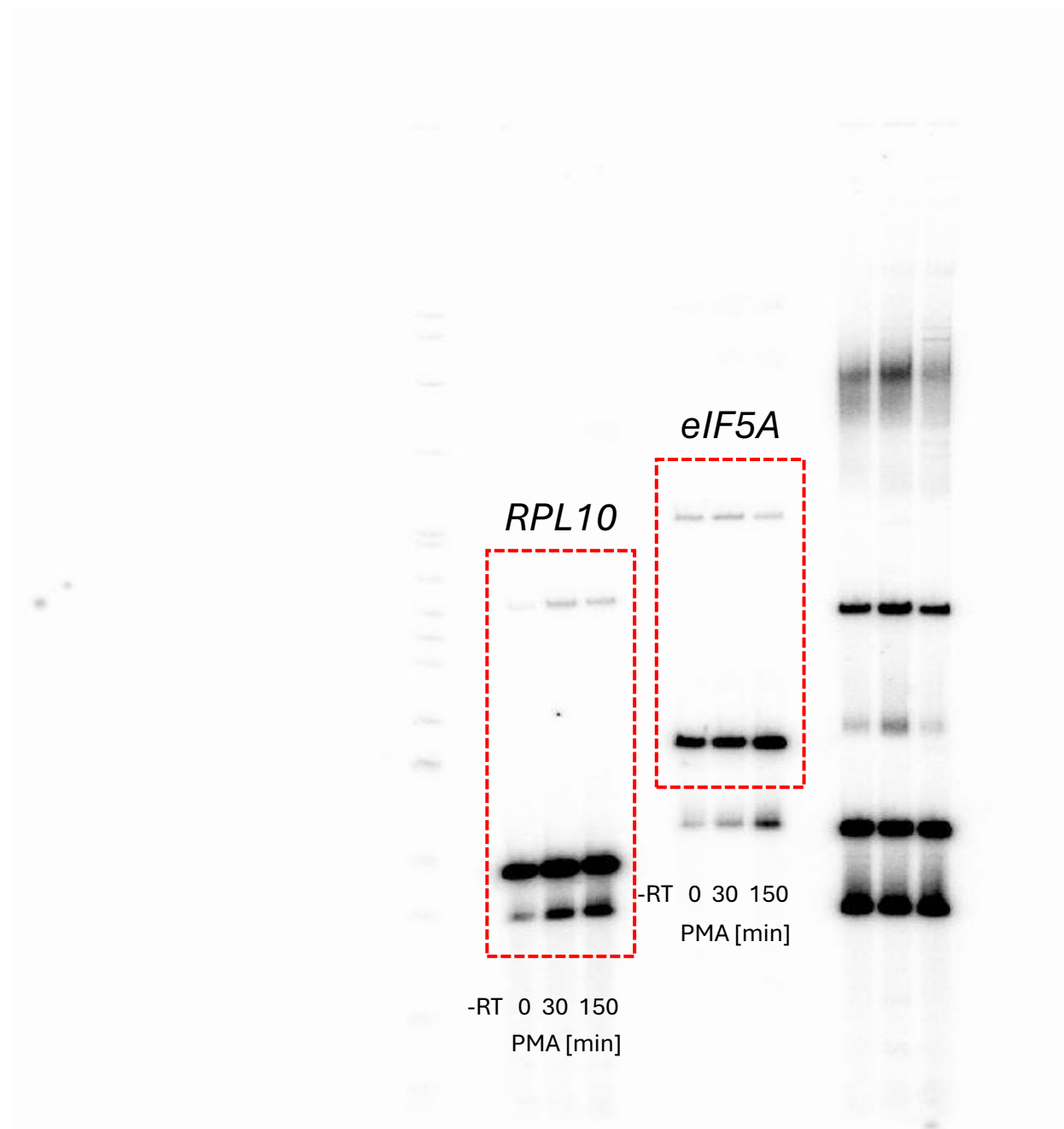

Supplement: Supplementary file 5 — Source data Fig. 1 [file 44318_2025_374_MOESM5_ESM.zip › EMBOJ-2024-118552_Source data_Fig. 1/1G/1G.pdf]

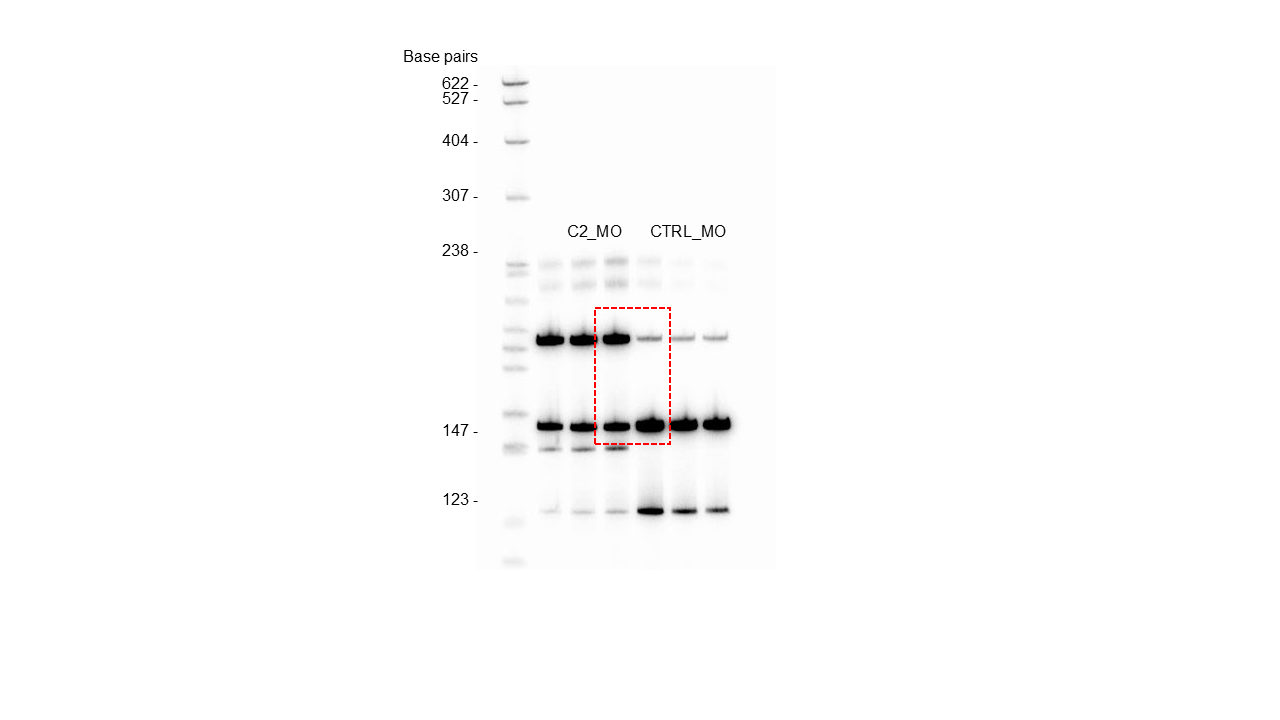

Supplement: Supplementary file 6 — Source data Fig. 2 [file 44318_2025_374_MOESM6_ESM.zip › EMBOJ-2024-118552_Source data_Fig. 2/2E/2E.tif]

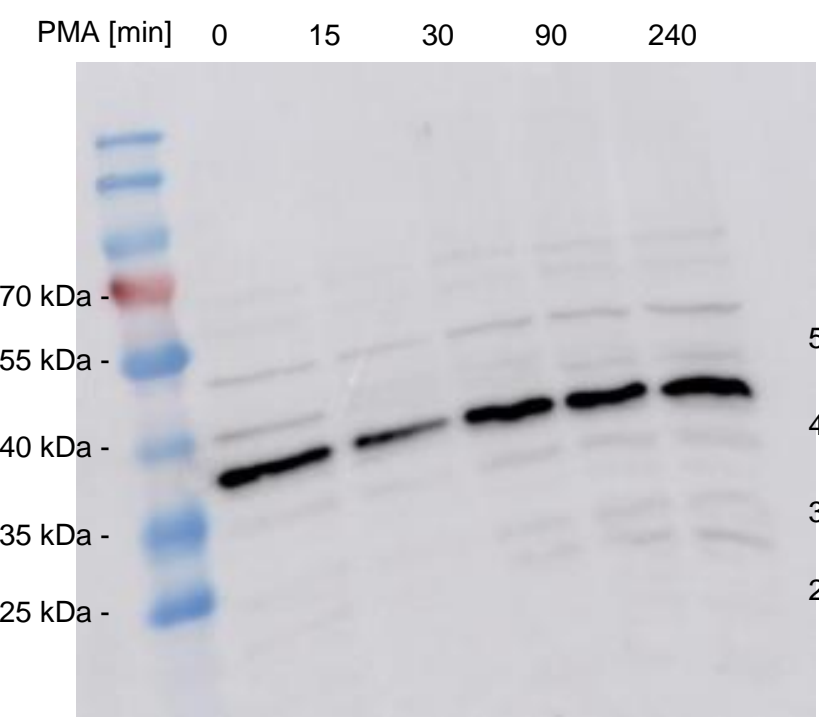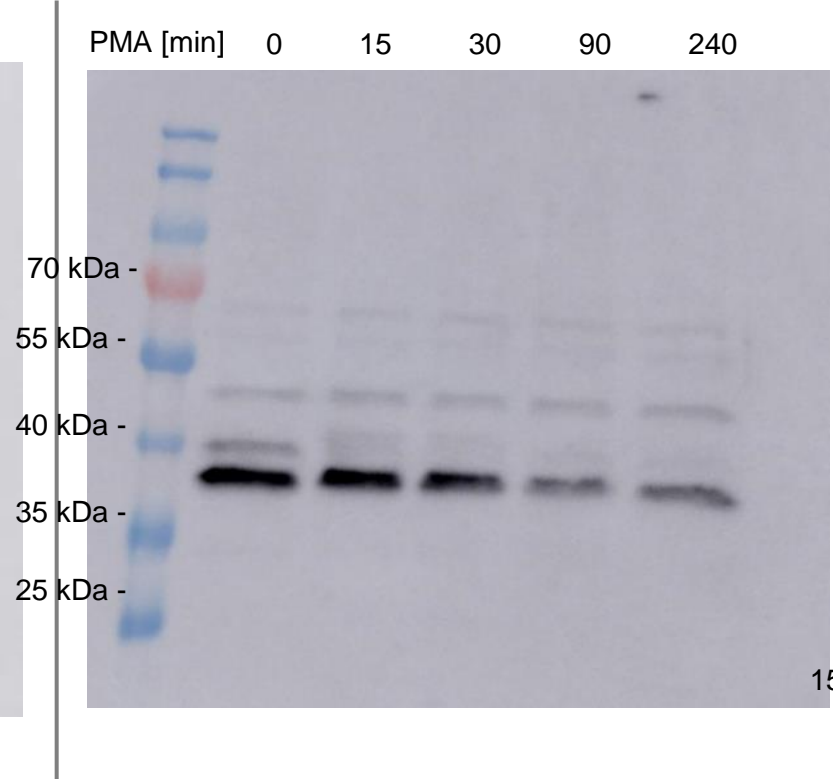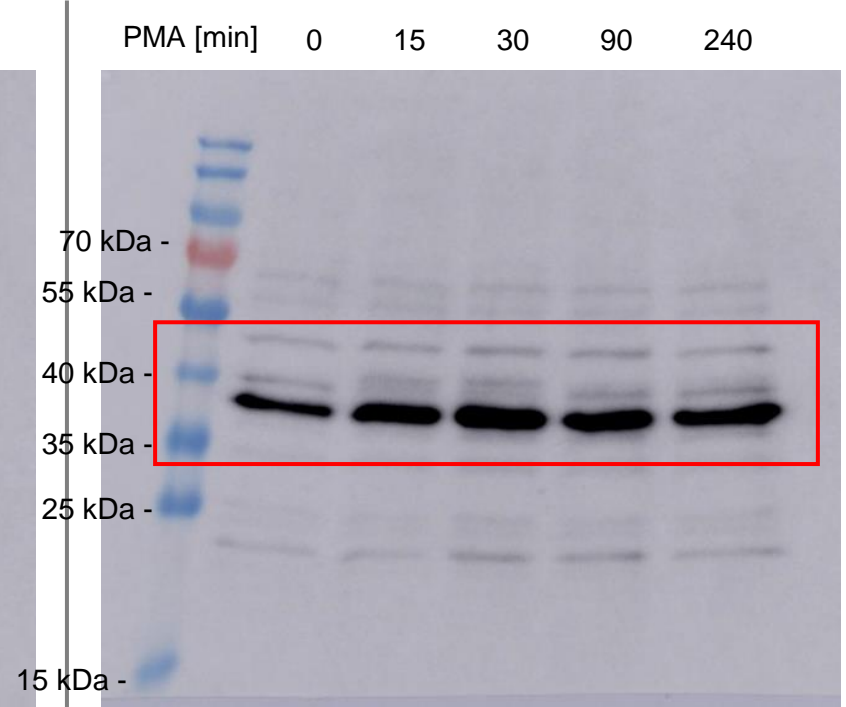

Supplement: Supplementary file 6 — Source data Fig. 2 [file 44318_2025_374_MOESM6_ESM.zip › EMBOJ-2024-118552_Source data_Fig. 2/2C/2C.pdf]

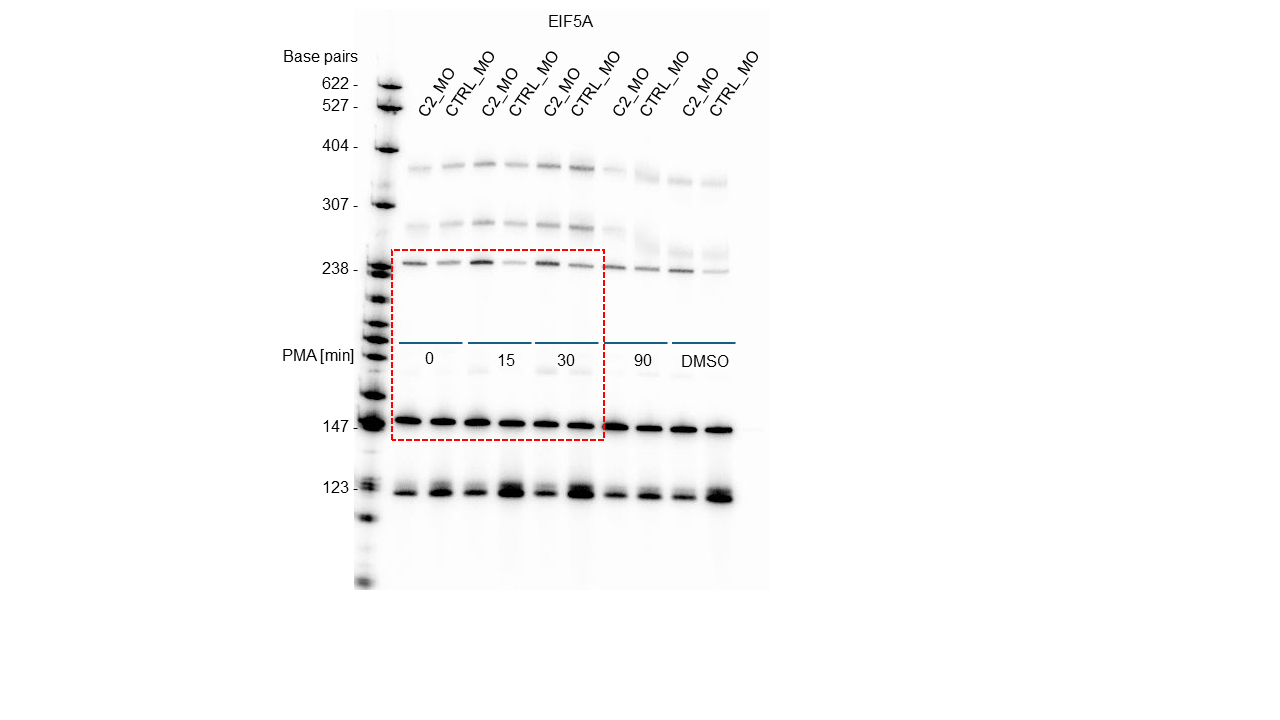

Supplement: Supplementary file 6 — Source data Fig. 2 [file 44318_2025_374_MOESM6_ESM.zip › EMBOJ-2024-118552_Source data_Fig. 2/2I/2I.tif]

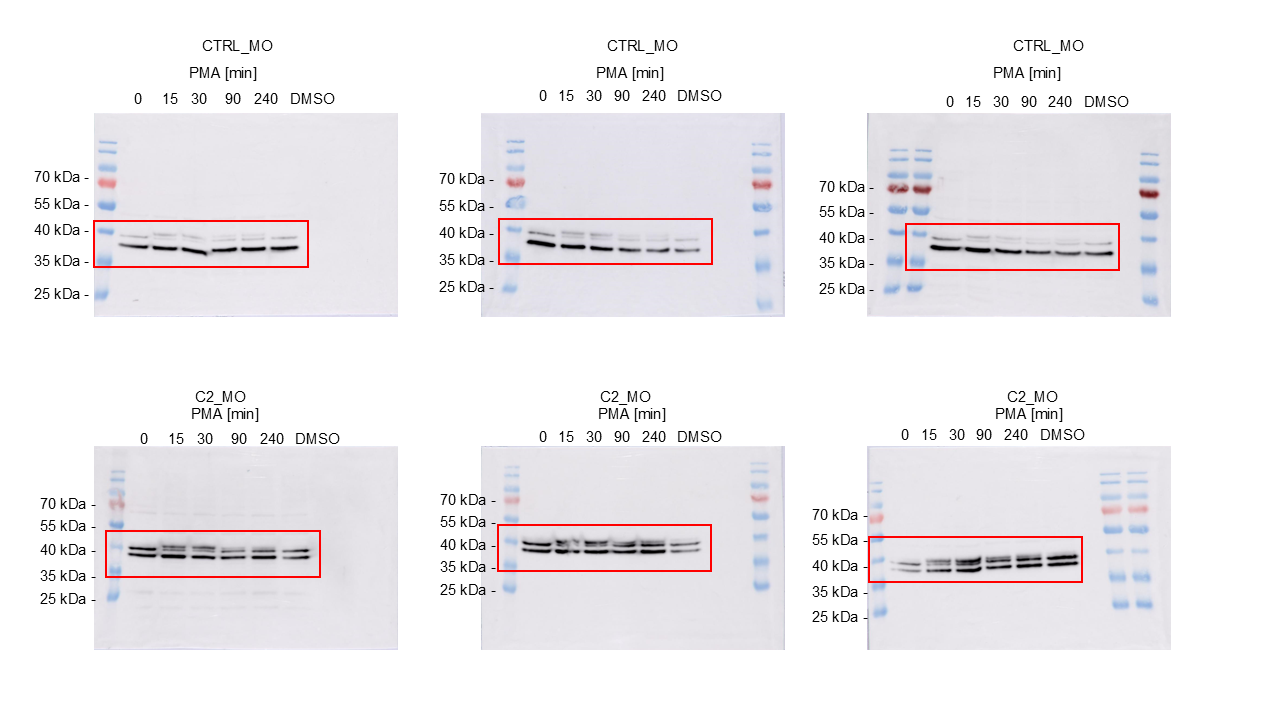

Supplement: Supplementary file 6 — Source data Fig. 2 [file 44318_2025_374_MOESM6_ESM.zip › EMBOJ-2024-118552_Source data_Fig. 2/2G/2G.tif]

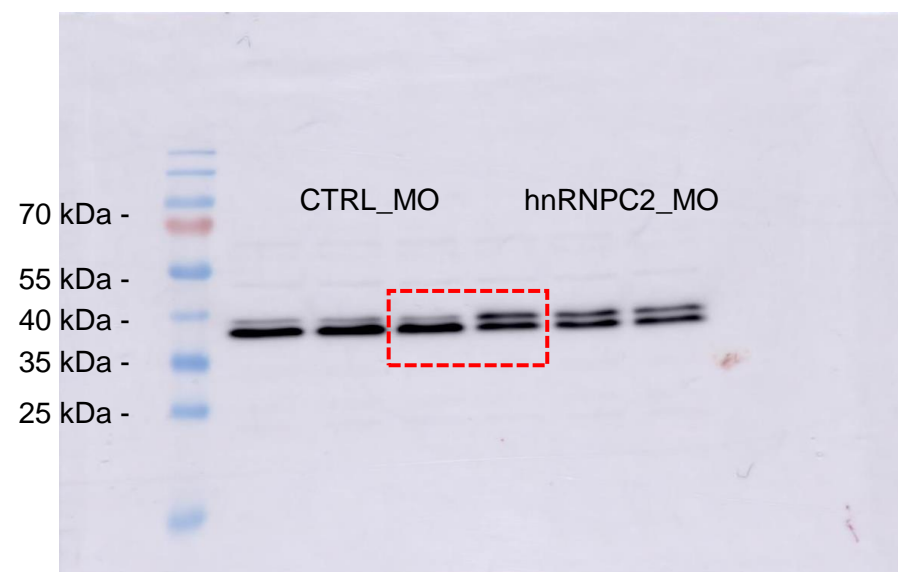

Supplement: Supplementary file 6 — Source data Fig. 2 [file 44318_2025_374_MOESM6_ESM.zip › EMBOJ-2024-118552_Source data_Fig. 2/2F/2F.pdf]

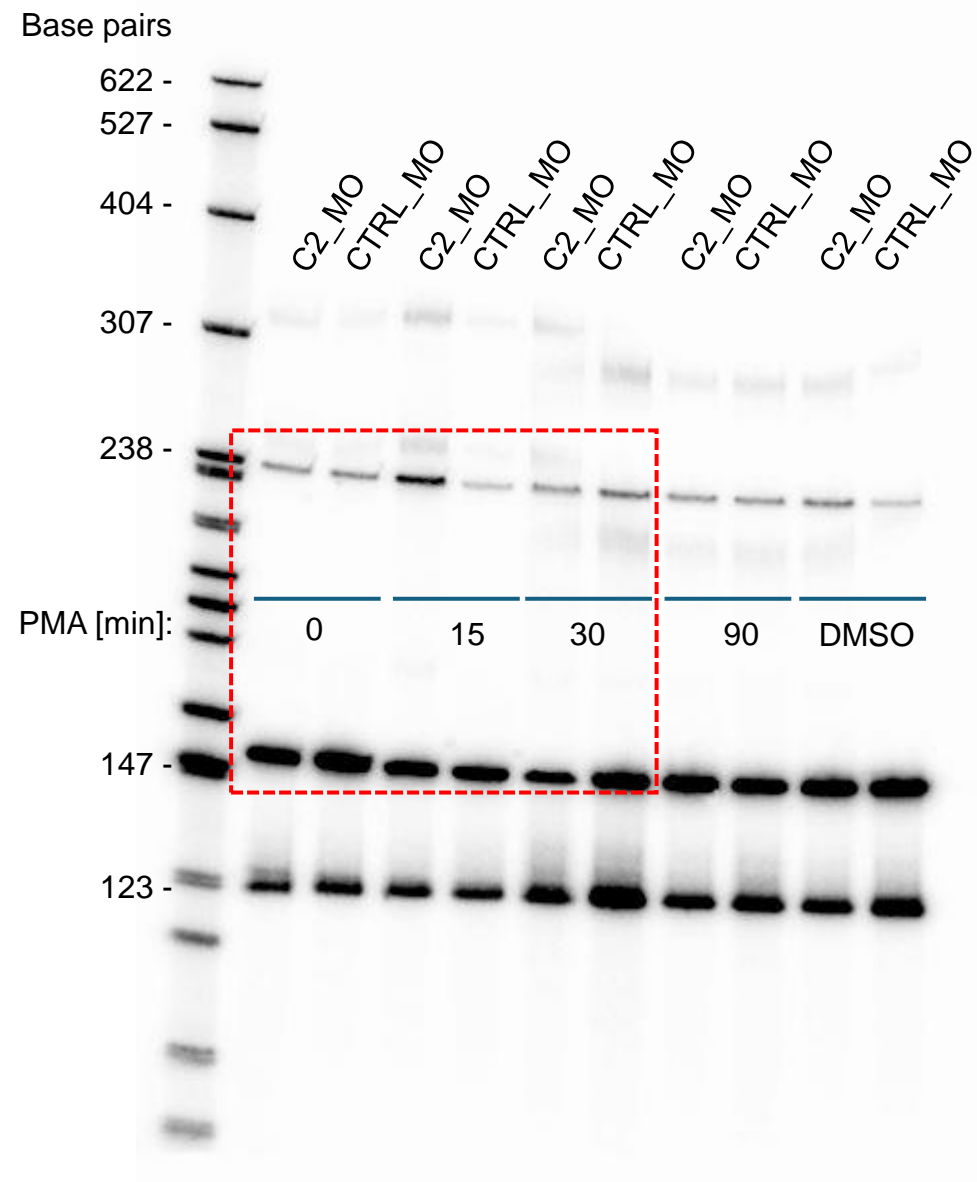

Supplement: Supplementary file 6 — Source data Fig. 2 [file 44318_2025_374_MOESM6_ESM.zip › EMBOJ-2024-118552_Source data_Fig. 2/2H/2H.pdf]

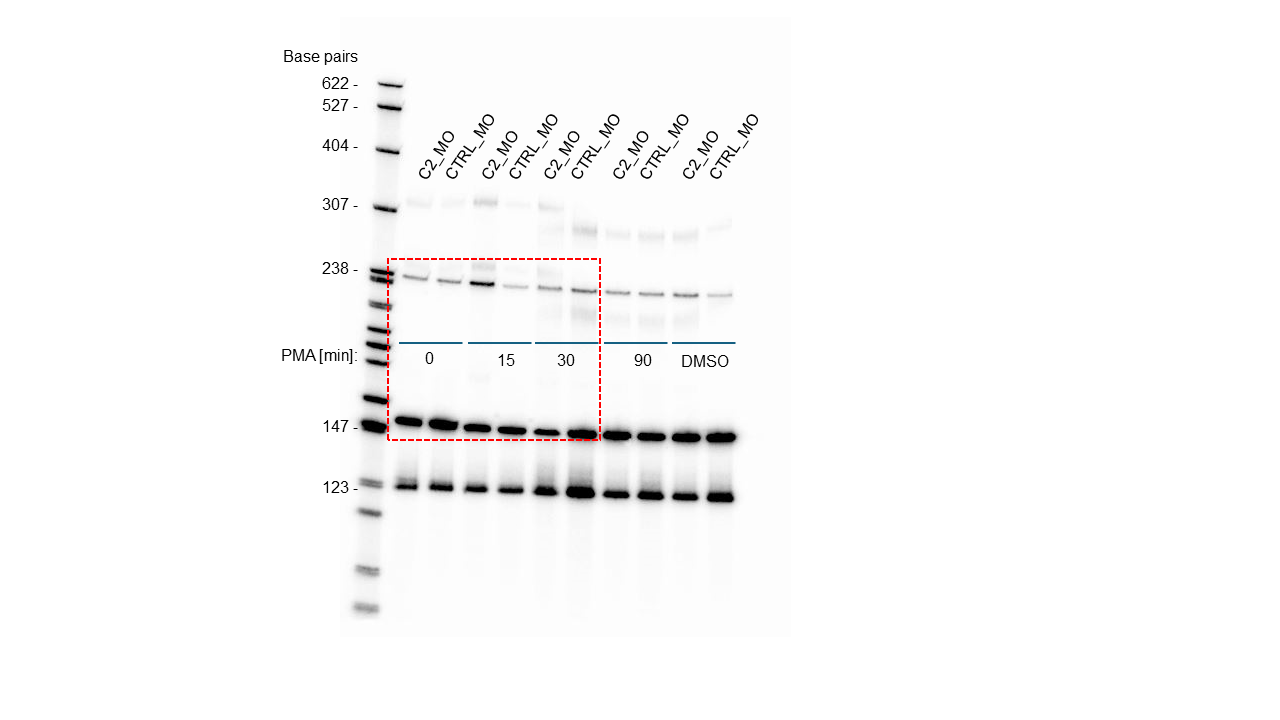

Supplement: Supplementary file 6 — Source data Fig. 2 [file 44318_2025_374_MOESM6_ESM.zip › EMBOJ-2024-118552_Source data_Fig. 2/2H/2H.tif]

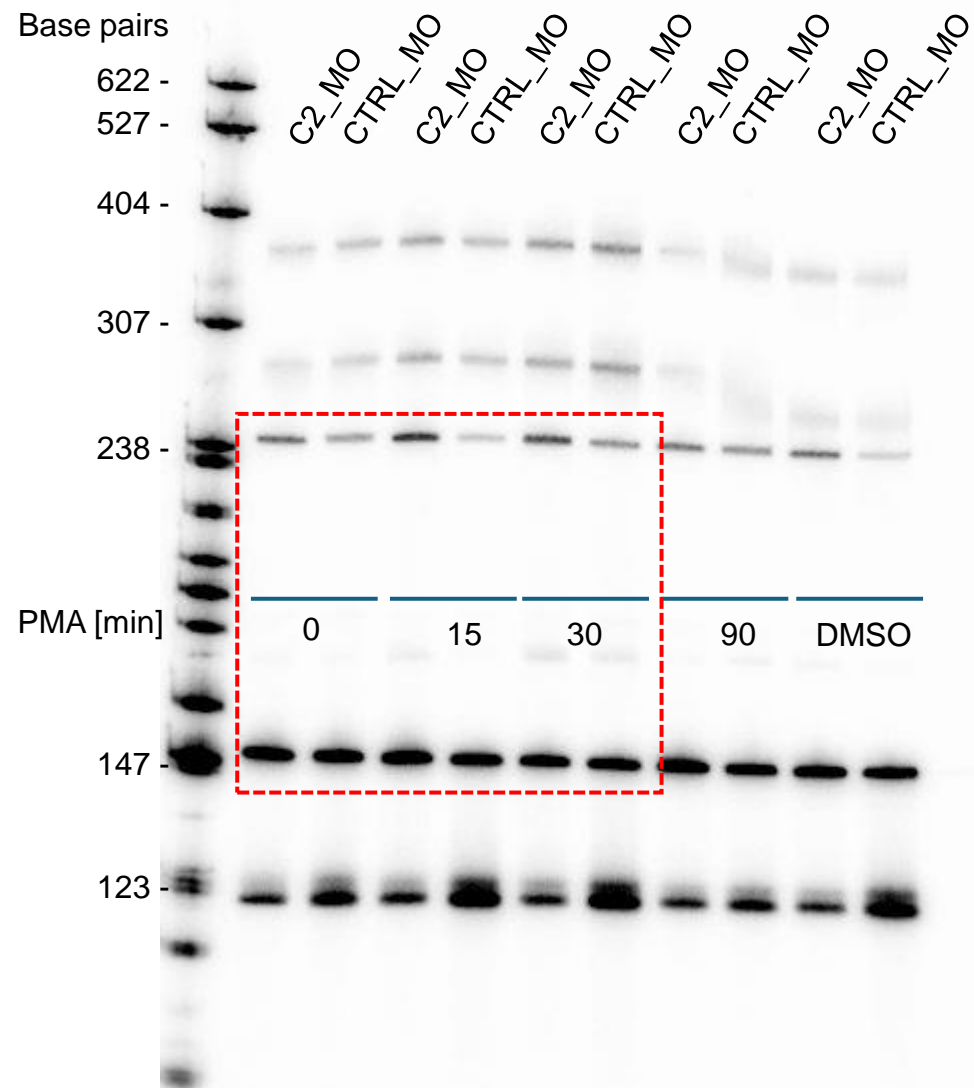

Supplement: Supplementary file 6 — Source data Fig. 2 [file 44318_2025_374_MOESM6_ESM.zip › EMBOJ-2024-118552_Source data_Fig. 2/2I/2I.pdf]

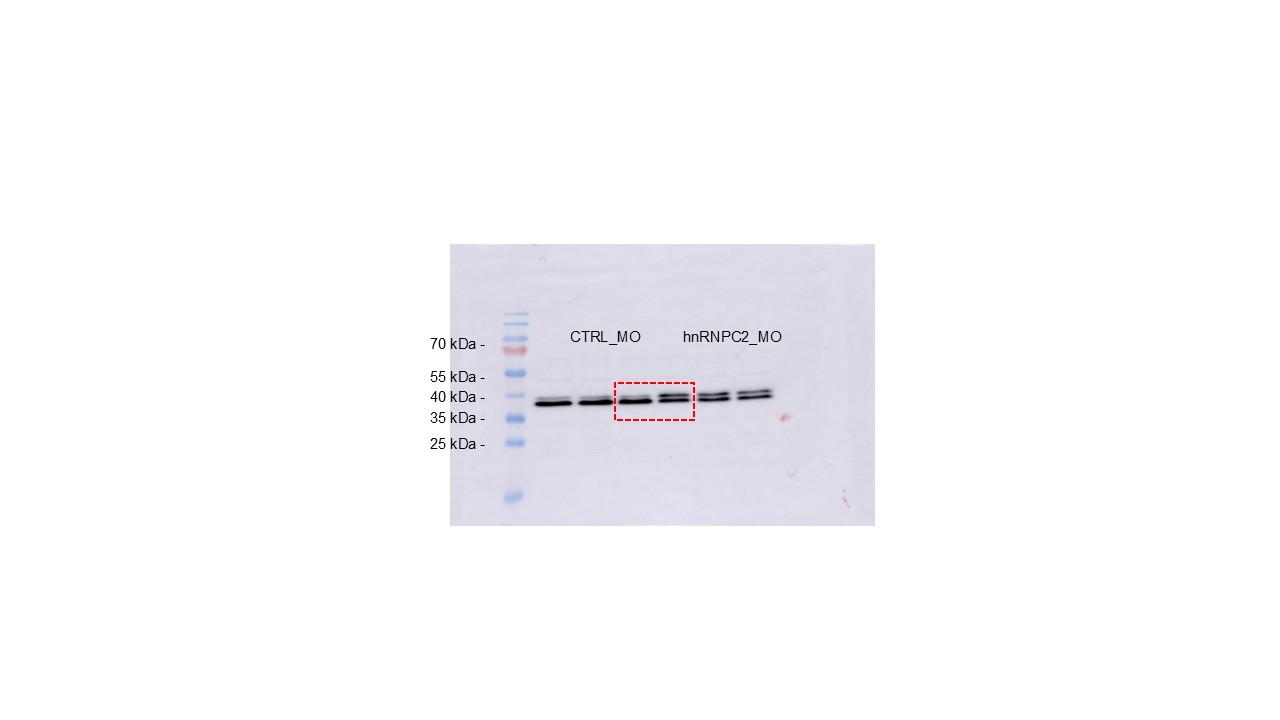

Supplement: Supplementary file 6 — Source data Fig. 2 [file 44318_2025_374_MOESM6_ESM.zip › EMBOJ-2024-118552_Source data_Fig. 2/2F/2F.tif]

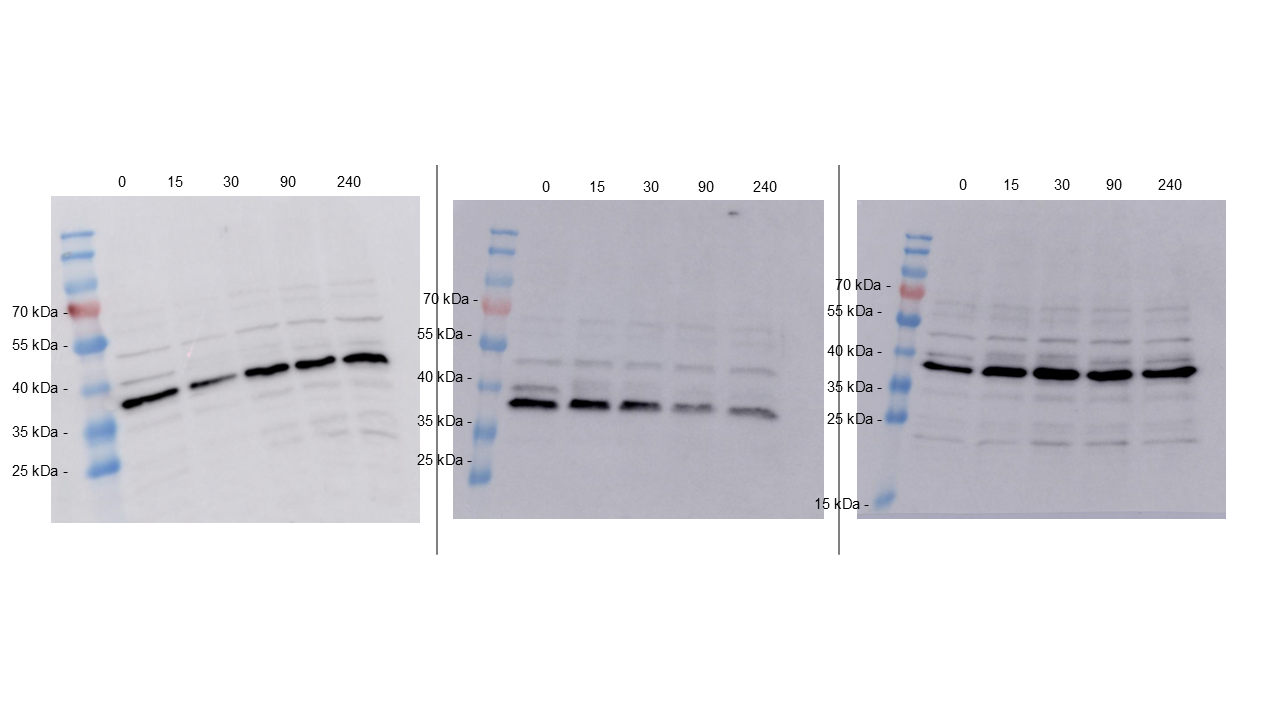

Supplement: Supplementary file 6 — Source data Fig. 2 [file 44318_2025_374_MOESM6_ESM.zip › EMBOJ-2024-118552_Source data_Fig. 2/2C/2C.TIF]

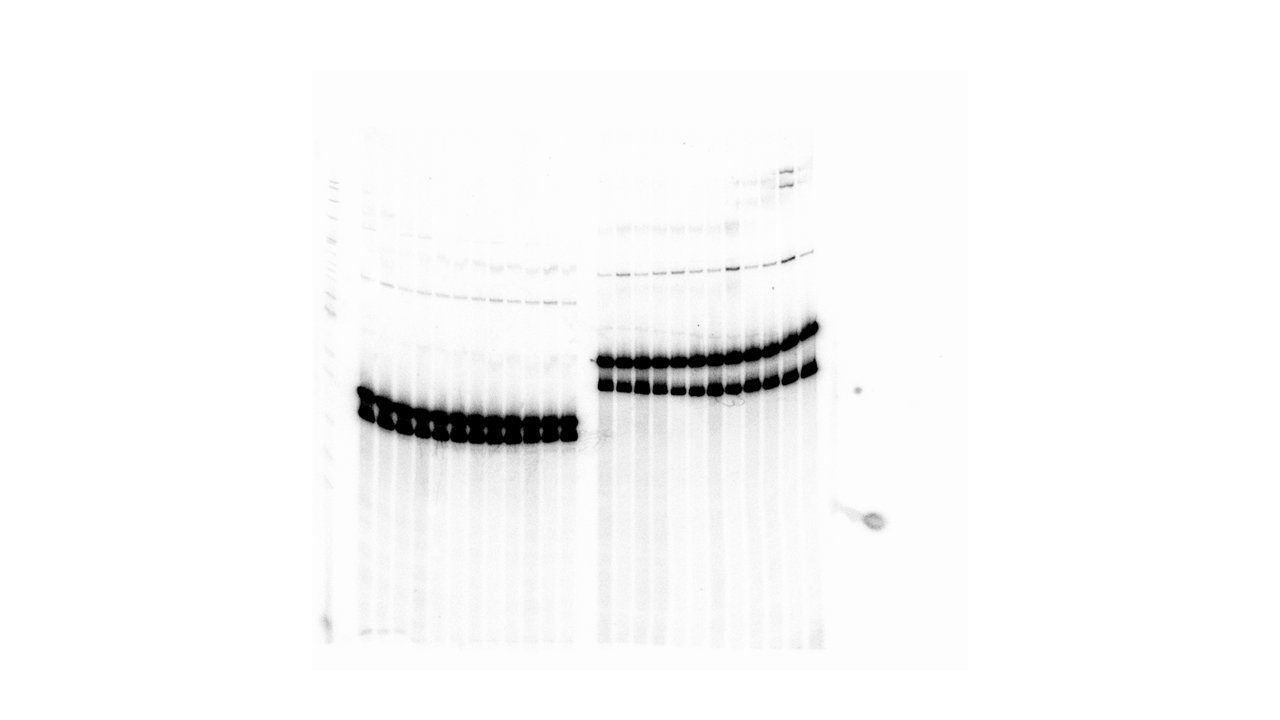

Supplement: Supplementary file 7 — Source data Fig. 3 [file 44318_2025_374_MOESM7_ESM.zip › EMBOJ-2024-118552_Source data_Fig. 3/3B/3B.tif]

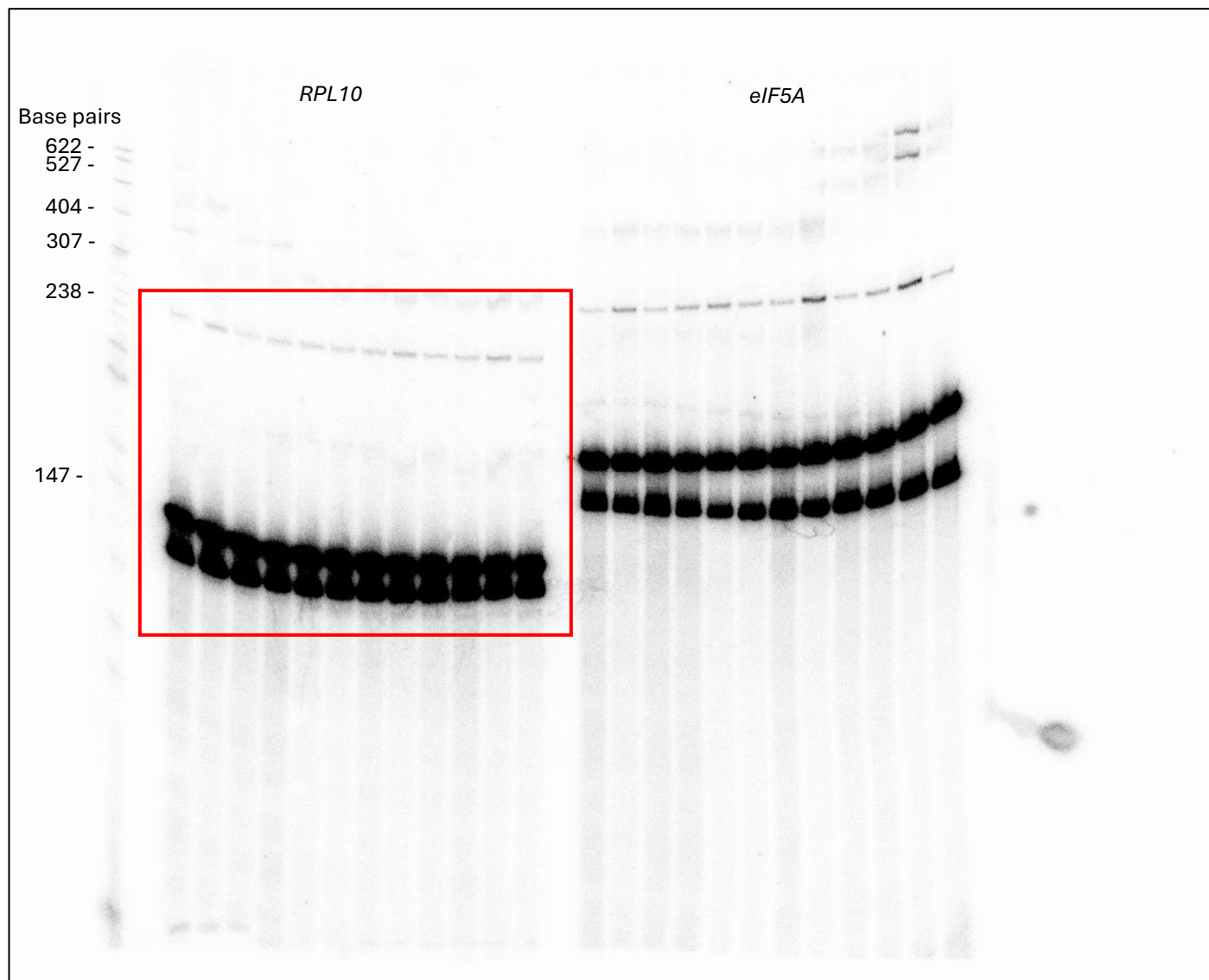

Supplement: Supplementary file 7 — Source data Fig. 3 [file 44318_2025_374_MOESM7_ESM.zip › EMBOJ-2024-118552_Source data_Fig. 3/3A/3A.pdf]

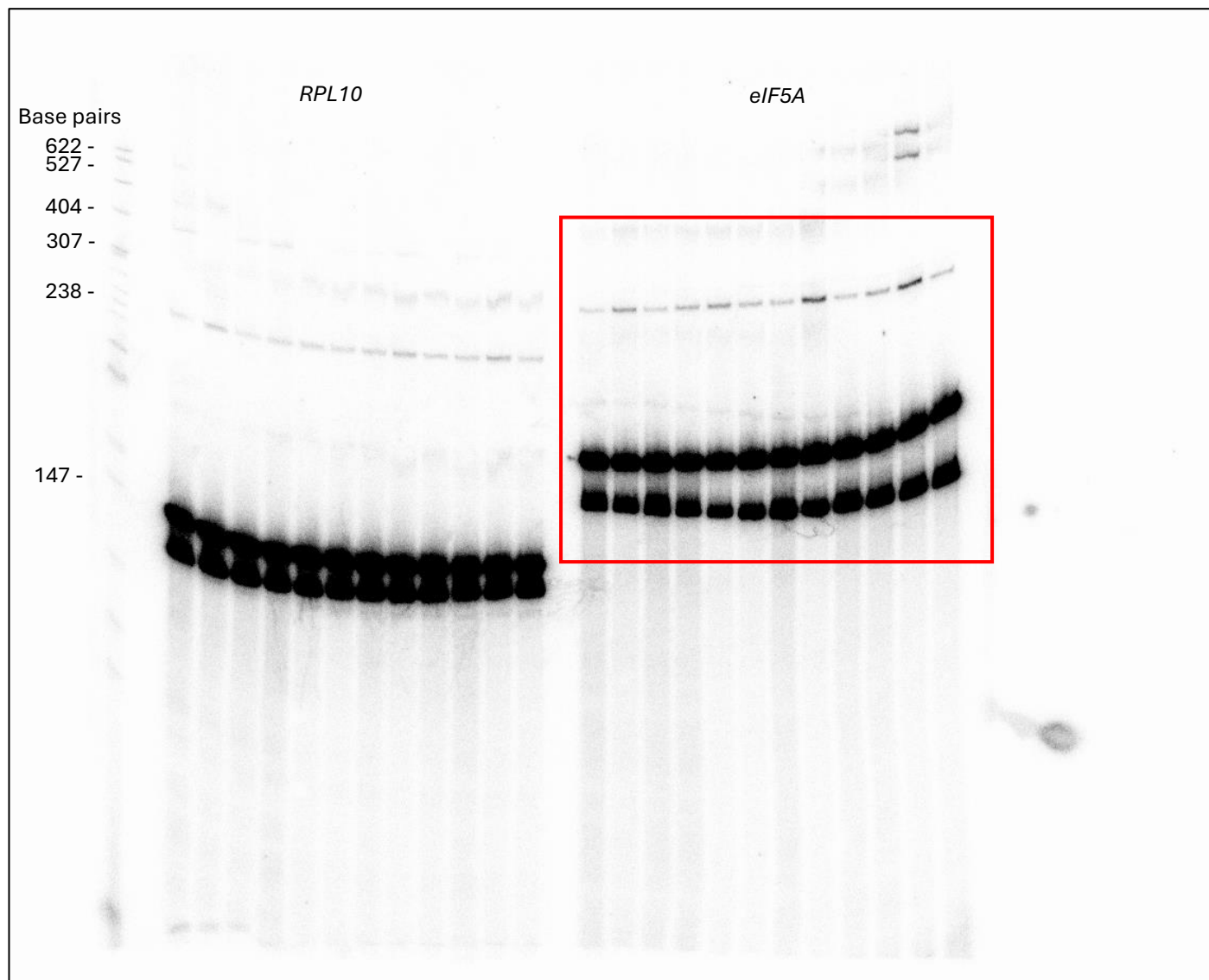

Supplement: Supplementary file 7 — Source data Fig. 3 [file 44318_2025_374_MOESM7_ESM.zip › EMBOJ-2024-118552_Source data_Fig. 3/3B/3B.pdf]

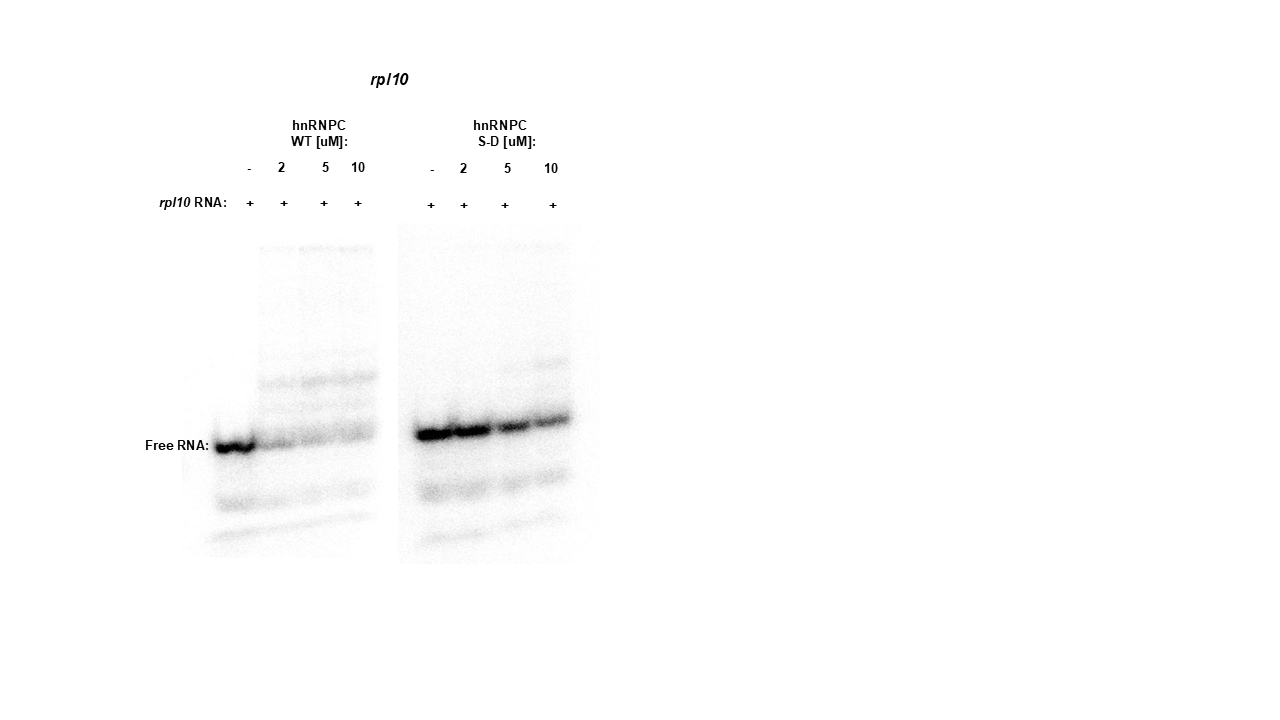

Supplement: Supplementary file 7 — Source data Fig. 3 [file 44318_2025_374_MOESM7_ESM.zip › EMBOJ-2024-118552_Source data_Fig. 3/3E/3E/Slide3.TIF]

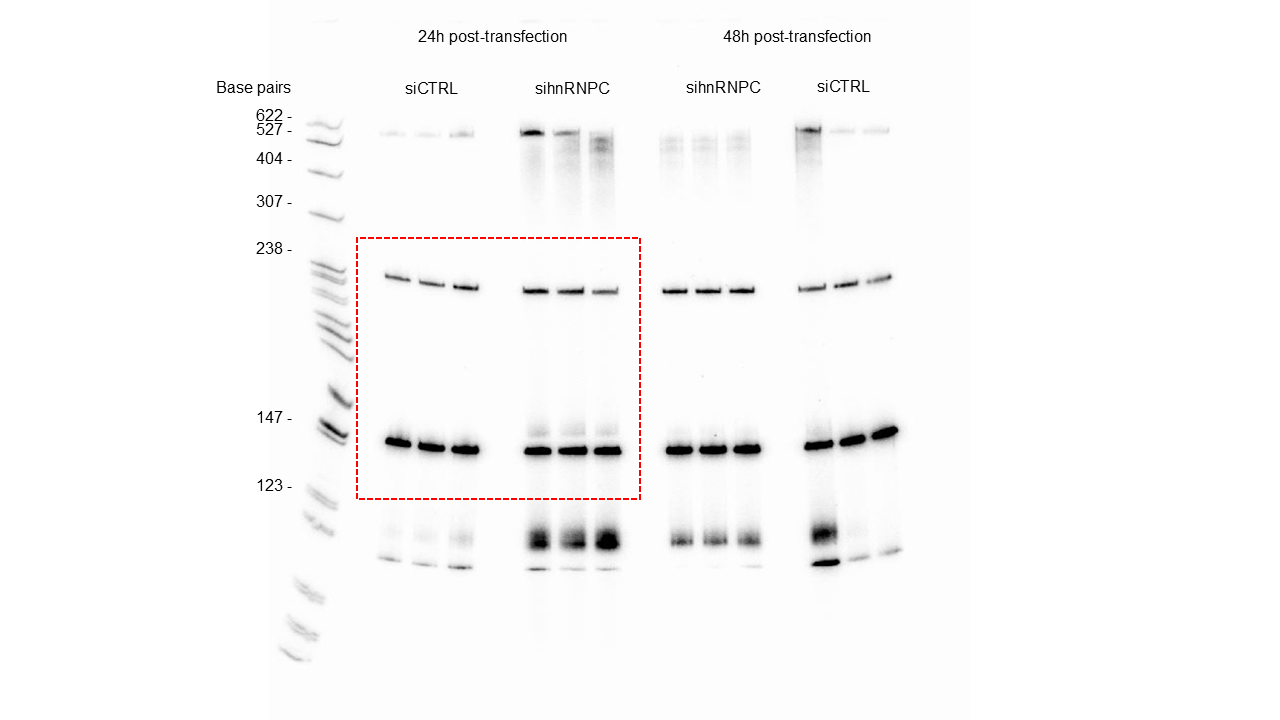

Supplement: Supplementary file 7 — Source data Fig. 3 [file 44318_2025_374_MOESM7_ESM.zip › EMBOJ-2024-118552_Source data_Fig. 3/3D/3D.tif]

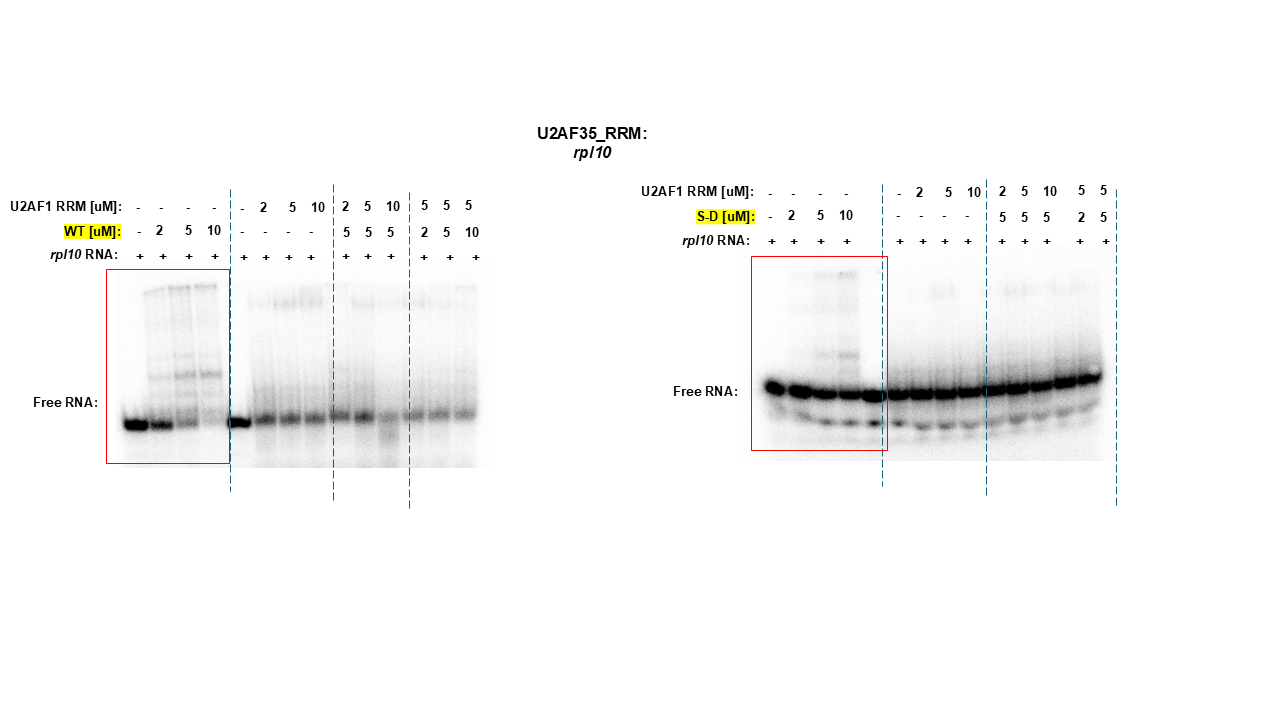

Supplement: Supplementary file 7 — Source data Fig. 3 [file 44318_2025_374_MOESM7_ESM.zip › EMBOJ-2024-118552_Source data_Fig. 3/3E/3E/Slide2.TIF]

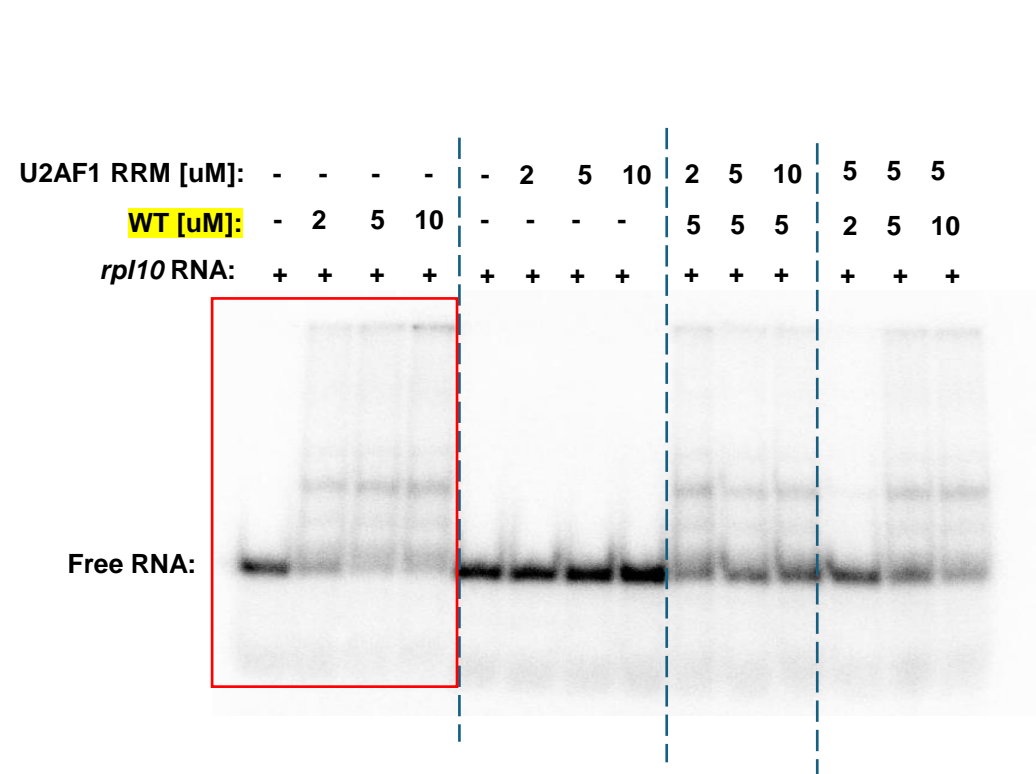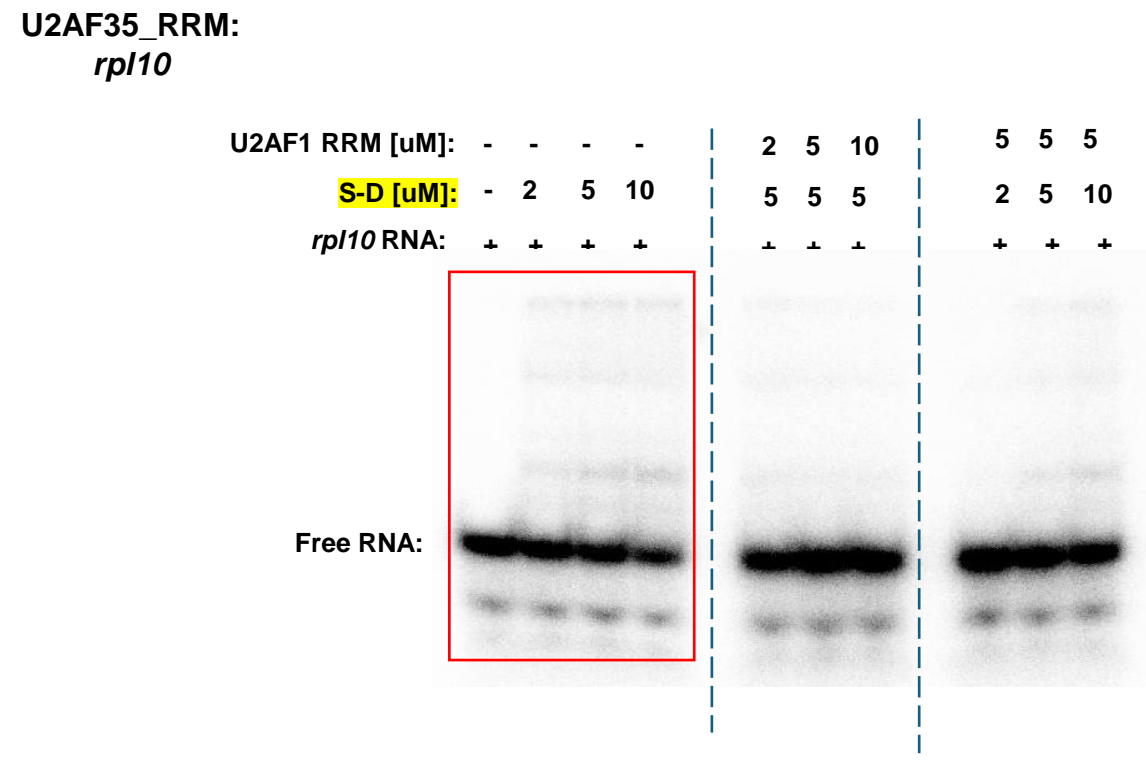

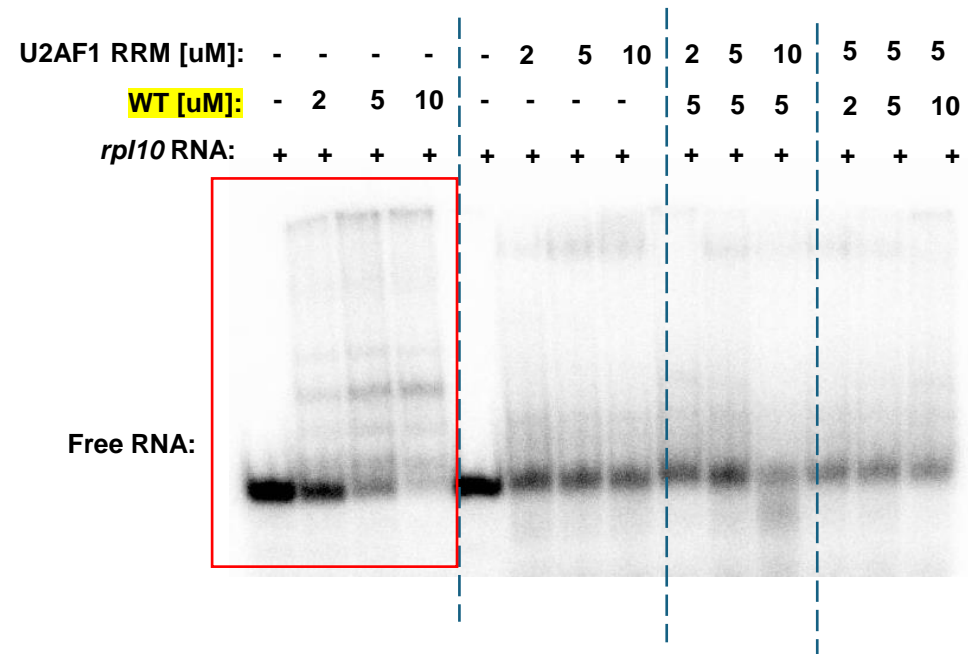

U2AF35\_RRM:  
*rpl10*

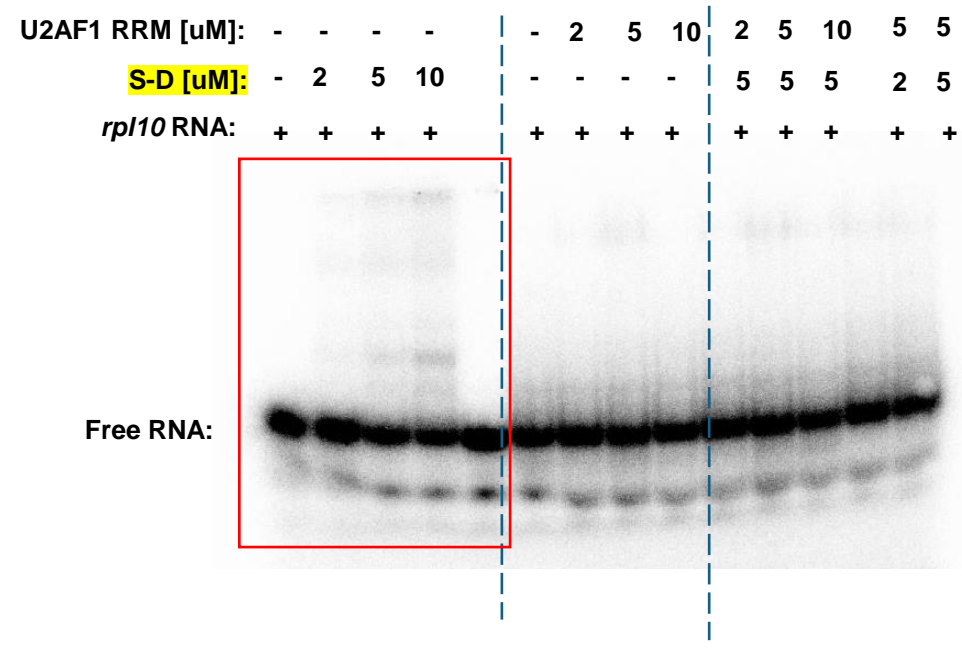

*rpl10*

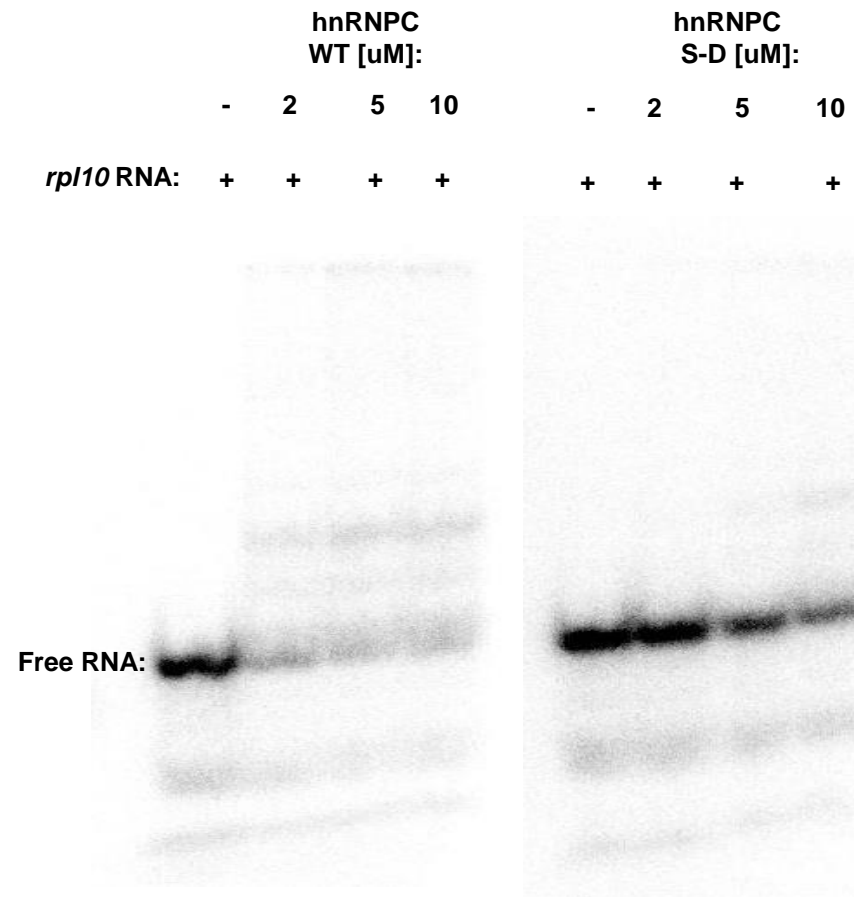

Supplement: Supplementary file 7 — Source data Fig. 3 [file 44318_2025_374_MOESM7_ESM.zip › EMBOJ-2024-118552_Source data_Fig. 3/3E/3E.pdf]

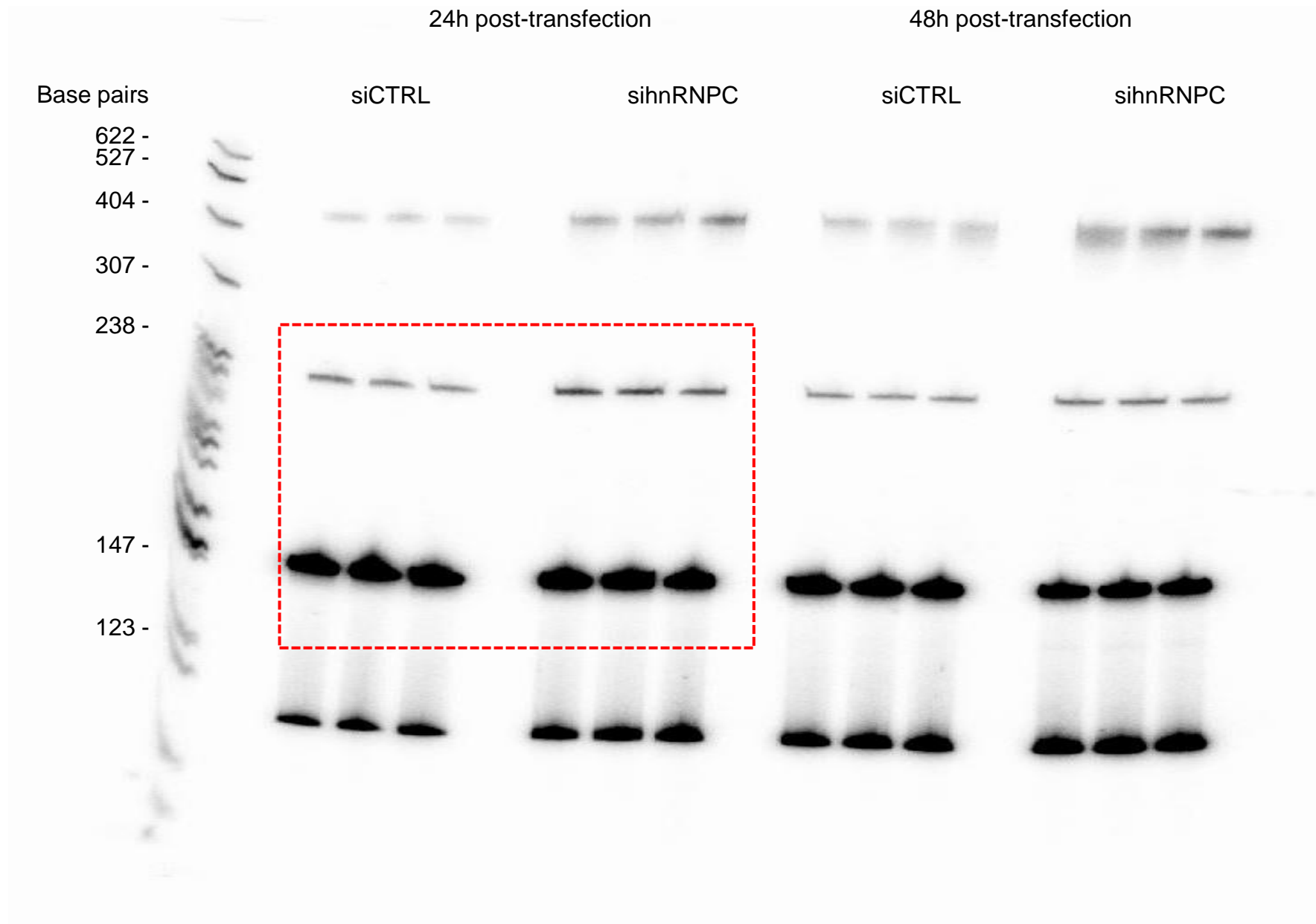

Supplement: Supplementary file 7 — Source data Fig. 3 [file 44318_2025_374_MOESM7_ESM.zip › EMBOJ-2024-118552_Source data_Fig. 3/3C/3C.pdf]

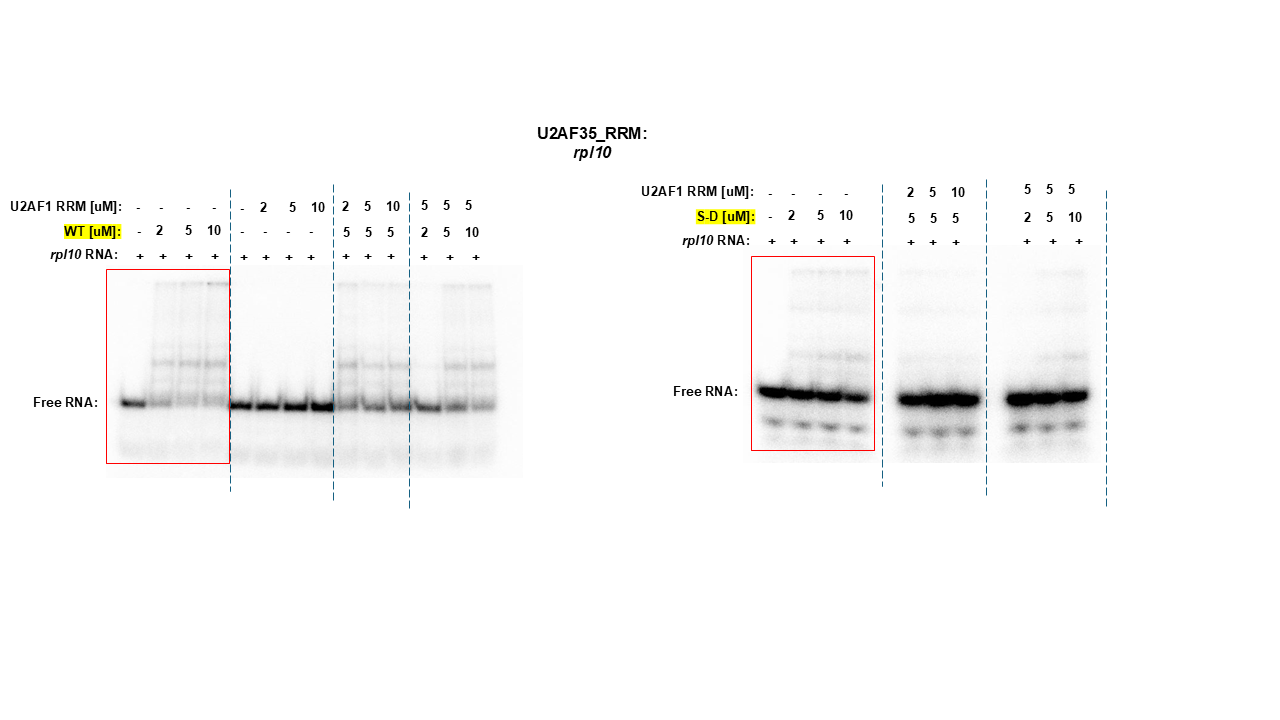

Supplement: Supplementary file 7 — Source data Fig. 3 [file 44318_2025_374_MOESM7_ESM.zip › EMBOJ-2024-118552_Source data_Fig. 3/3E/3E/Slide1.TIF]

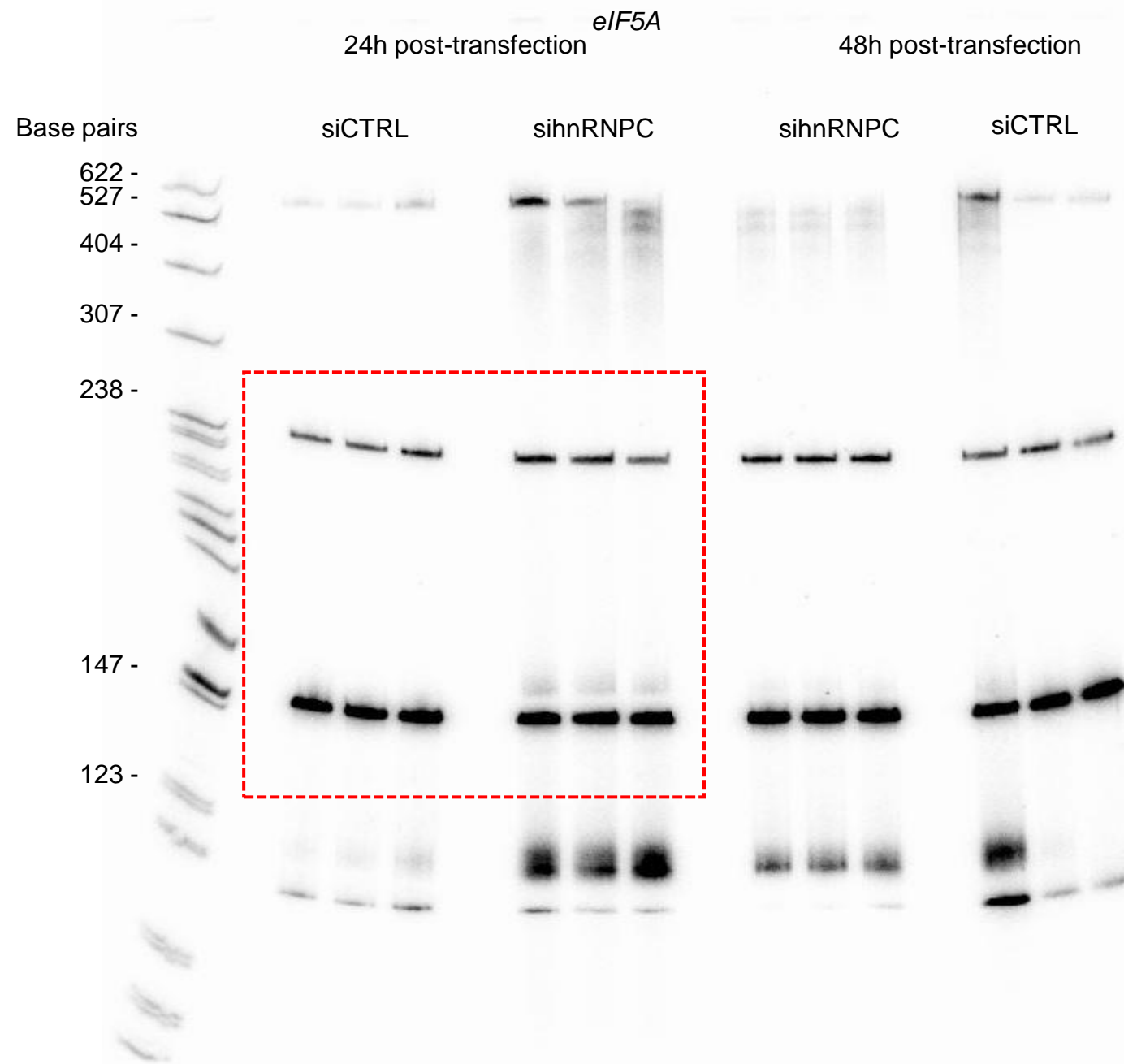

Supplement: Supplementary file 7 — Source data Fig. 3 [file 44318_2025_374_MOESM7_ESM.zip › EMBOJ-2024-118552_Source data_Fig. 3/3D/3D.pdf]

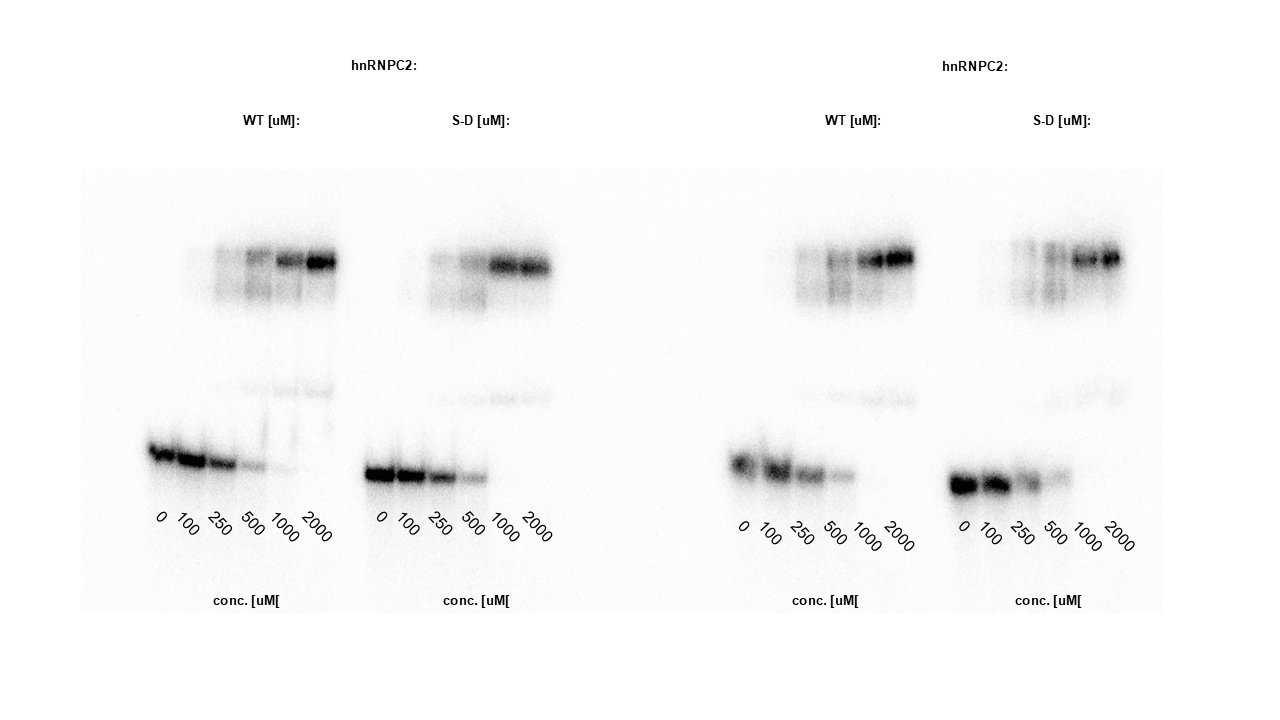

Supplement: Supplementary file 7 — Source data Fig. 3 [file 44318_2025_374_MOESM7_ESM.zip › EMBOJ-2024-118552_Source data_Fig. 3/3G/3G/Slide1.TIF]

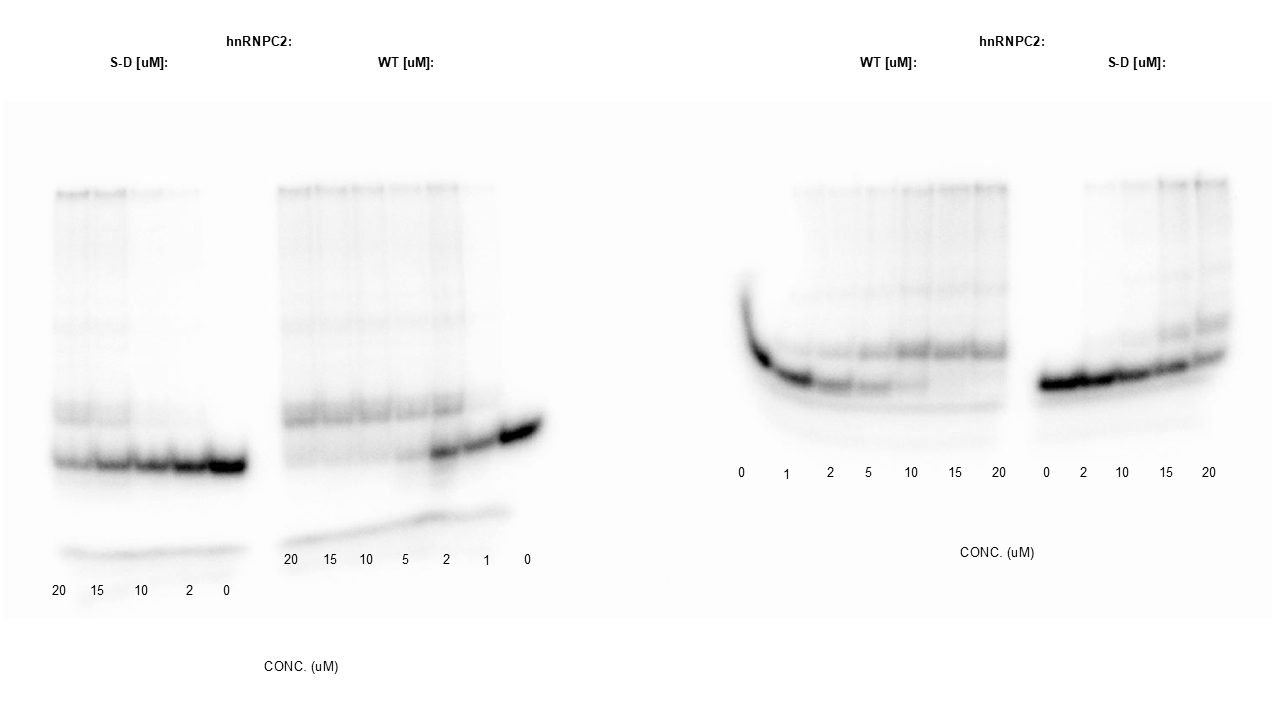

Supplement: Supplementary file 7 — Source data Fig. 3 [file 44318_2025_374_MOESM7_ESM.zip › EMBOJ-2024-118552_Source data_Fig. 3/3F/3F/Slide2.TIF]

hnRNP C2:

WT [ $\mu$ M]:

S-D [ $\mu$ M]:

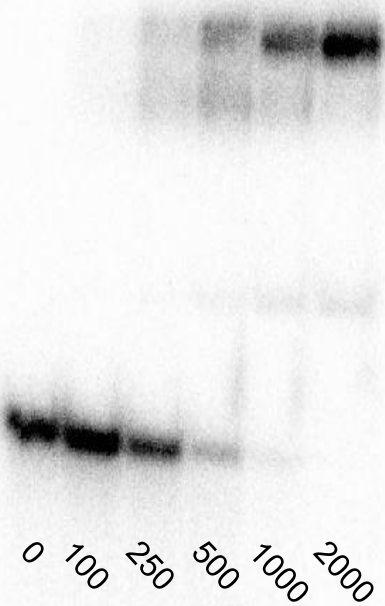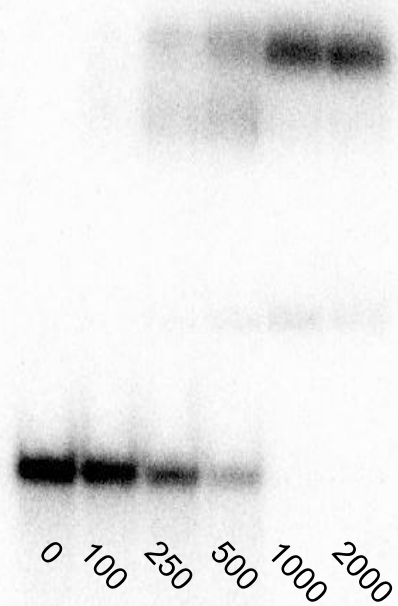

hnRNP C2:

WT [ $\mu$ M]:

S-D [ $\mu$ M]:

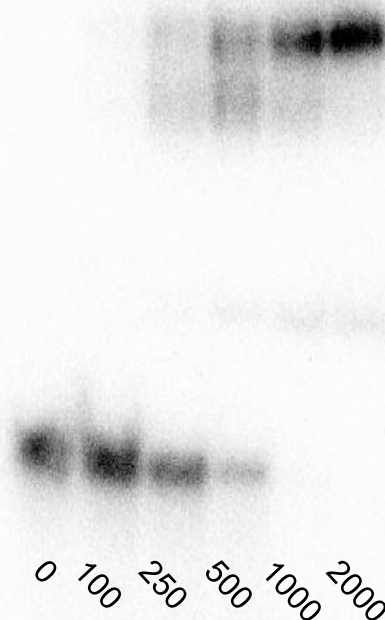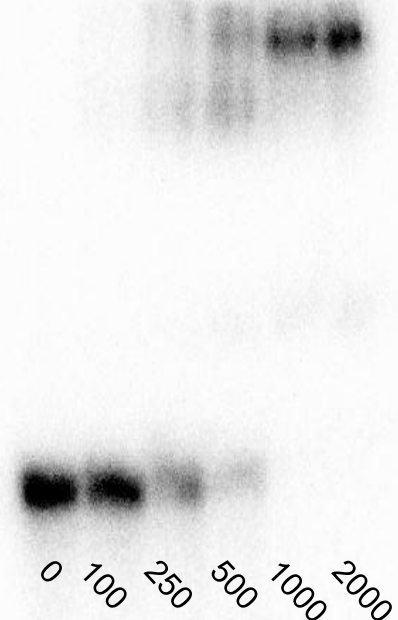

hnRNP2:

WT [uM]:

S-D [uM]:

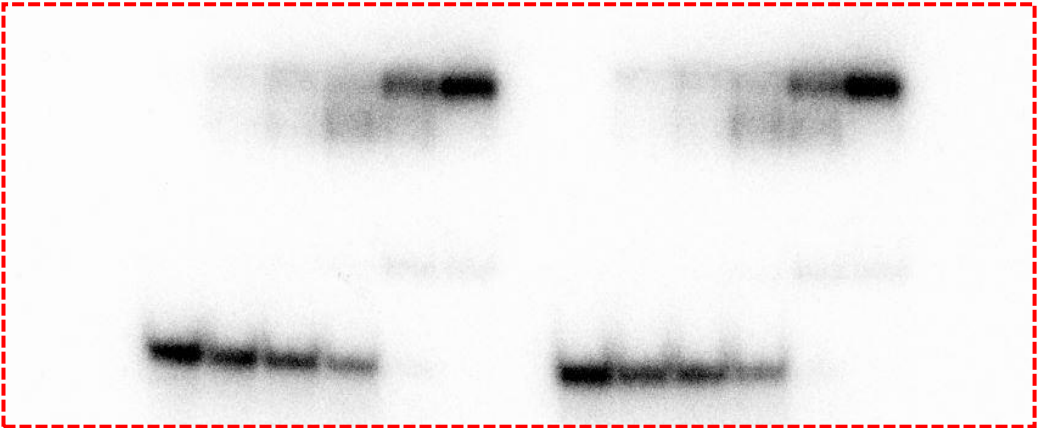

0 100 250 500 1000 2000

0 100 250 500 1000 2000

conc. [uM]

conc. [uM]

Supplement: Supplementary file 7 — Source data Fig. 3 [file 44318_2025_374_MOESM7_ESM.zip › EMBOJ-2024-118552_Source data_Fig. 3/3G/3G.pdf]

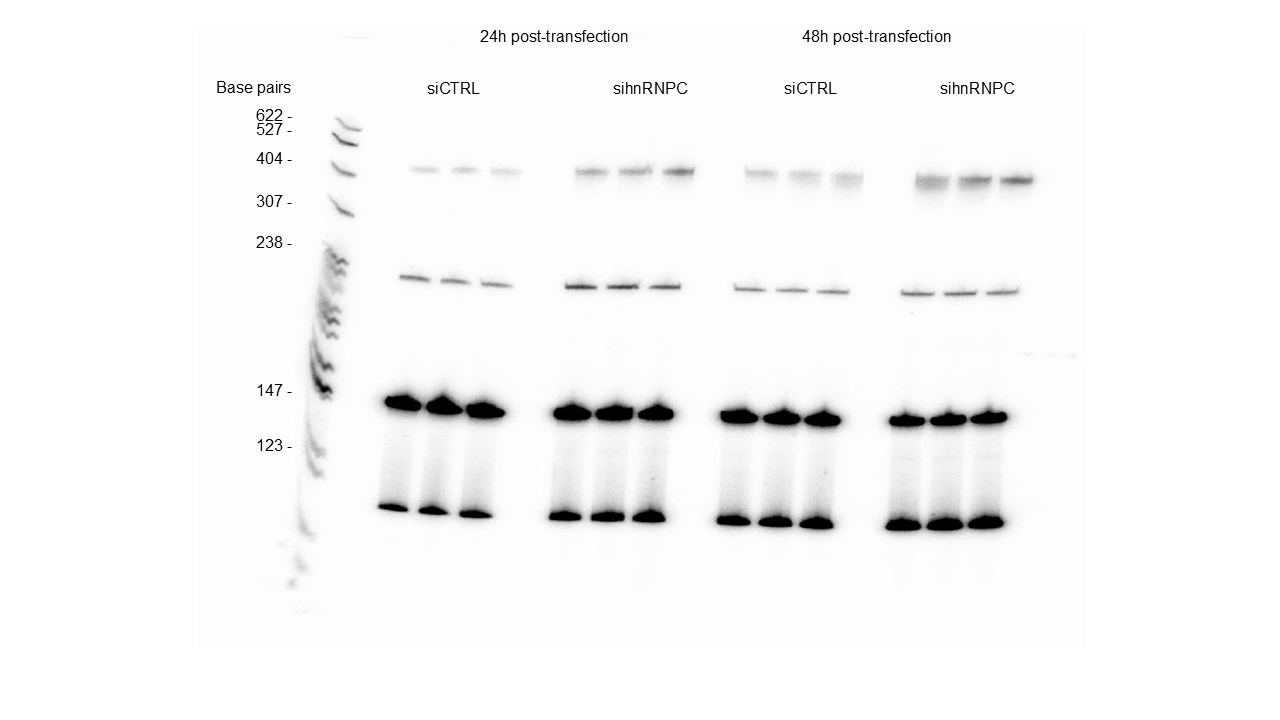

Supplement: Supplementary file 7 — Source data Fig. 3 [file 44318_2025_374_MOESM7_ESM.zip › EMBOJ-2024-118552_Source data_Fig. 3/3C/3C.tif]

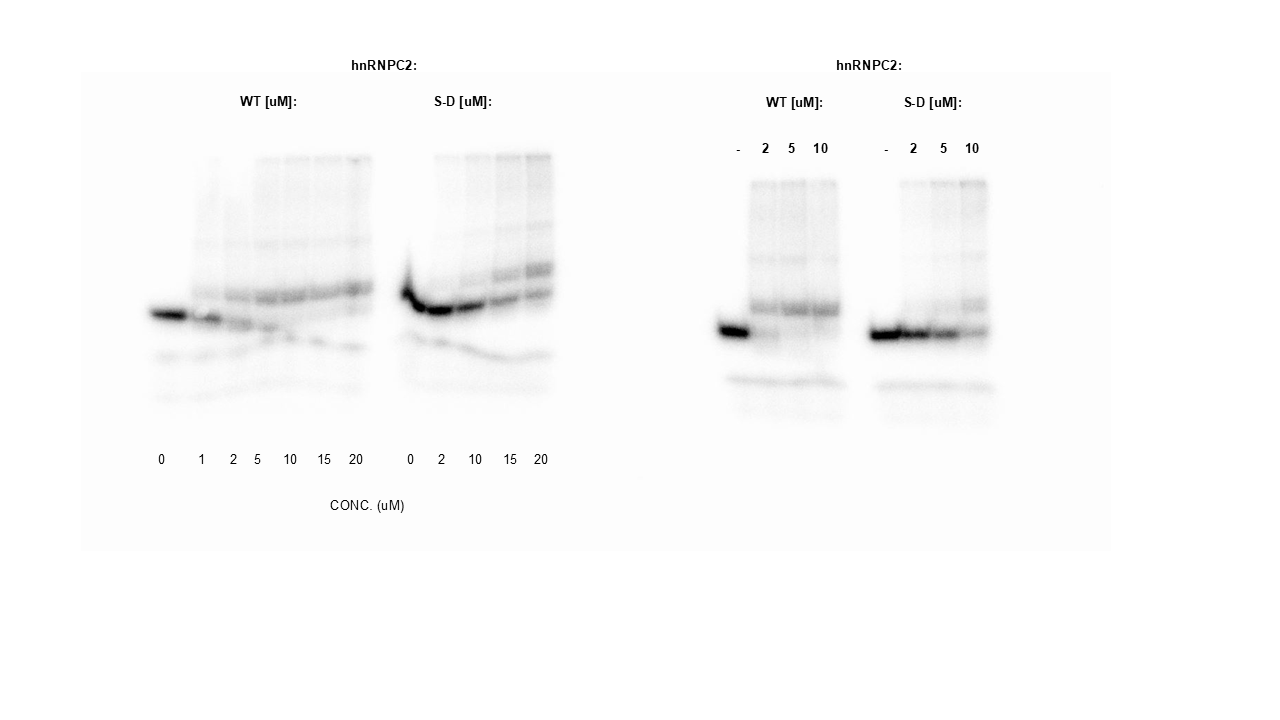

Supplement: Supplementary file 7 — Source data Fig. 3 [file 44318_2025_374_MOESM7_ESM.zip › EMBOJ-2024-118552_Source data_Fig. 3/3F/3F/Slide1.TIF]

hnRNP C2:

WT [uM]:

S-D [uM]:

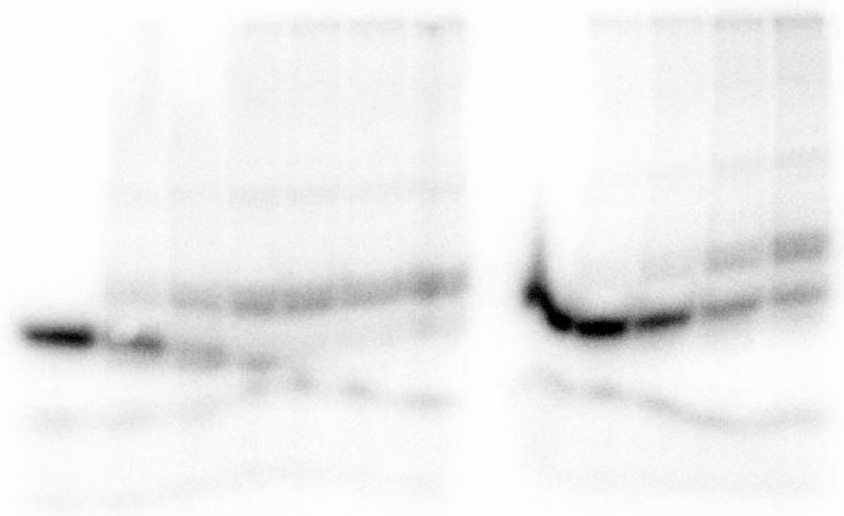

0 1 2 5 10 15 20 0 2 10 15 20

CONC. (uM)

hnRNP C2:

WT [uM]:

S-D [uM]:

- 2 5 10 - 2 5 10

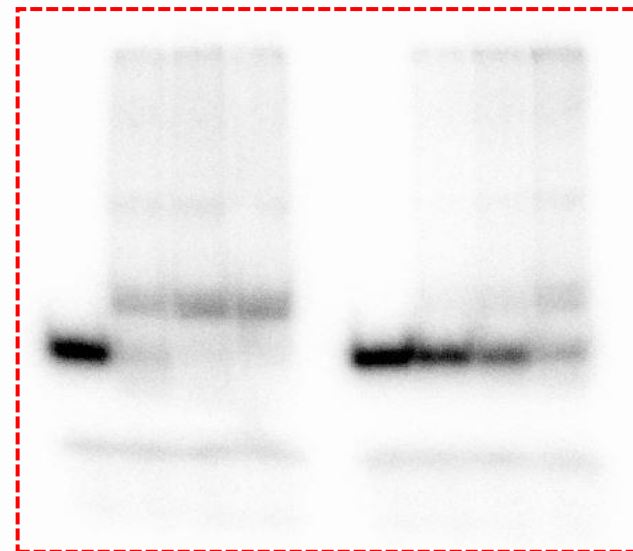

**WT [uM]:**

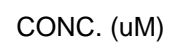

**S-D [uM]:**

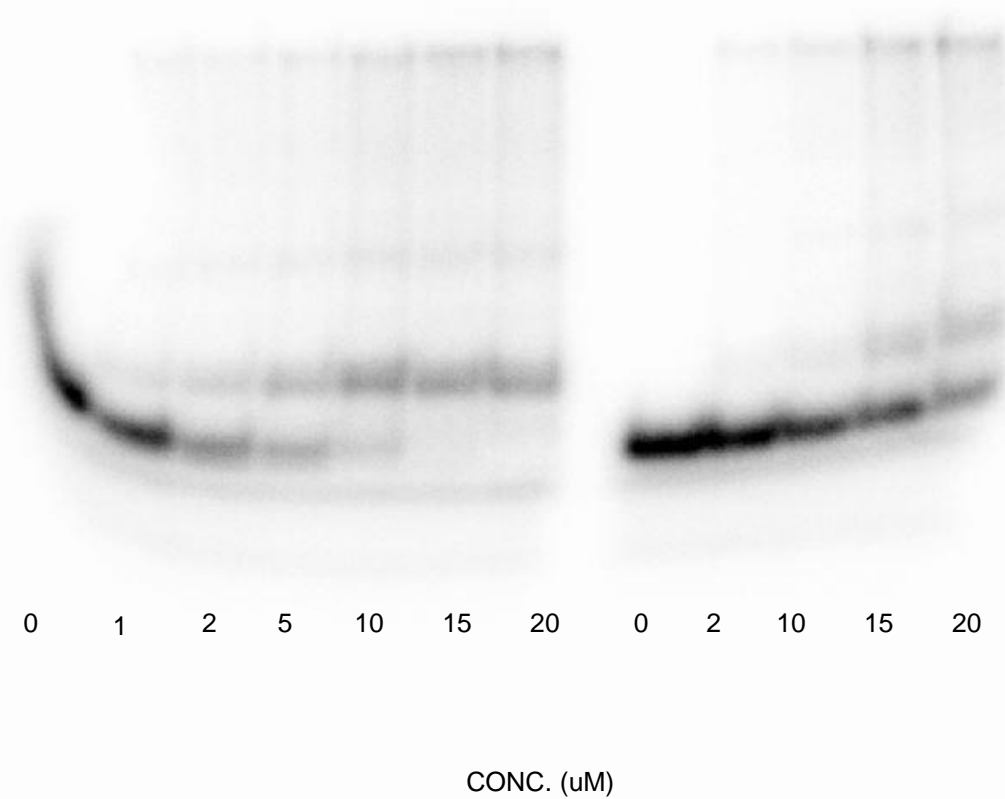

Supplement: Supplementary file 7 — Source data Fig. 3 [file 44318_2025_374_MOESM7_ESM.zip › EMBOJ-2024-118552_Source data_Fig. 3/3F/3F.pdf]

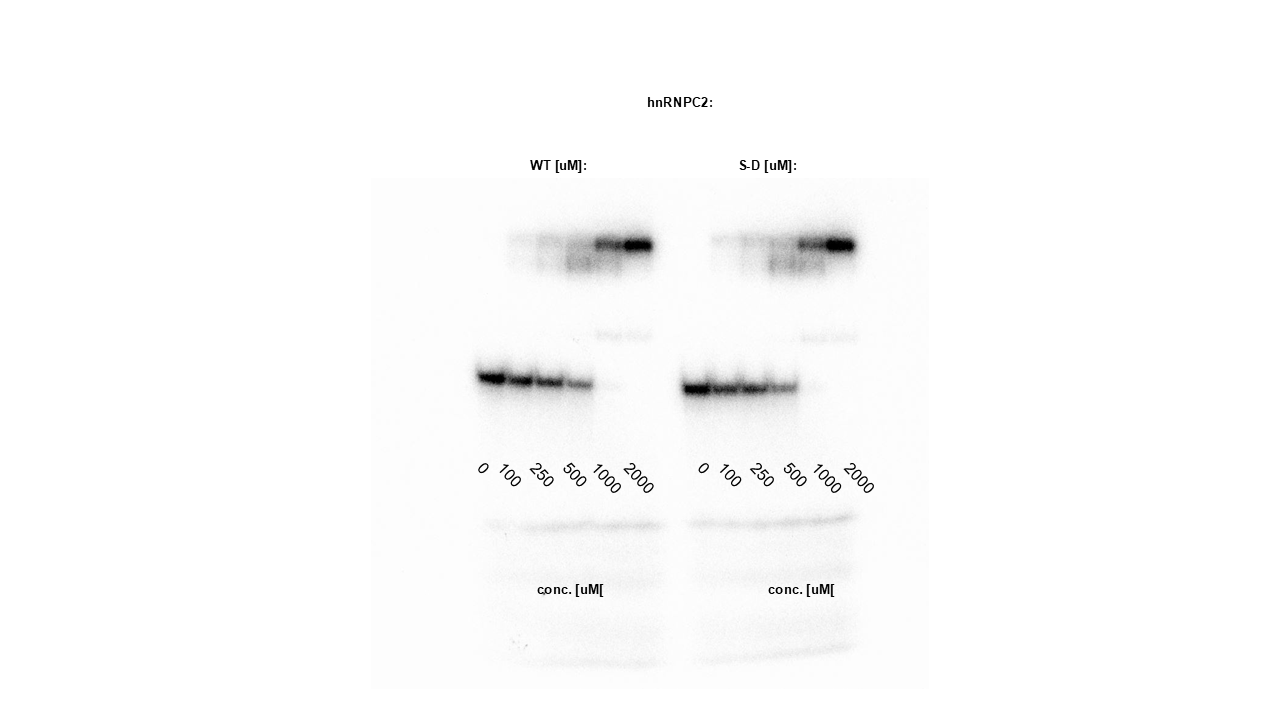

Supplement: Supplementary file 7 — Source data Fig. 3 [file 44318_2025_374_MOESM7_ESM.zip › EMBOJ-2024-118552_Source data_Fig. 3/3G/3G/Slide2.TIF]

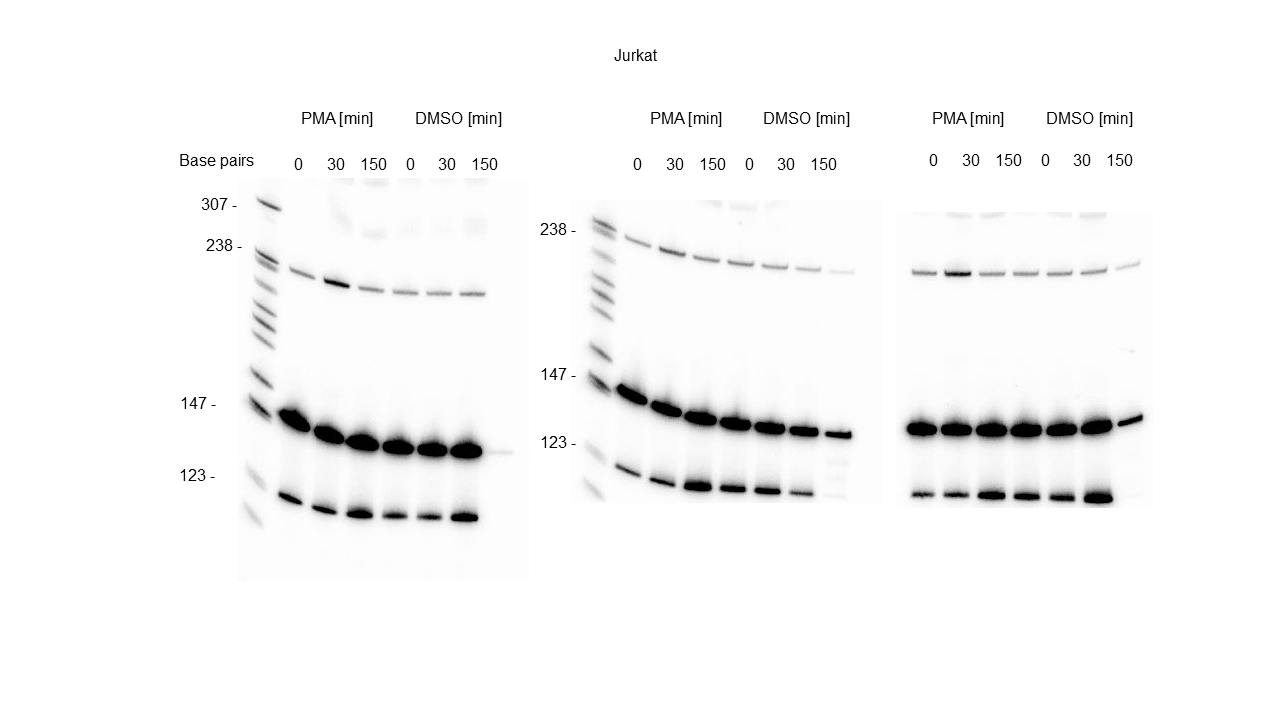

Supplement: Supplementary file 8 — Source data Fig. 4 [file 44318_2025_374_MOESM8_ESM.zip › EMBOJ-2024-118552_Source data_Fig. 4/4E/4E/Slide4.TIF]

Base pairs

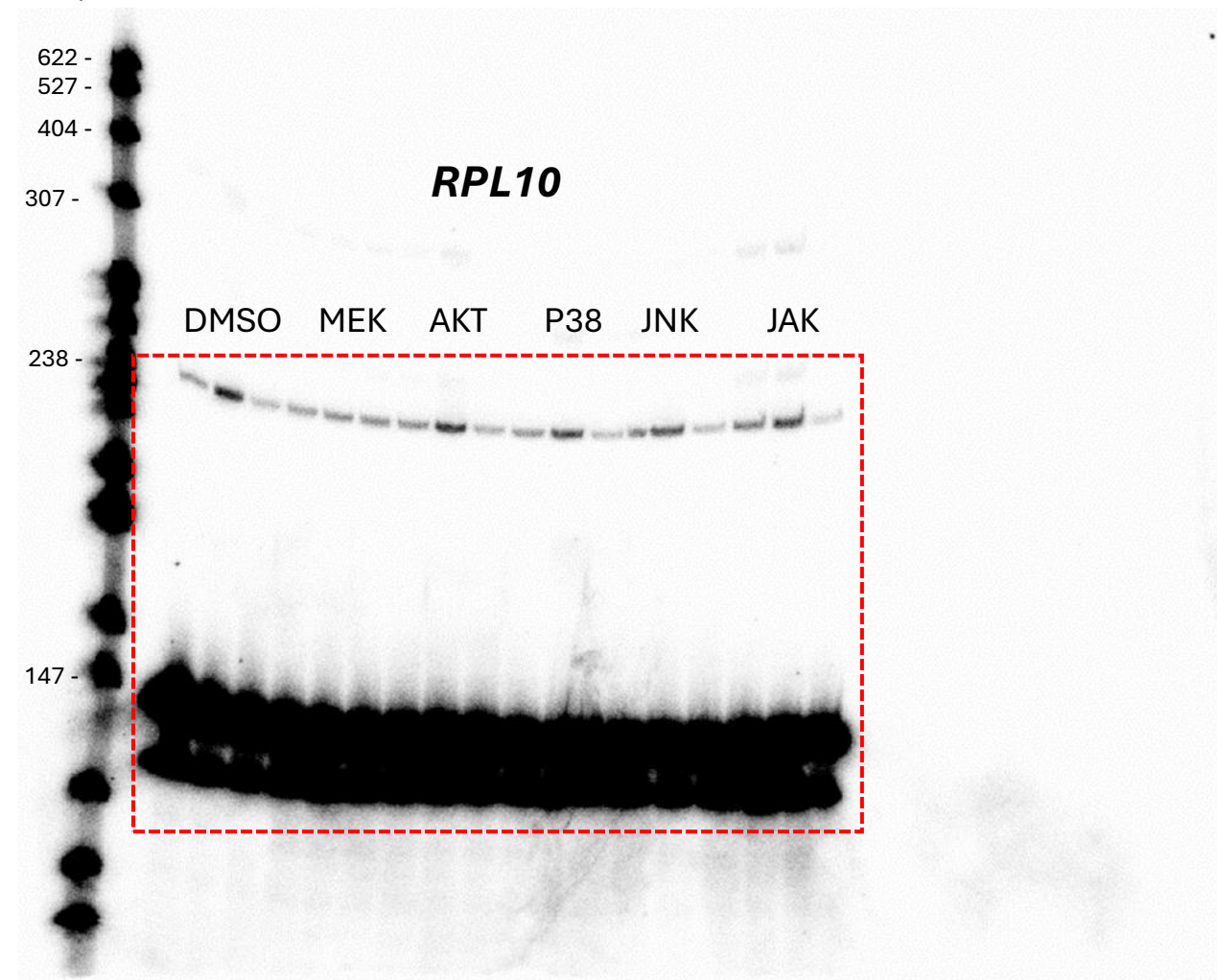

Supplement: Supplementary file 8 — Source data Fig. 4 [file 44318_2025_374_MOESM8_ESM.zip › EMBOJ-2024-118552_Source data_Fig. 4/4A/4A.pdf]

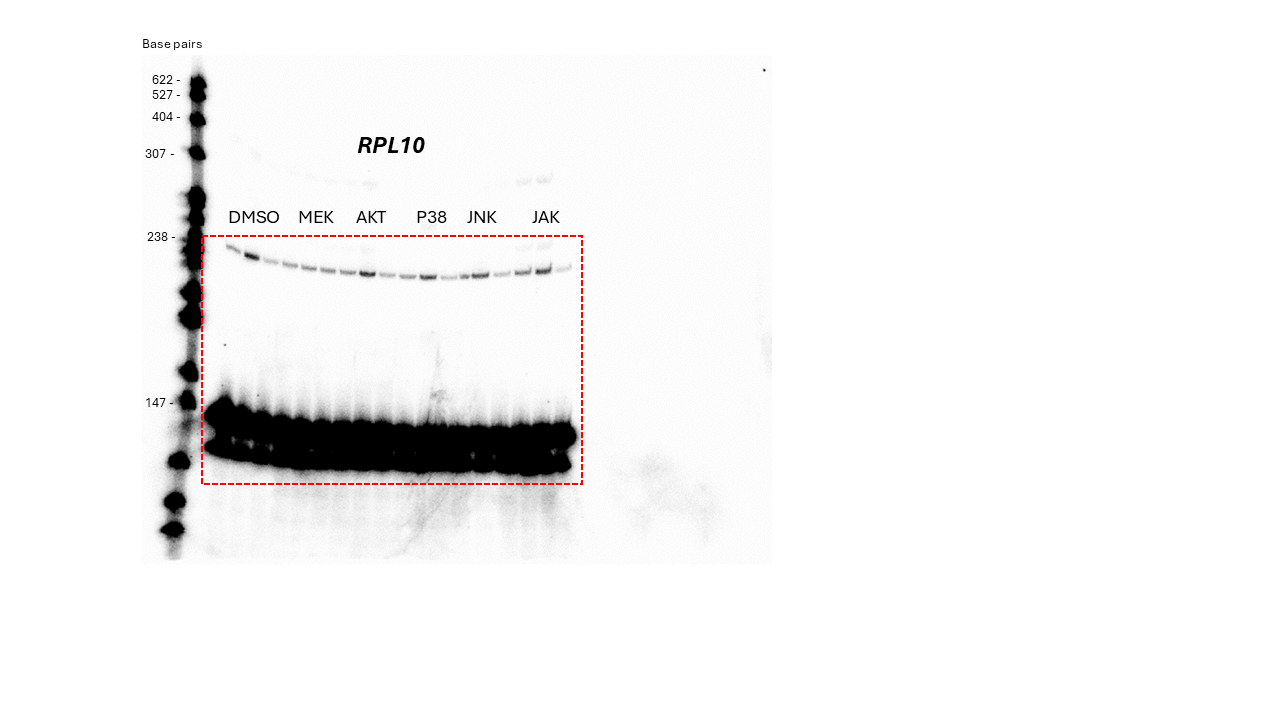

Supplement: Supplementary file 8 — Source data Fig. 4 [file 44318_2025_374_MOESM8_ESM.zip › EMBOJ-2024-118552_Source data_Fig. 4/4A/4A.tif]

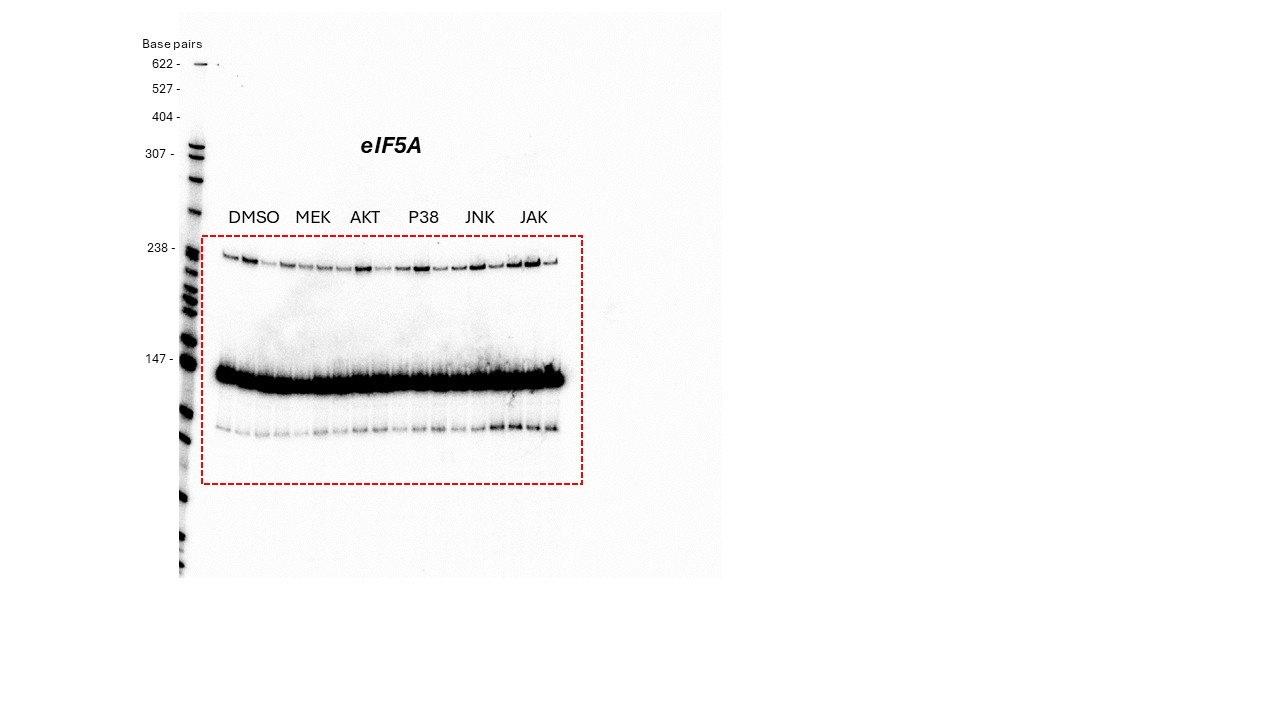

Supplement: Supplementary file 8 — Source data Fig. 4 [file 44318_2025_374_MOESM8_ESM.zip › EMBOJ-2024-118552_Source data_Fig. 4/4B/4B.tif]

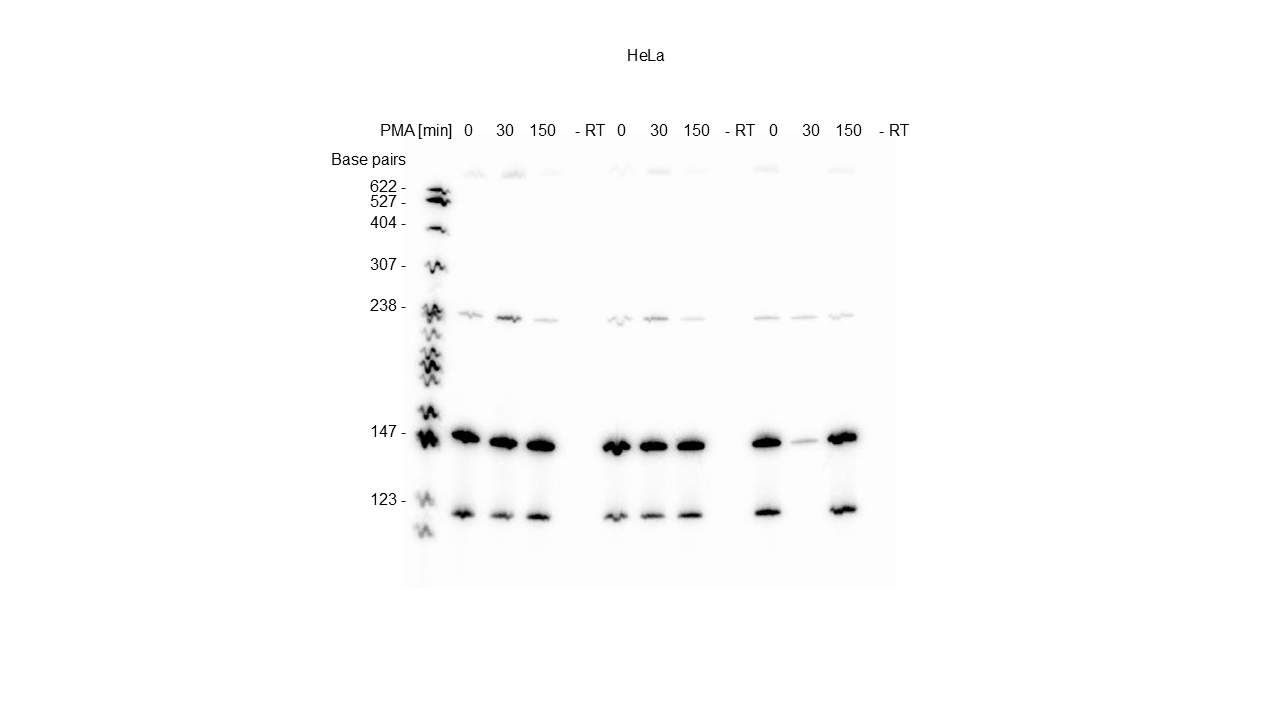

Supplement: Supplementary file 8 — Source data Fig. 4 [file 44318_2025_374_MOESM8_ESM.zip › EMBOJ-2024-118552_Source data_Fig. 4/4E/4E/Slide2.TIF]

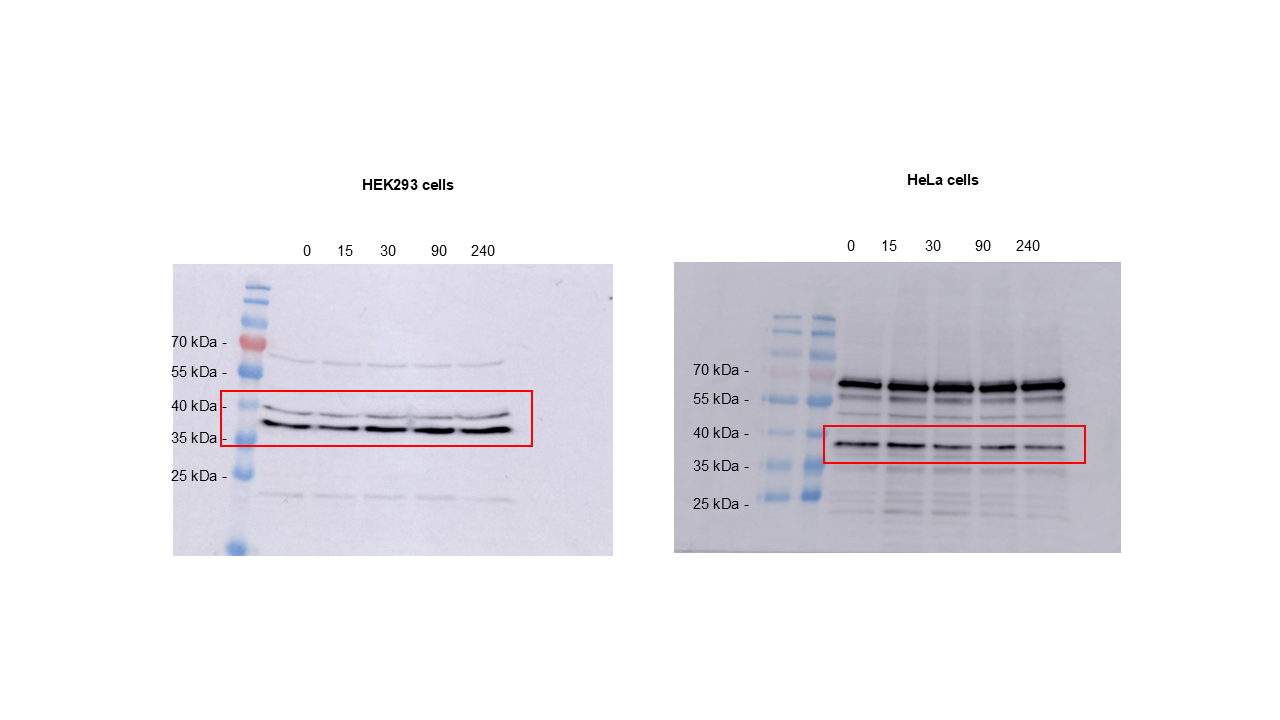

Supplement: Supplementary file 8 — Source data Fig. 4 [file 44318_2025_374_MOESM8_ESM.zip › EMBOJ-2024-118552_Source data_Fig. 4/4D/4D.tif]

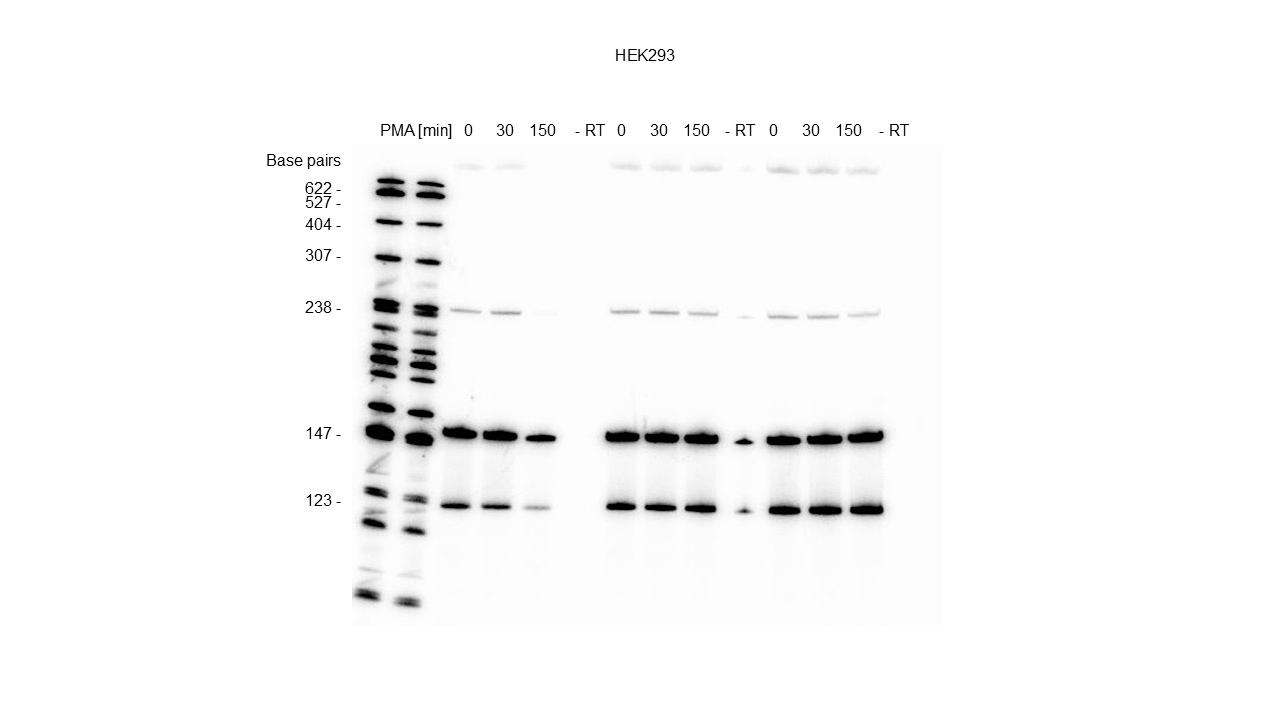

Supplement: Supplementary file 8 — Source data Fig. 4 [file 44318_2025_374_MOESM8_ESM.zip › EMBOJ-2024-118552_Source data_Fig. 4/4E/4E/Slide1.TIF]

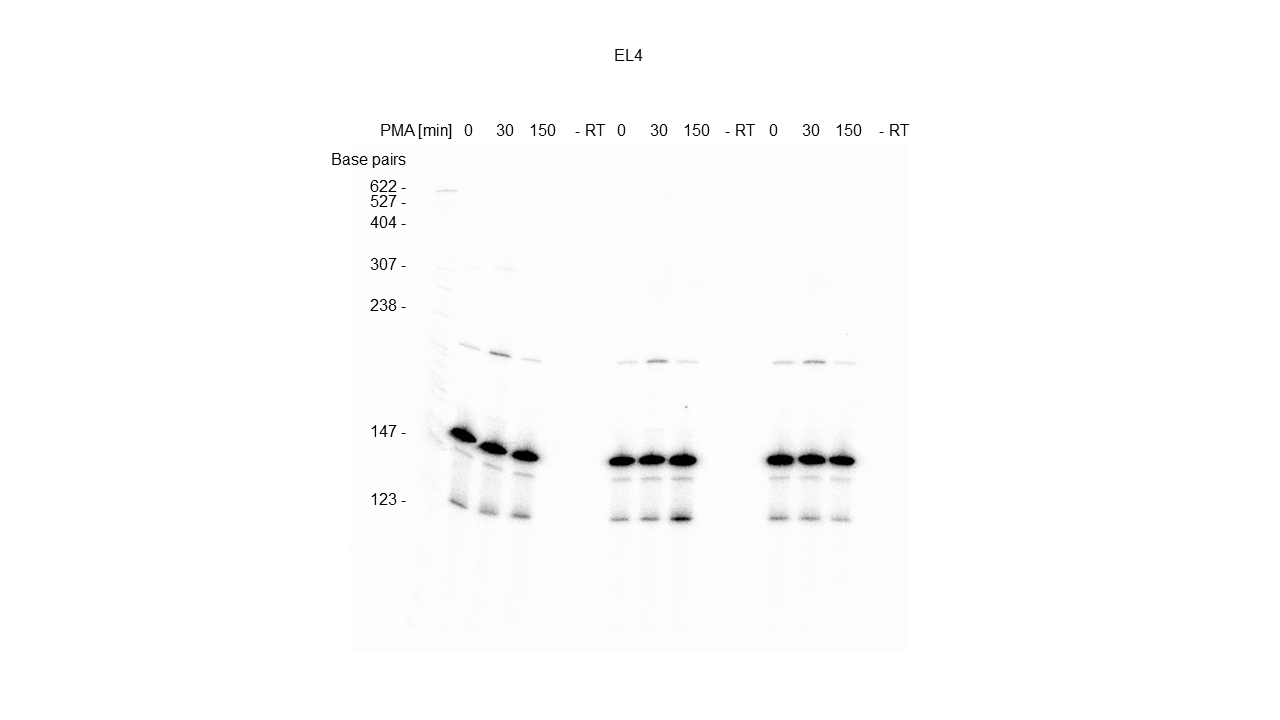

Supplement: Supplementary file 8 — Source data Fig. 4 [file 44318_2025_374_MOESM8_ESM.zip › EMBOJ-2024-118552_Source data_Fig. 4/4E/4E/Slide3.TIF]

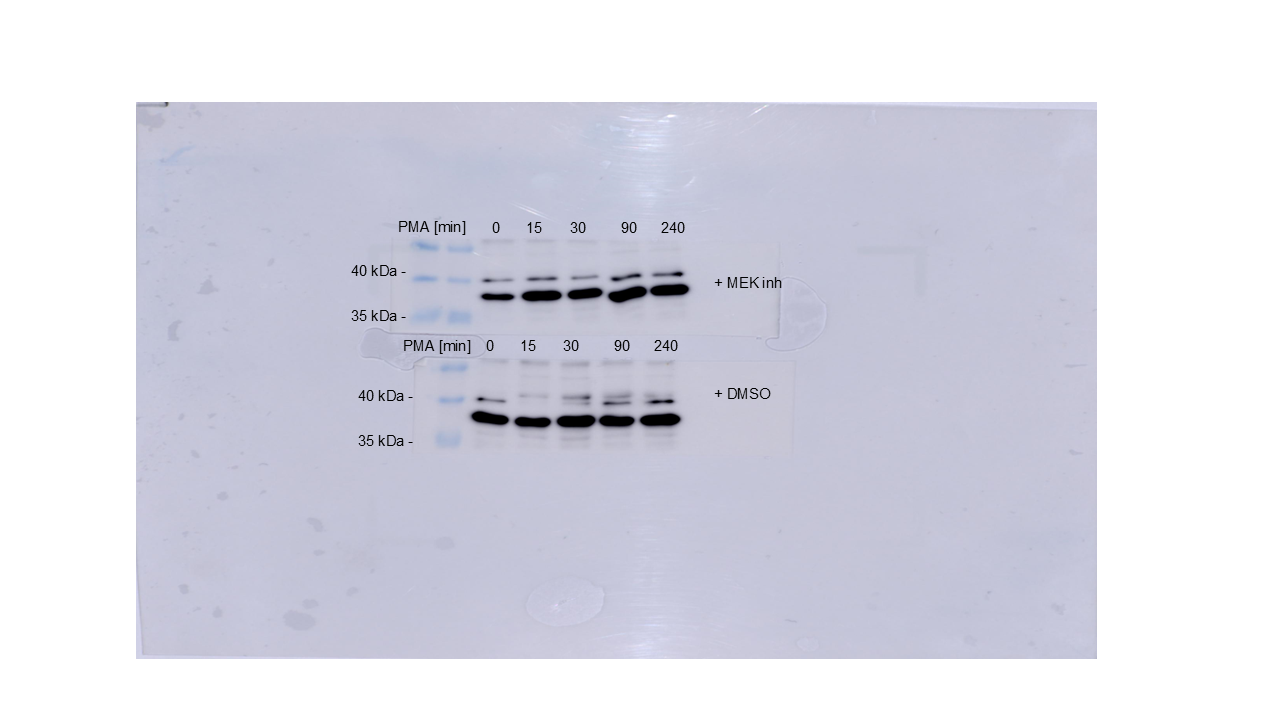

Supplement: Supplementary file 8 — Source data Fig. 4 [file 44318_2025_374_MOESM8_ESM.zip › EMBOJ-2024-118552_Source data_Fig. 4/4C/4C.tif]

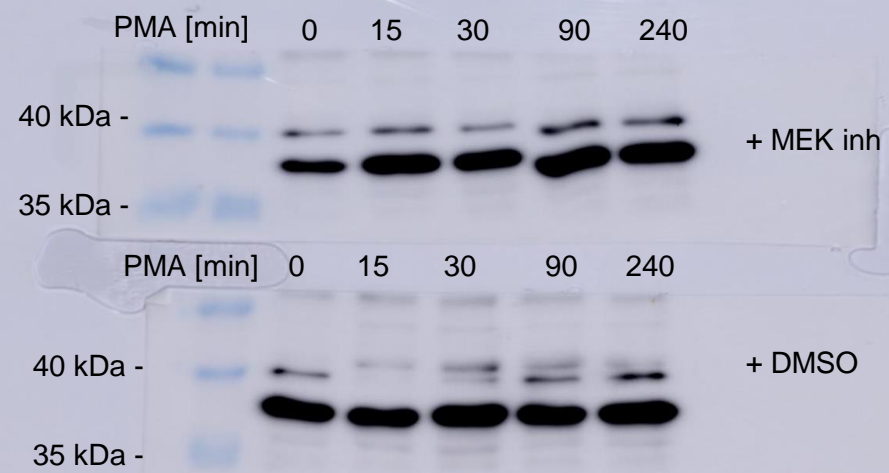

Supplement: Supplementary file 8 — Source data Fig. 4 [file 44318_2025_374_MOESM8_ESM.zip › EMBOJ-2024-118552_Source data_Fig. 4/4C/4C.pdf]

Base pairs

622 -

527 -

404 -

307 -

***eIF5A***

DMSO

MEK

AKT

P38

JNK

JAK

238 -

147 -

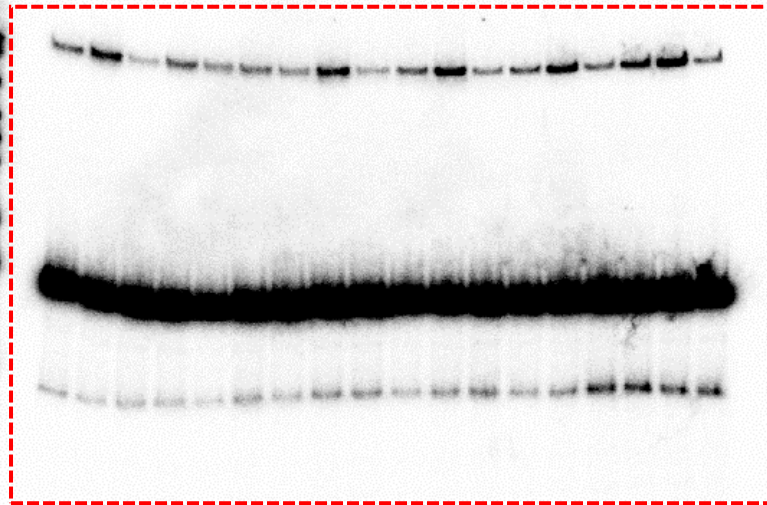

Supplement: Supplementary file 8 — Source data Fig. 4 [file 44318_2025_374_MOESM8_ESM.zip › EMBOJ-2024-118552_Source data_Fig. 4/4B/4B.pdf]

## HEK293

Base pairs

622 -

527 -

404 -

307 -

238 -

147 -

123 -

PMA [min] 0 30 150 - RT 0 30 150 - RT 0 30 150 - RT

HeLa

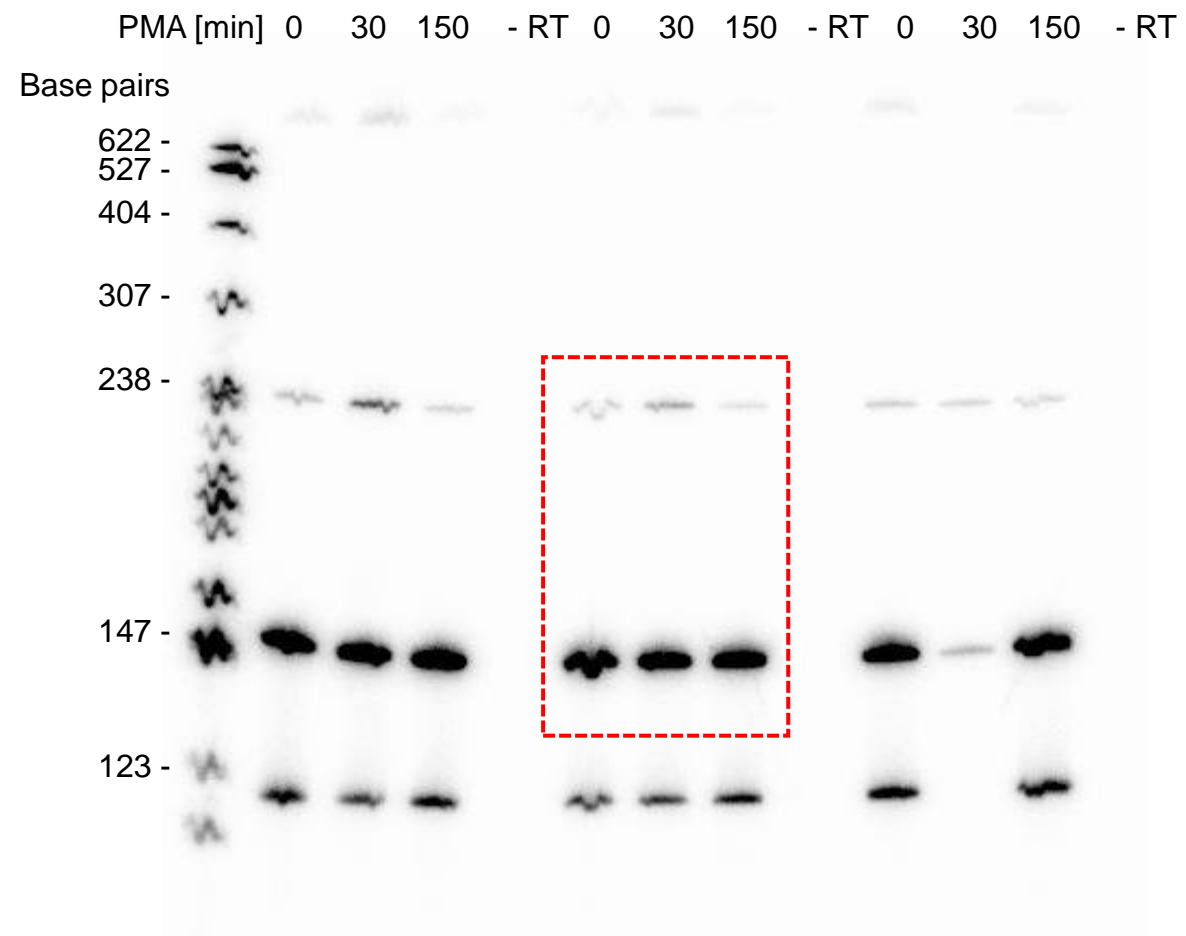

# EL4

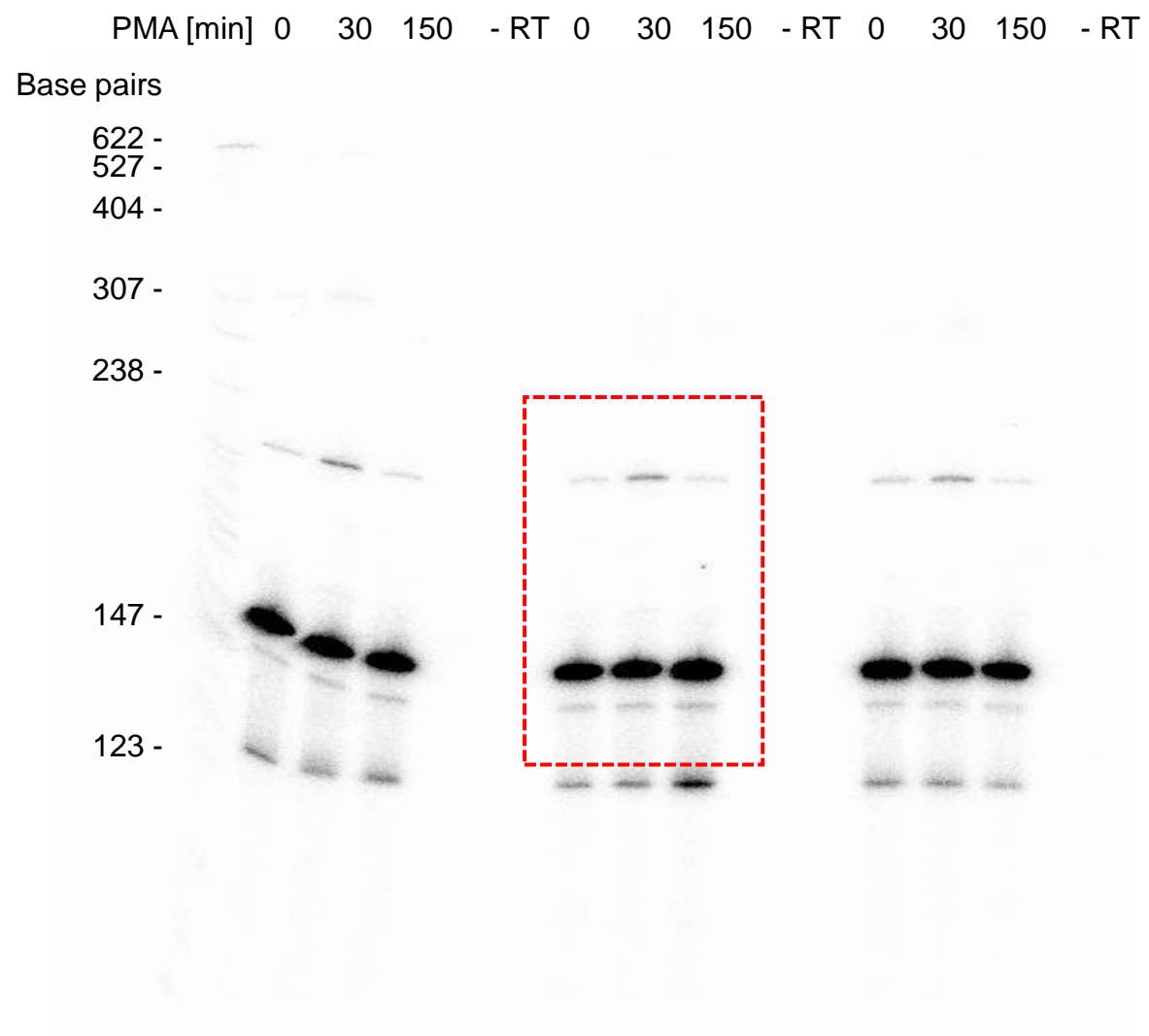

# Jurkat

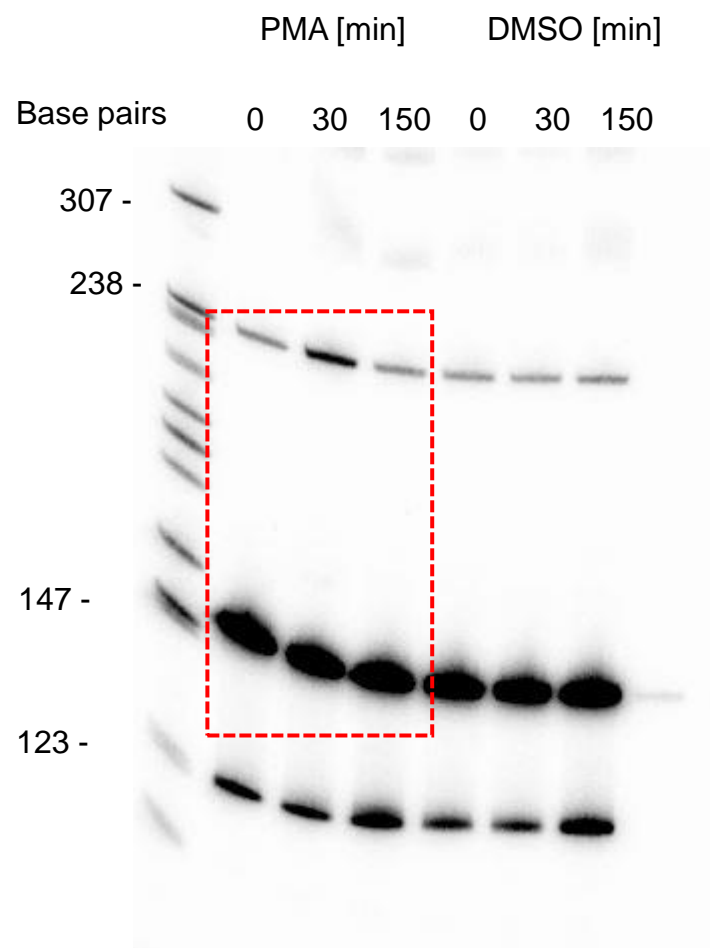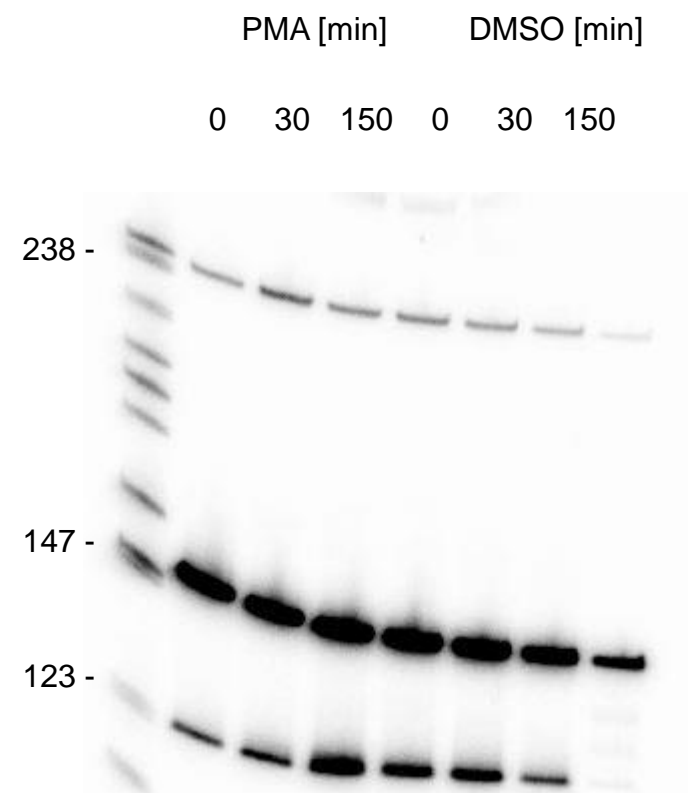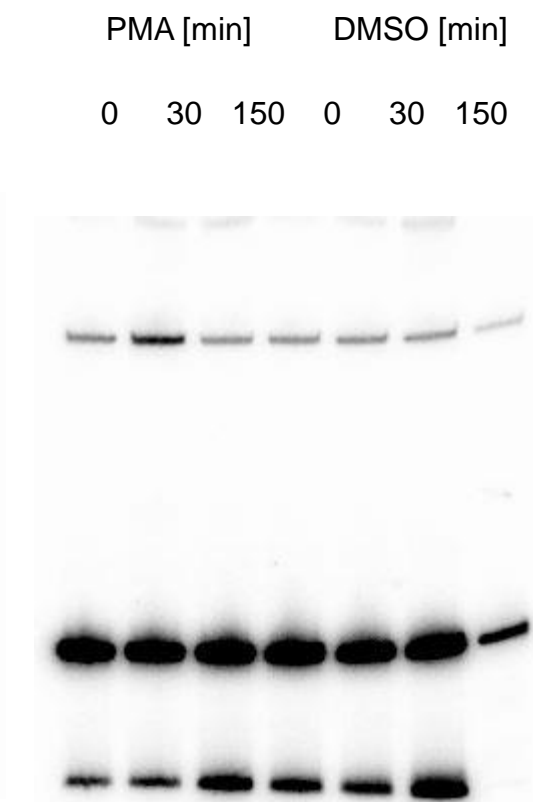

Supplement: Supplementary file 8 — Source data Fig. 4 [file 44318_2025_374_MOESM8_ESM.zip › EMBOJ-2024-118552_Source data_Fig. 4/4E/4E.pdf]

### HEK293 cells

PMA [min]

0 15 30 90 240

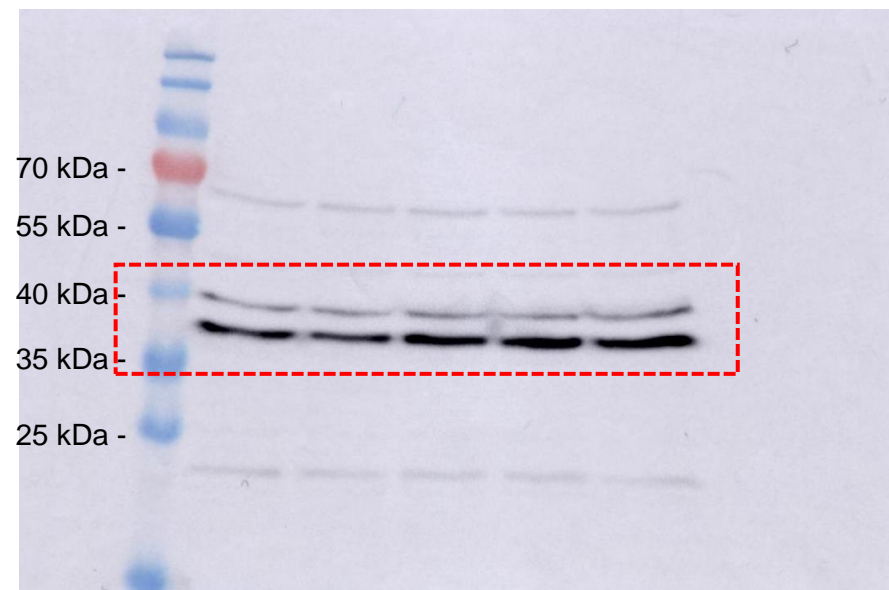

### HeLa cells

PMA [min]

0 15 30 90 240

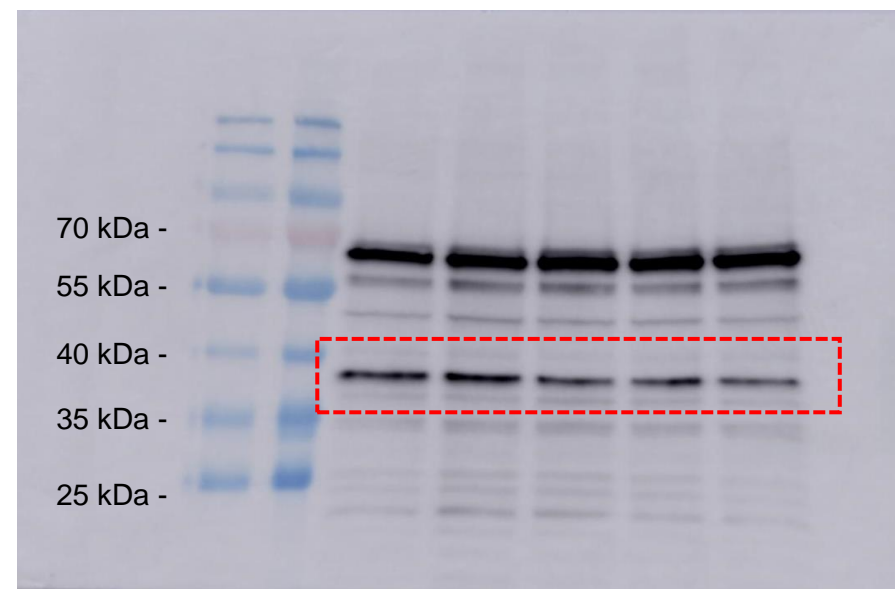

Supplement: Supplementary file 8 — Source data Fig. 4 [file 44318_2025_374_MOESM8_ESM.zip › EMBOJ-2024-118552_Source data_Fig. 4/4D/4D.pdf]

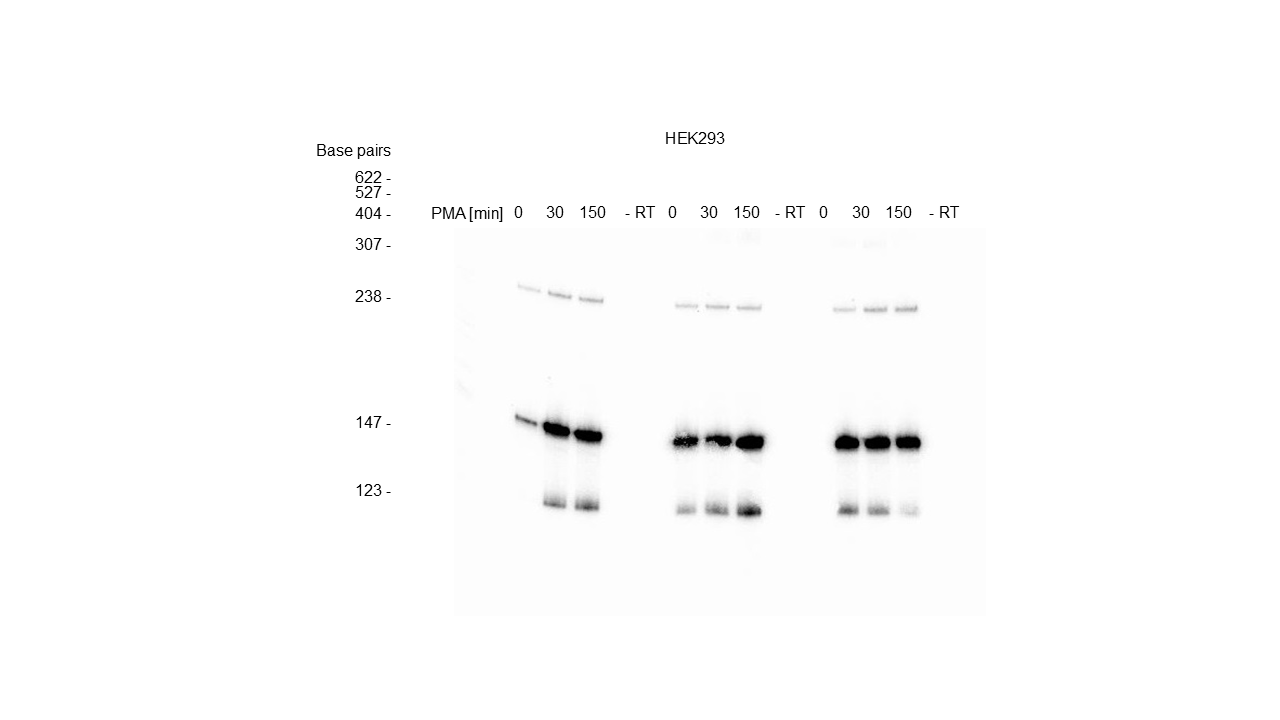

Supplement: Supplementary file 8 — Source data Fig. 4 [file 44318_2025_374_MOESM8_ESM.zip › EMBOJ-2024-118552_Source data_Fig. 4/4F/4F/Slide1.TIF]

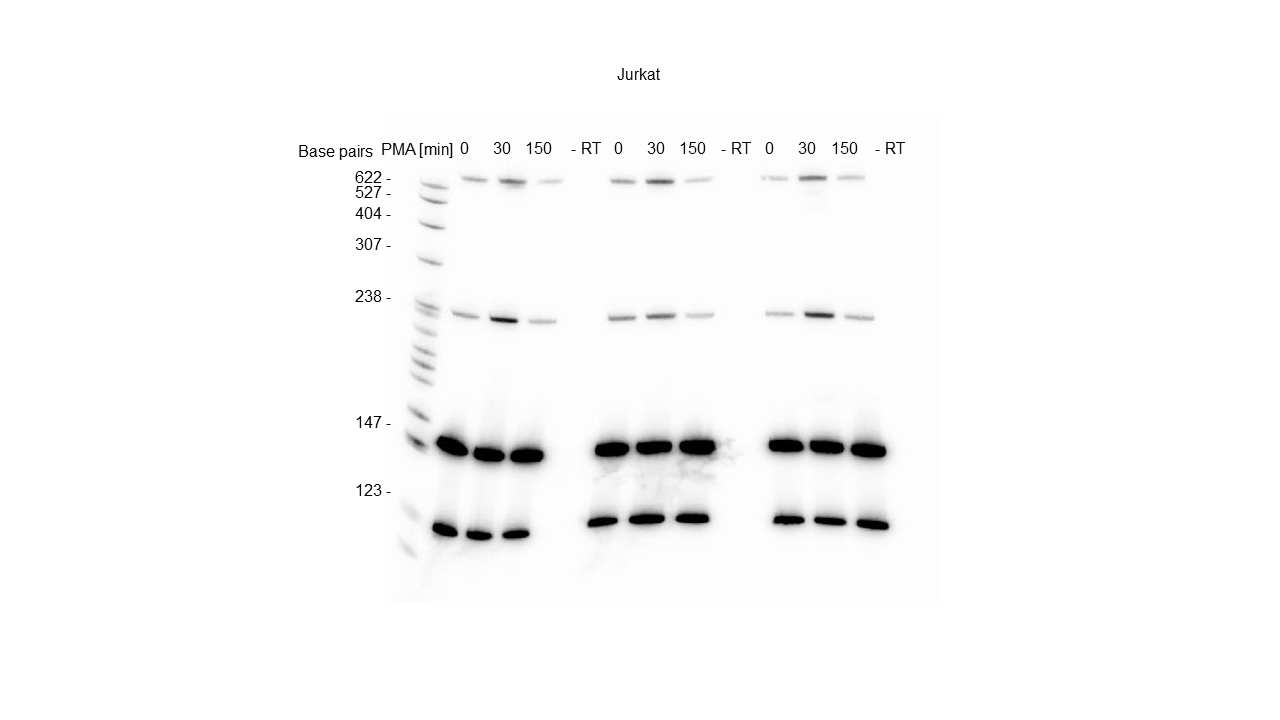

Supplement: Supplementary file 8 — Source data Fig. 4 [file 44318_2025_374_MOESM8_ESM.zip › EMBOJ-2024-118552_Source data_Fig. 4/4F/4F/Slide4.TIF]

Base pairs

HEK293

622 -

527 -

404 -

307 -

238 -

147 -

123 -

PMA [min] 0 30 150 - RT 0 30 150 - RT 0 30 150 - RT

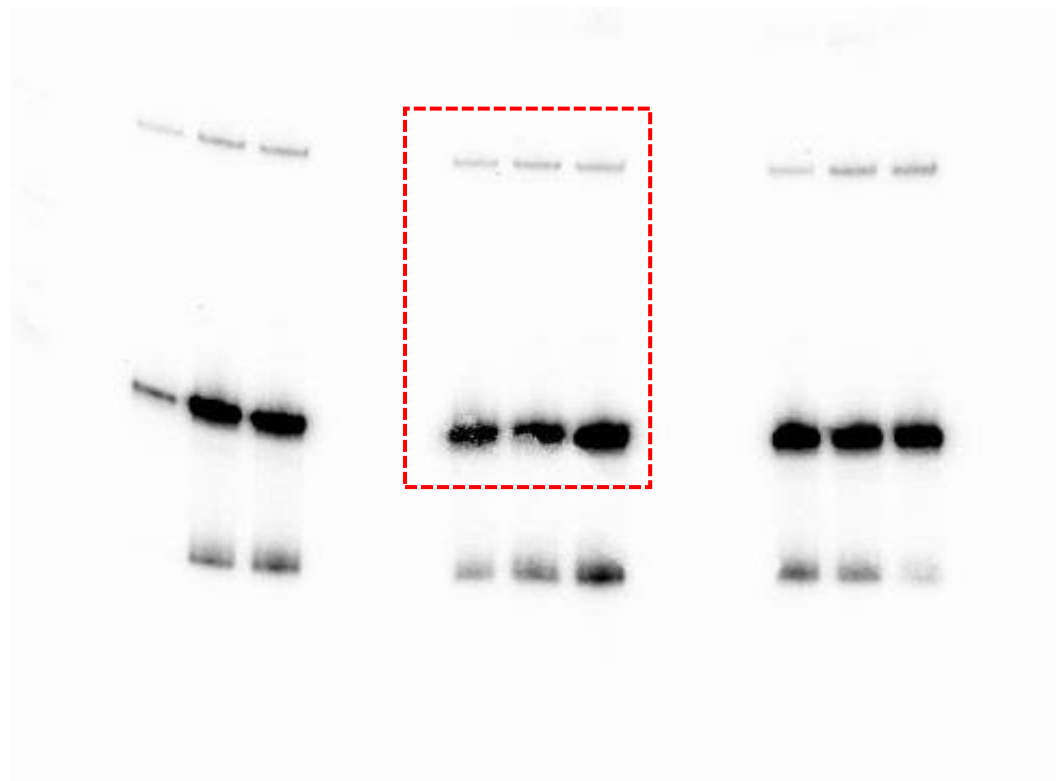

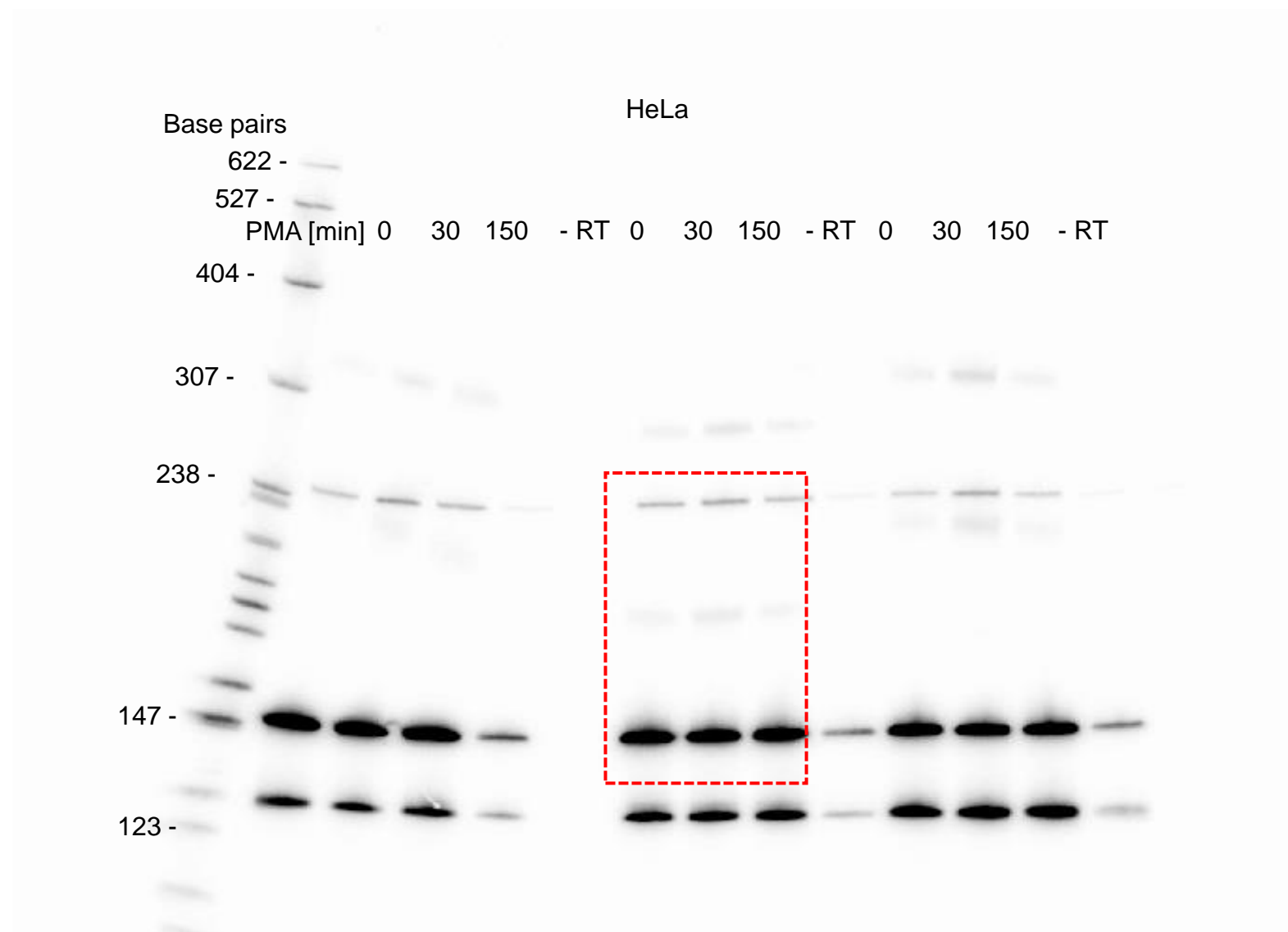

Base pairs

622 -

527 -

404 -

307 -

238 -

147 -

123 -

EL4

PMA [min] 0 30 150 - RT 0 30 150 - RT 0 30 150 - RT

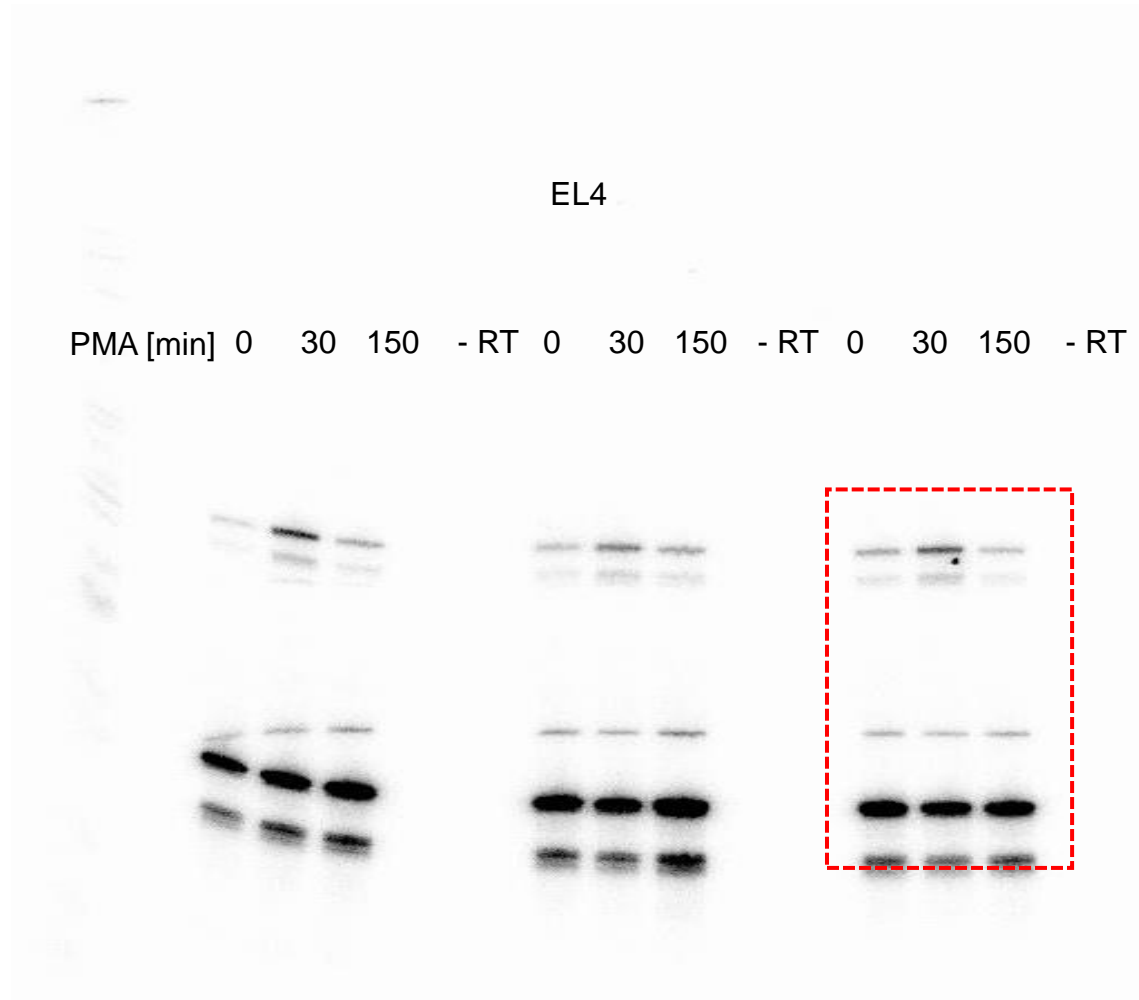

# Jurkat

Base pairs PMA [min] 0 30 150 - RT 0 30 150 - RT 0 30 150 - RT

622 -

527 -

404 -

307 -

238 -

147 -

123 -

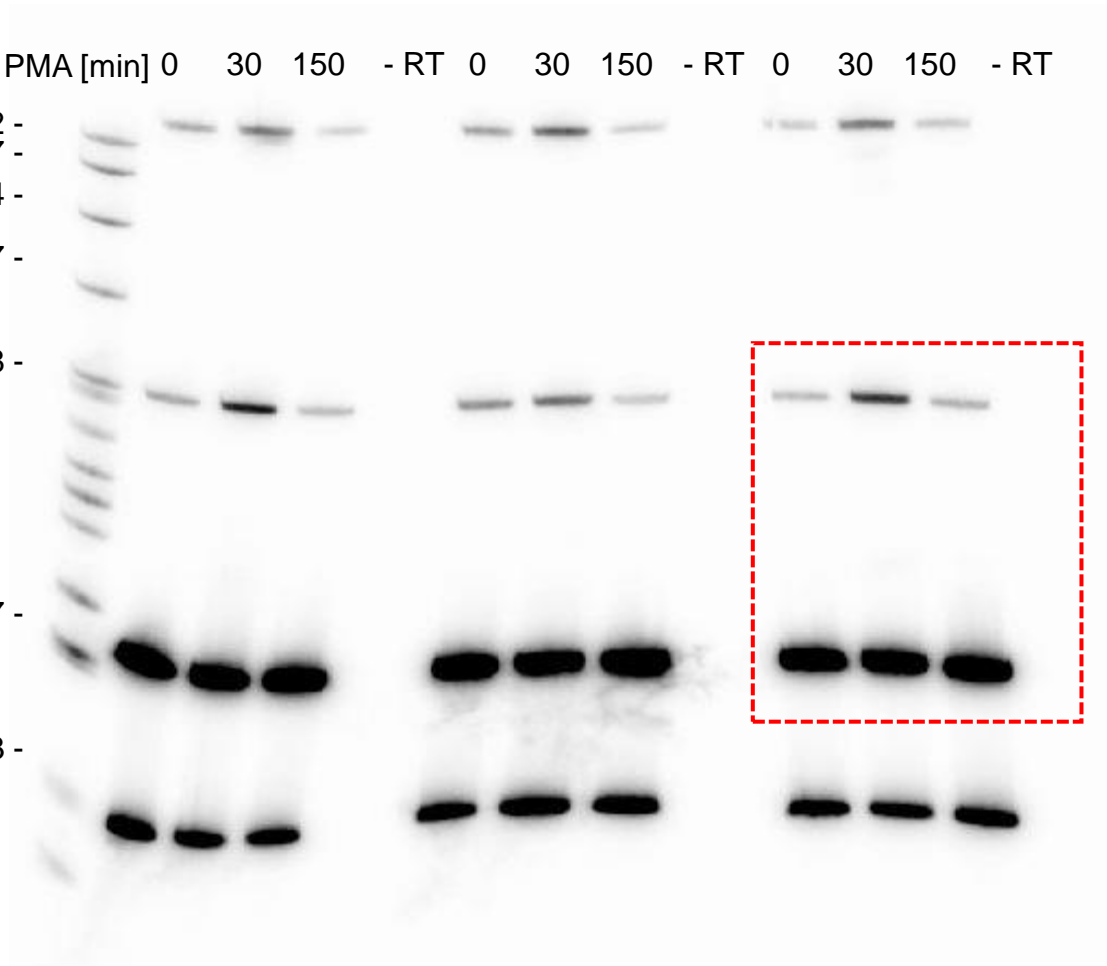

Supplement: Supplementary file 8 — Source data Fig. 4 [file 44318_2025_374_MOESM8_ESM.zip › EMBOJ-2024-118552_Source data_Fig. 4/4F/4F.pdf]

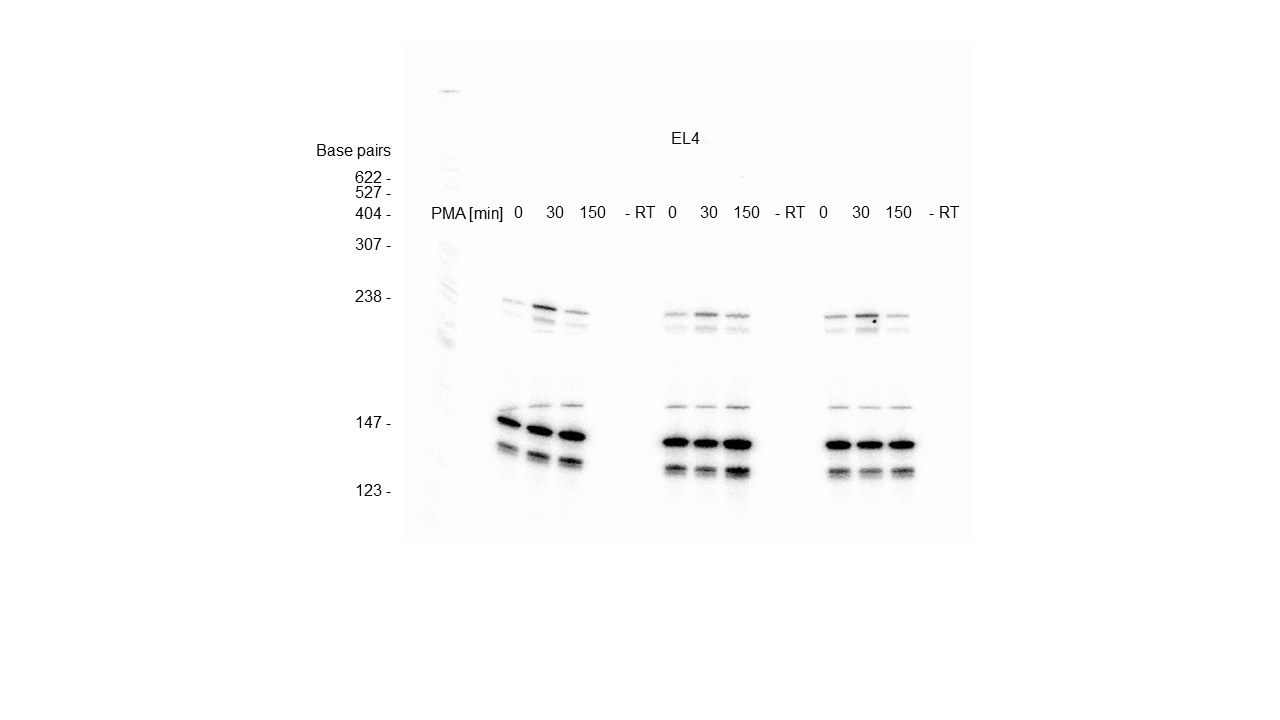

Supplement: Supplementary file 8 — Source data Fig. 4 [file 44318_2025_374_MOESM8_ESM.zip › EMBOJ-2024-118552_Source data_Fig. 4/4F/4F/Slide3.TIF]

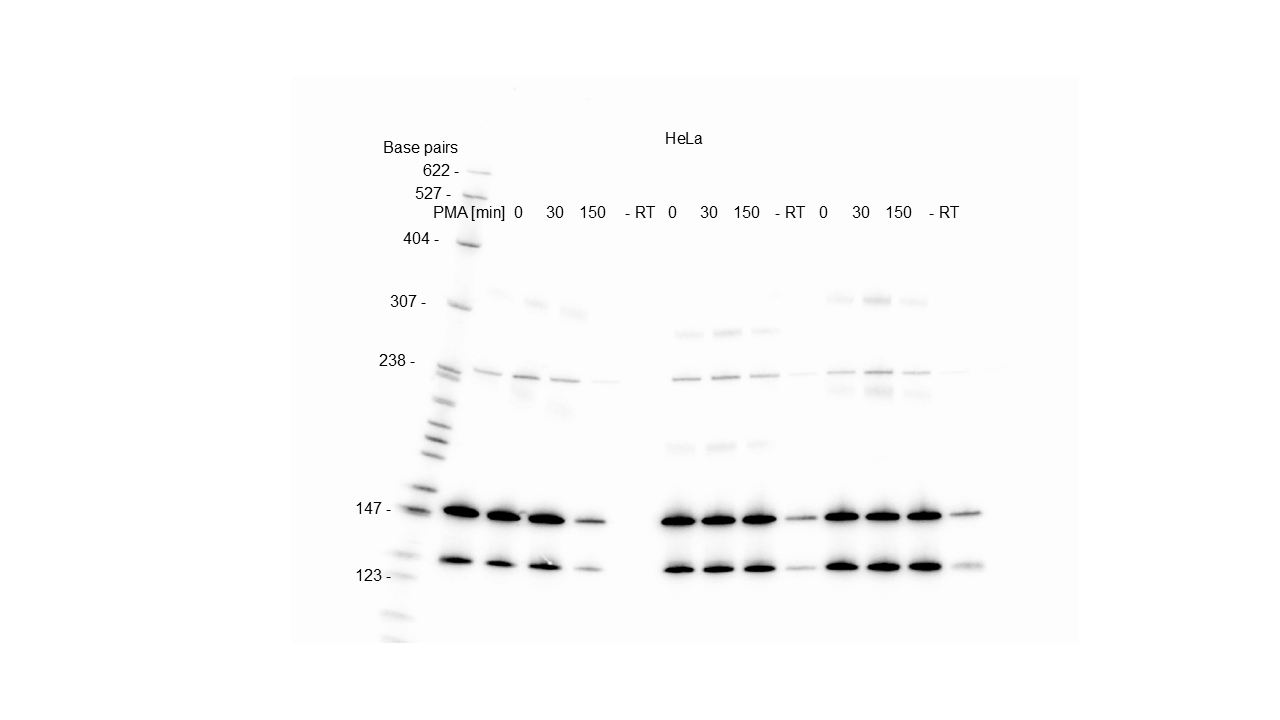

Supplement: Supplementary file 8 — Source data Fig. 4 [file 44318_2025_374_MOESM8_ESM.zip › EMBOJ-2024-118552_Source data_Fig. 4/4F/4F/Slide2.TIF]

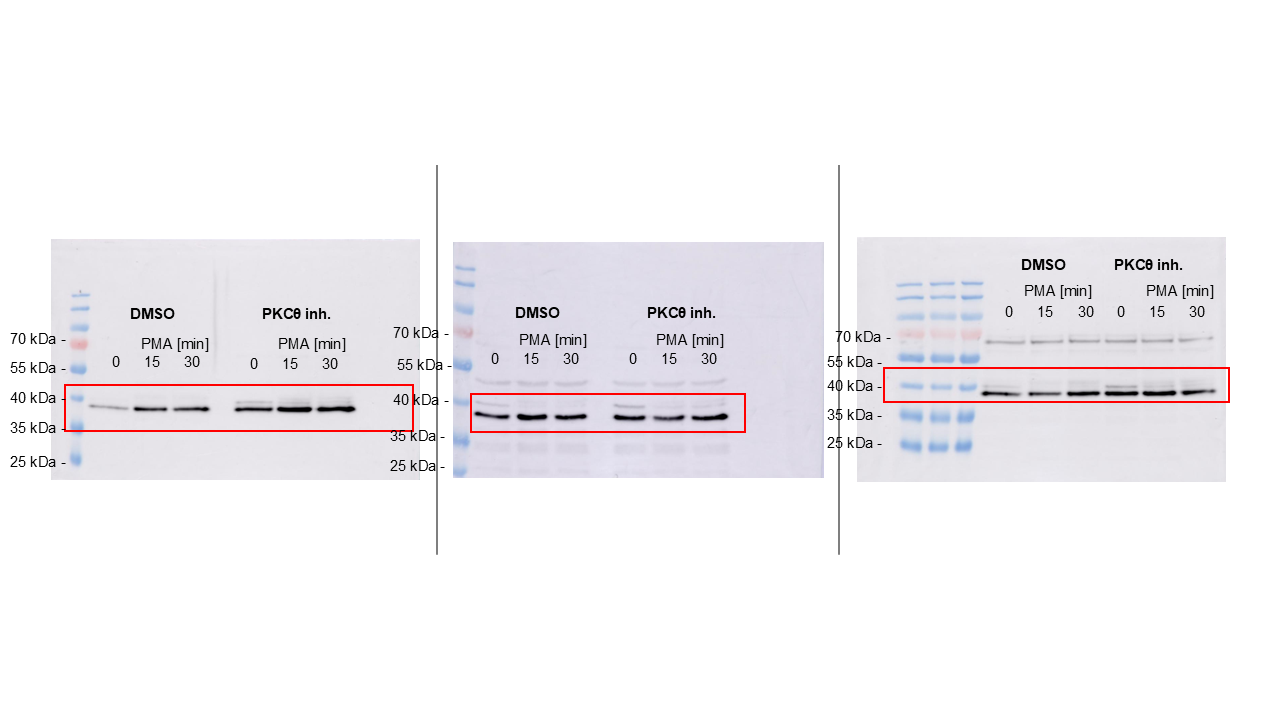

Supplement: Supplementary file 9 — Source data Fig. 5 [file 44318_2025_374_MOESM9_ESM.zip › EMBOJ-2024-118552_Source data_Fig. 5/5C/5C.tif]

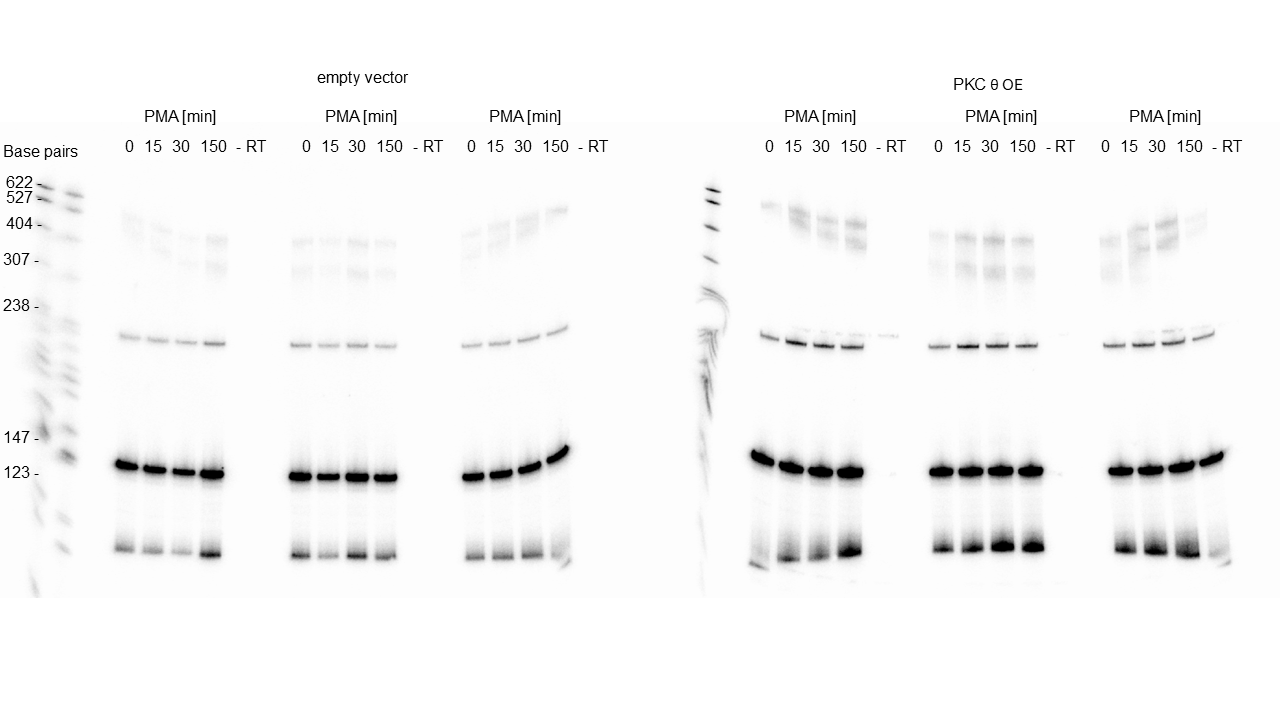

Supplement: Supplementary file 9 — Source data Fig. 5 [file 44318_2025_374_MOESM9_ESM.zip › EMBOJ-2024-118552_Source data_Fig. 5/5D/5D/Slide1.TIF]

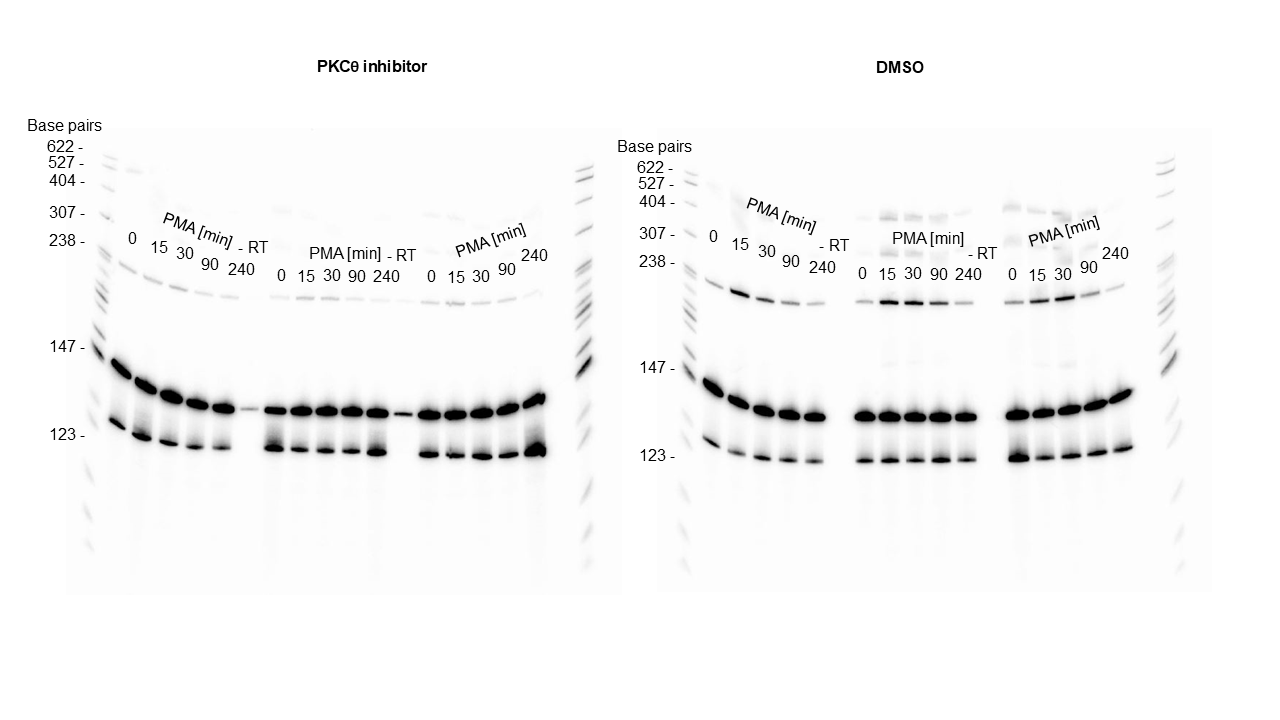

Supplement: Supplementary file 9 — Source data Fig. 5 [file 44318_2025_374_MOESM9_ESM.zip › EMBOJ-2024-118552_Source data_Fig. 5/5A/5A.TIF]

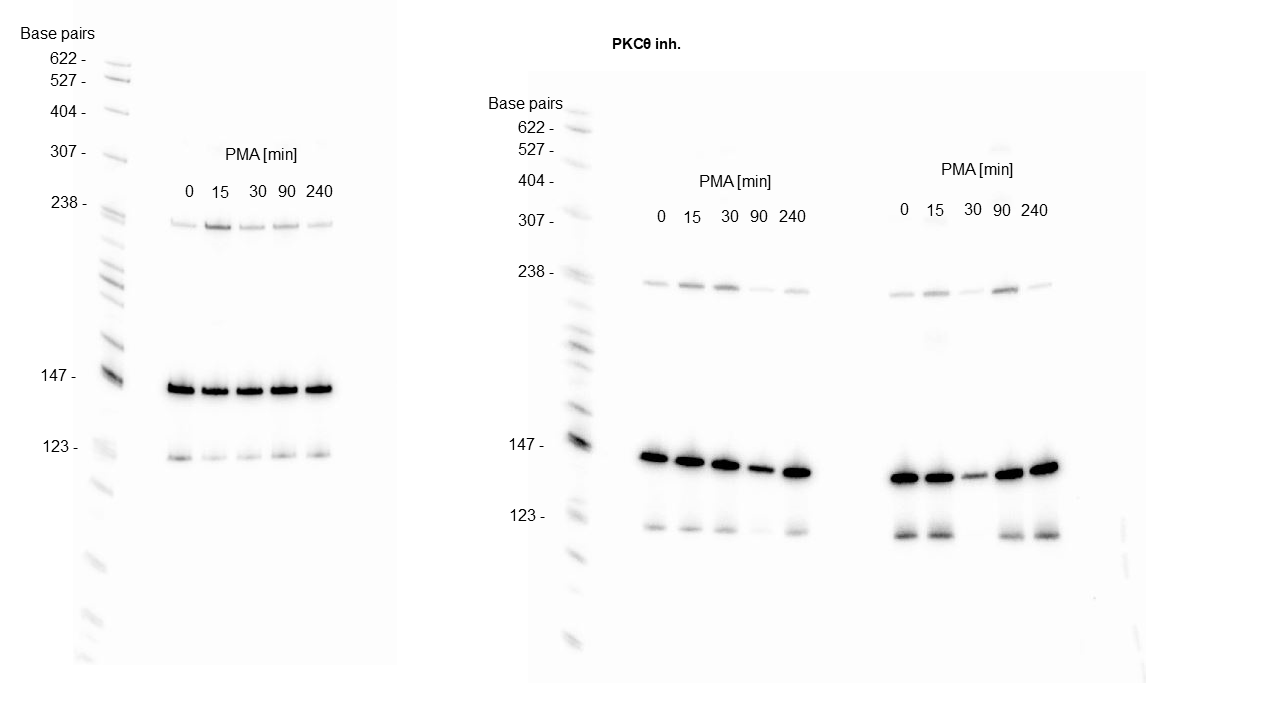

Supplement: Supplementary file 9 — Source data Fig. 5 [file 44318_2025_374_MOESM9_ESM.zip › EMBOJ-2024-118552_Source data_Fig. 5/5B/5B/Slide2.TIF]

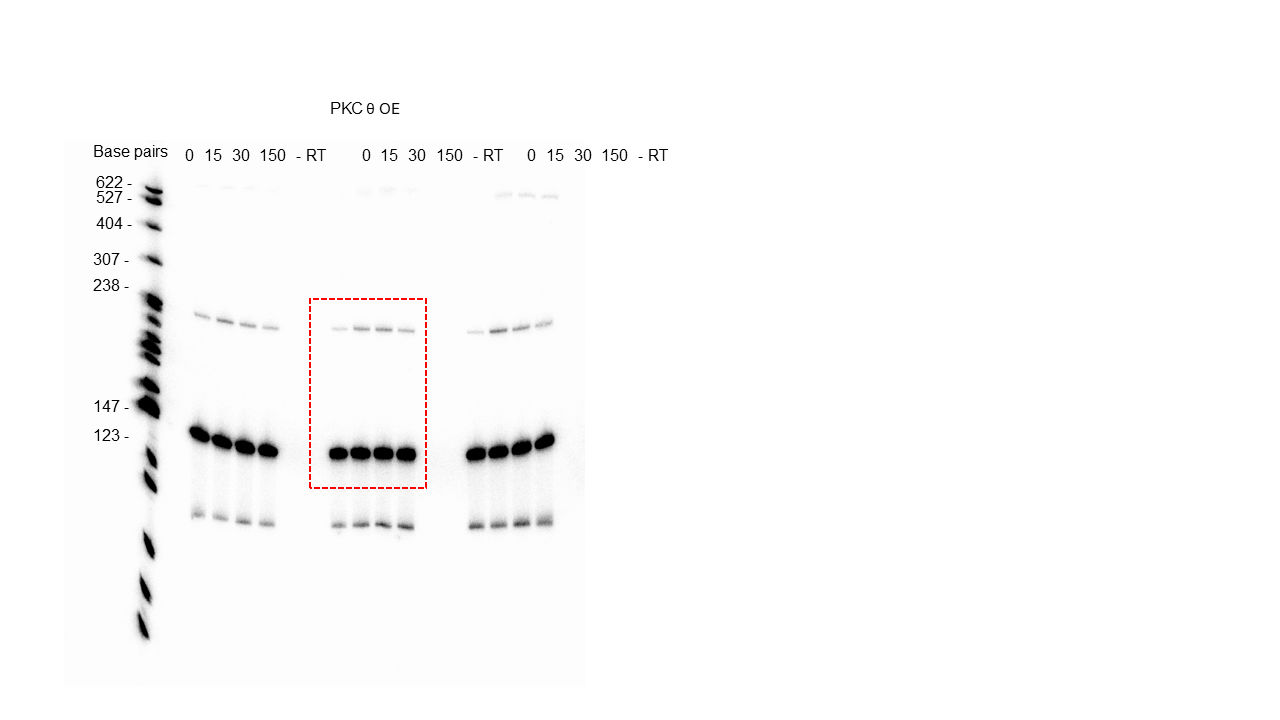

Supplement: Supplementary file 9 — Source data Fig. 5 [file 44318_2025_374_MOESM9_ESM.zip › EMBOJ-2024-118552_Source data_Fig. 5/5G/5G/Slide1.TIF]

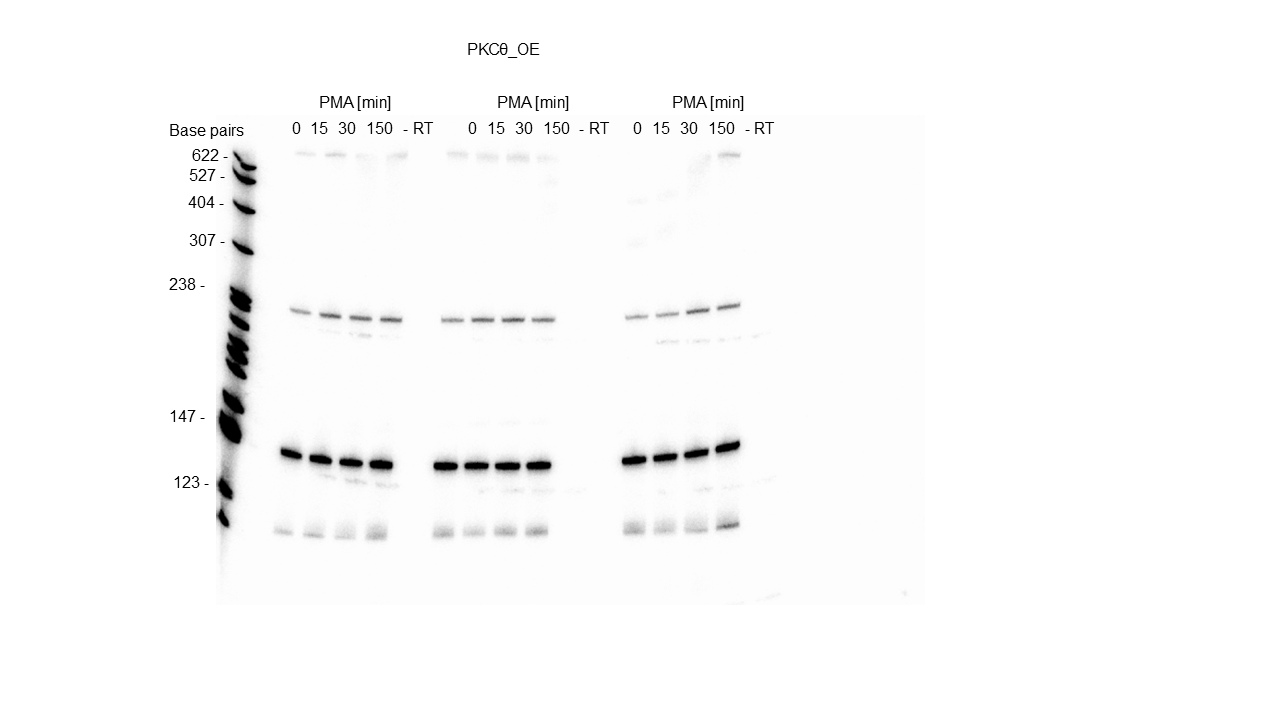

Supplement: Supplementary file 9 — Source data Fig. 5 [file 44318_2025_374_MOESM9_ESM.zip › EMBOJ-2024-118552_Source data_Fig. 5/5E/5E/Slide1.TIF]

### PKC $\theta$ inhibitor

Base pairs

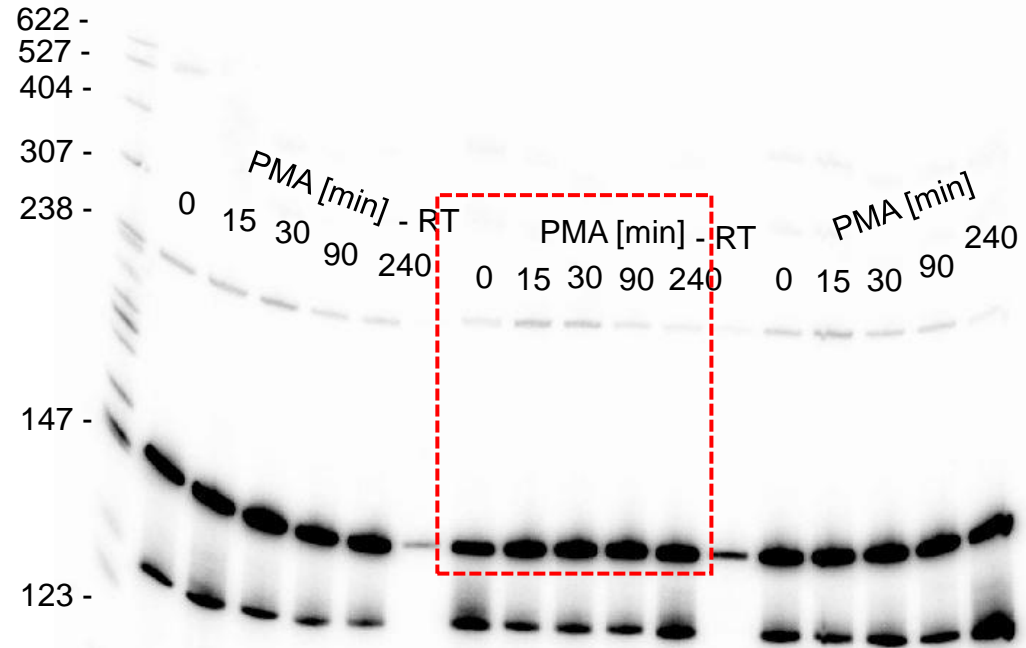

### DMSO

Base pairs

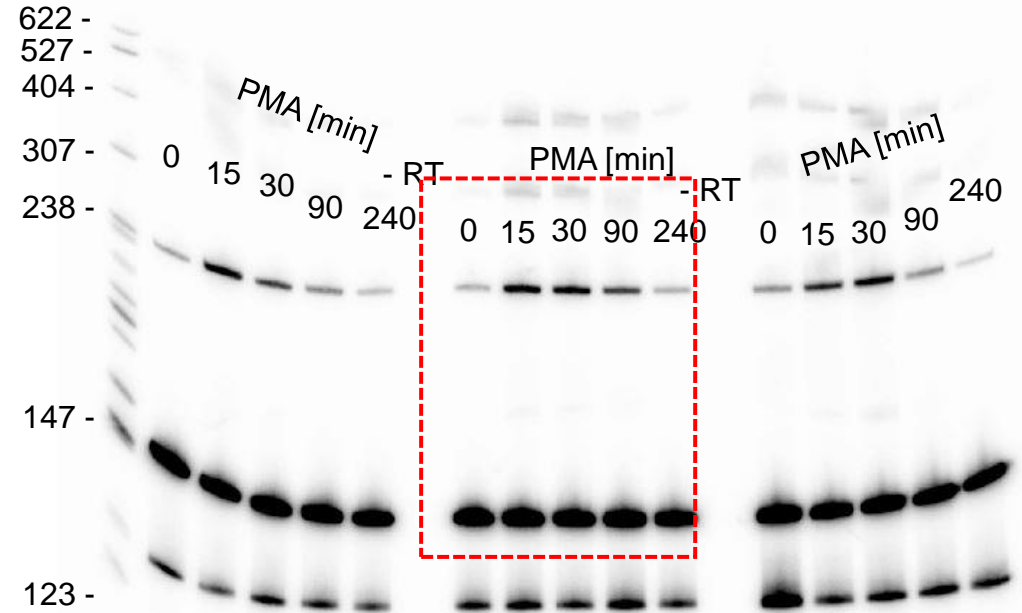

Supplement: Supplementary file 9 — Source data Fig. 5 [file 44318_2025_374_MOESM9_ESM.zip › EMBOJ-2024-118552_Source data_Fig. 5/5A/5A.pdf]

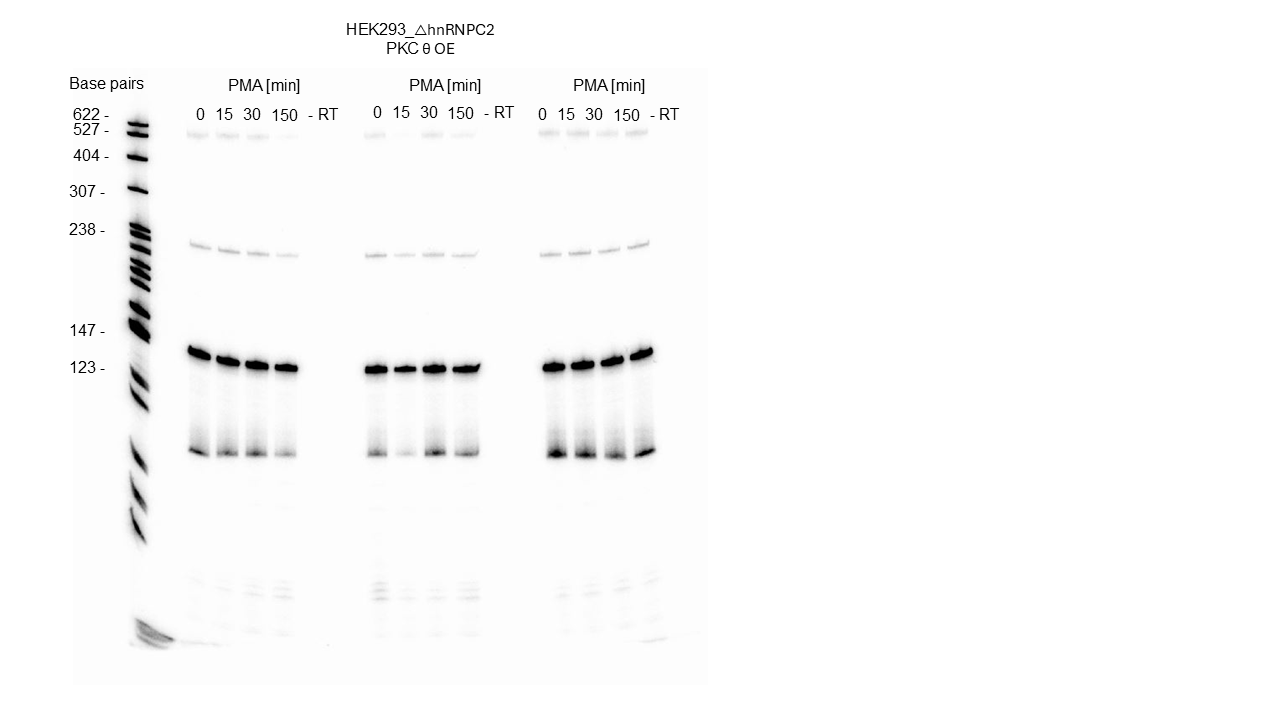

Supplement: Supplementary file 9 — Source data Fig. 5 [file 44318_2025_374_MOESM9_ESM.zip › EMBOJ-2024-118552_Source data_Fig. 5/5D/5D/Slide2.TIF]

PKCθ\_OE

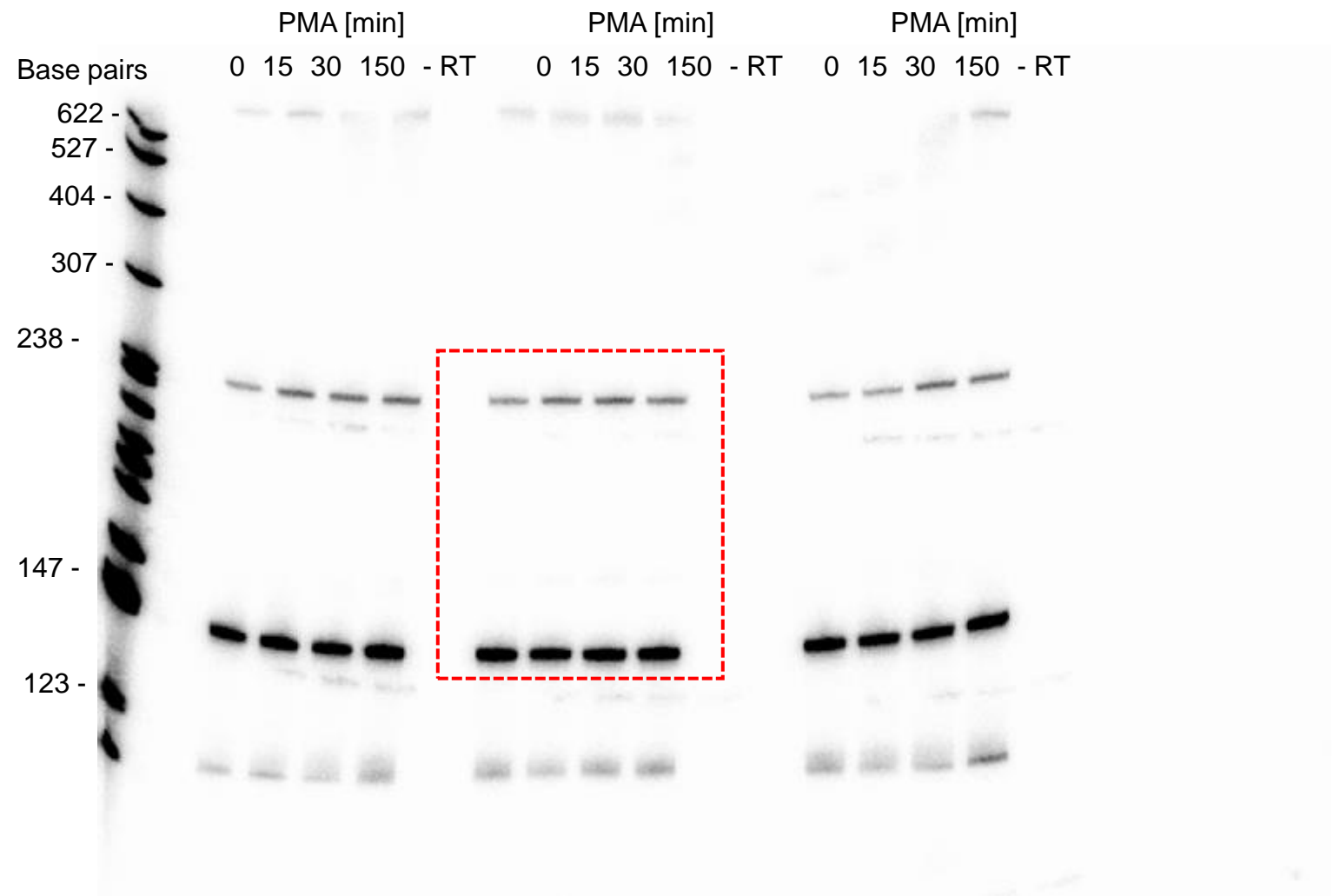

HEK WT  
empty vector

HEK293 hnRNP C2  
PKC  $\theta$  OE

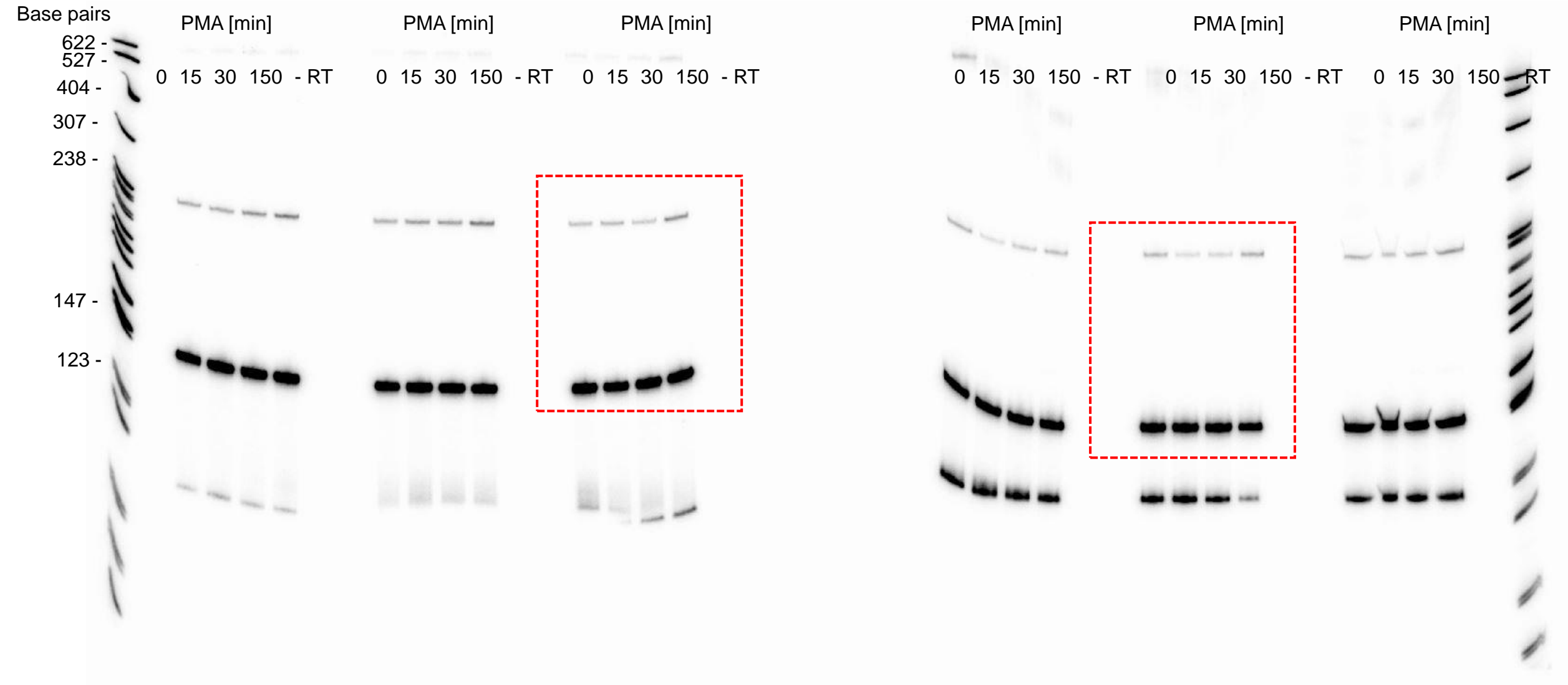

Supplement: Supplementary file 9 — Source data Fig. 5 [file 44318_2025_374_MOESM9_ESM.zip › EMBOJ-2024-118552_Source data_Fig. 5/5E/5E.pdf]

PKC  $\theta$  OE

Base pairs    0   15   30   150   - RT       0   15   30   150   - RT       0   15   30   150   - RT

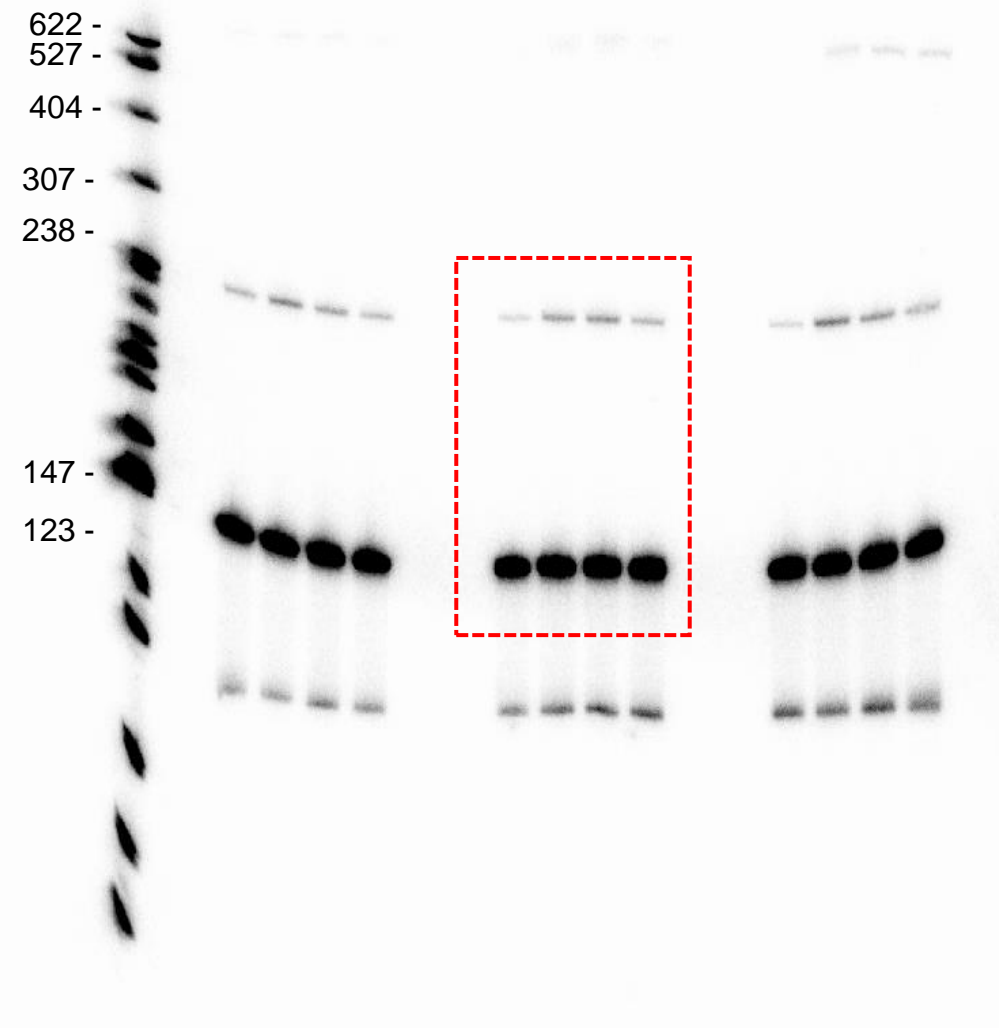

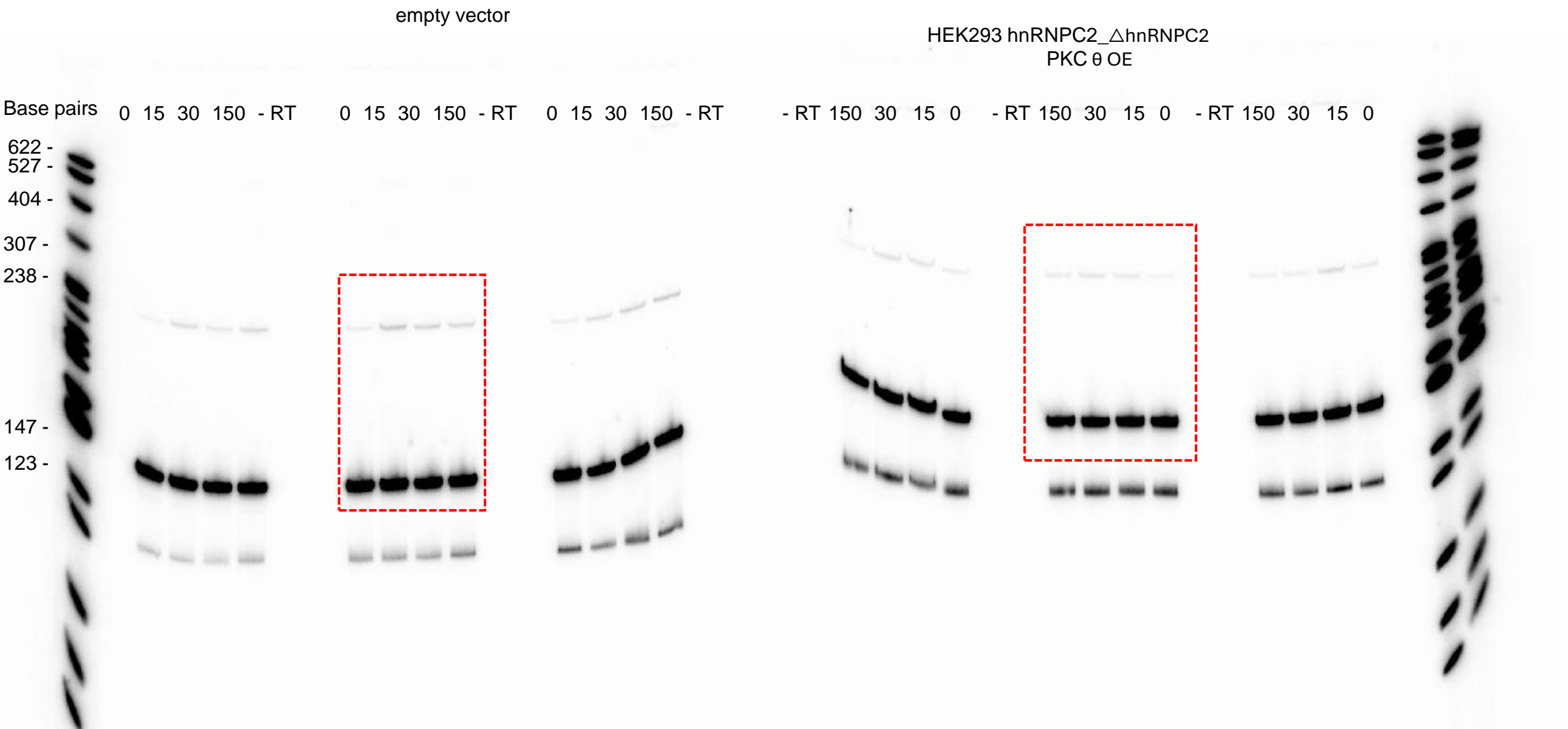

Supplement: Supplementary file 9 — Source data Fig. 5 [file 44318_2025_374_MOESM9_ESM.zip › EMBOJ-2024-118552_Source data_Fig. 5/5G/5G.pdf]

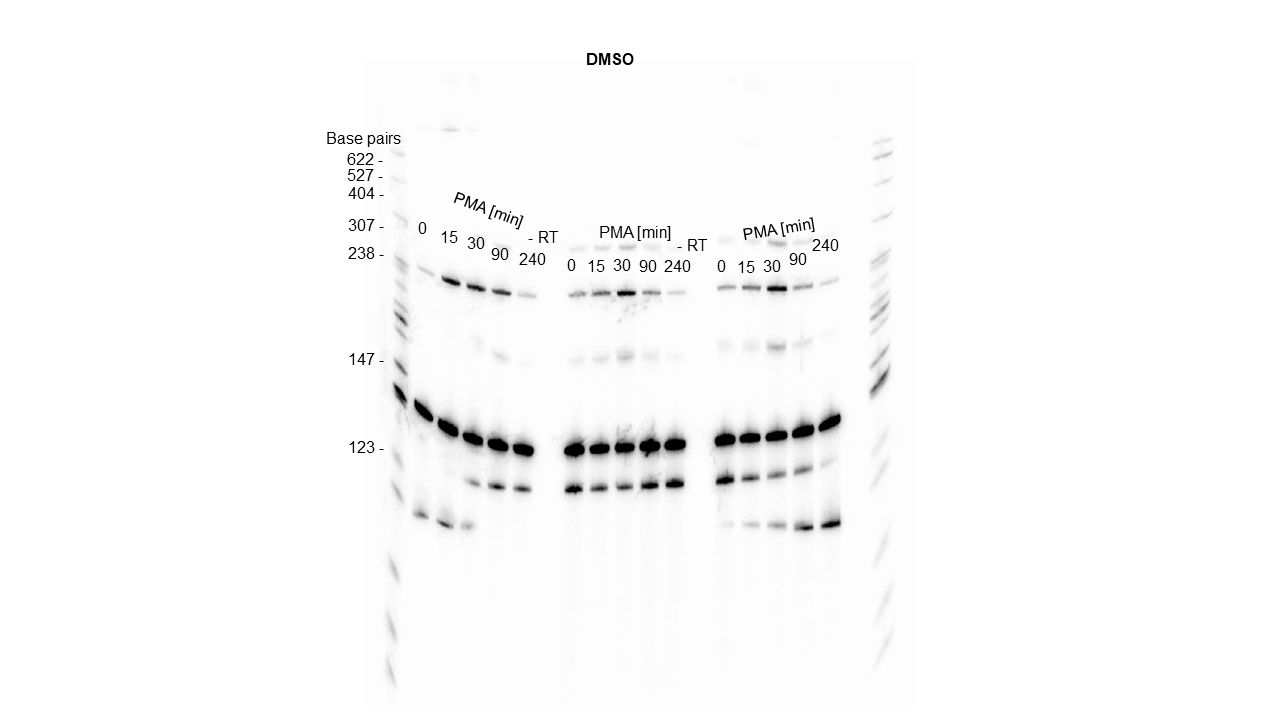

Supplement: Supplementary file 9 — Source data Fig. 5 [file 44318_2025_374_MOESM9_ESM.zip › EMBOJ-2024-118552_Source data_Fig. 5/5B/5B/Slide1.TIF]

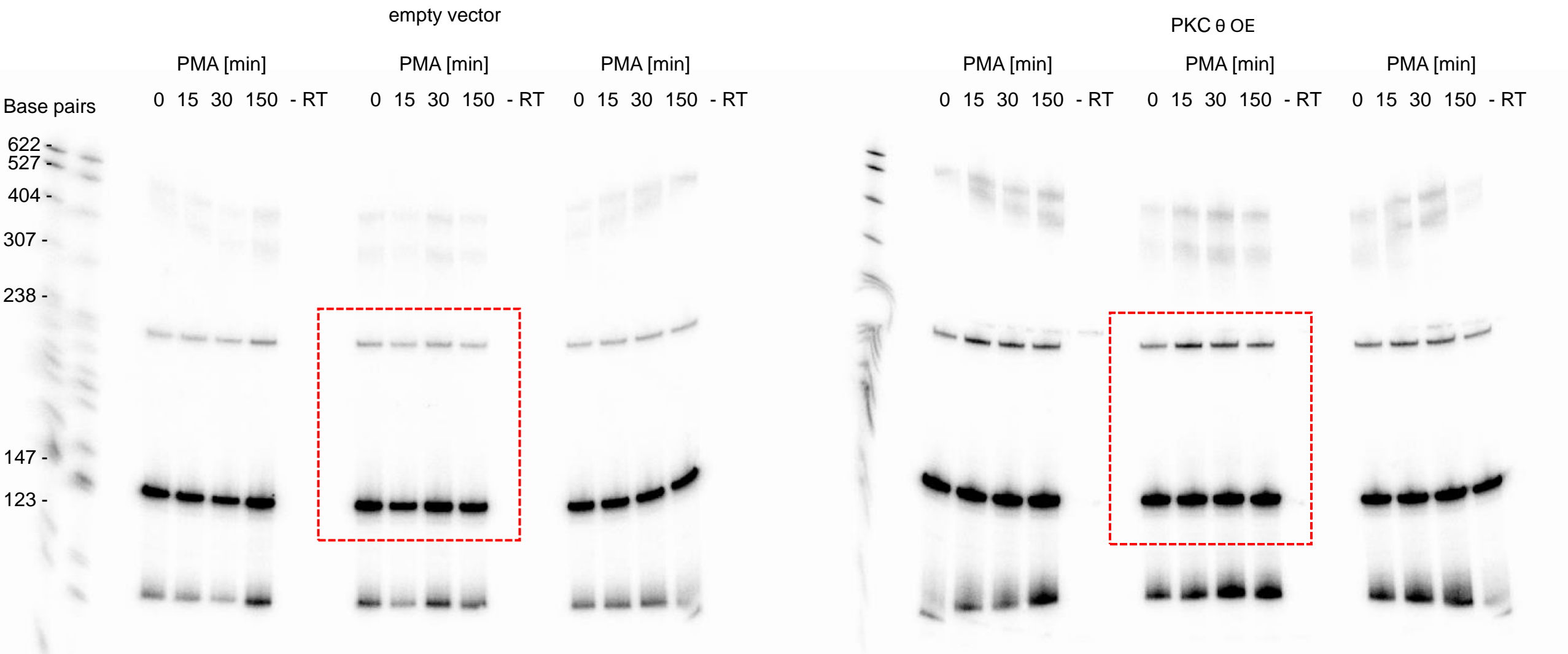

HEK293\_ΔhnRNP2  
PKC θ OE

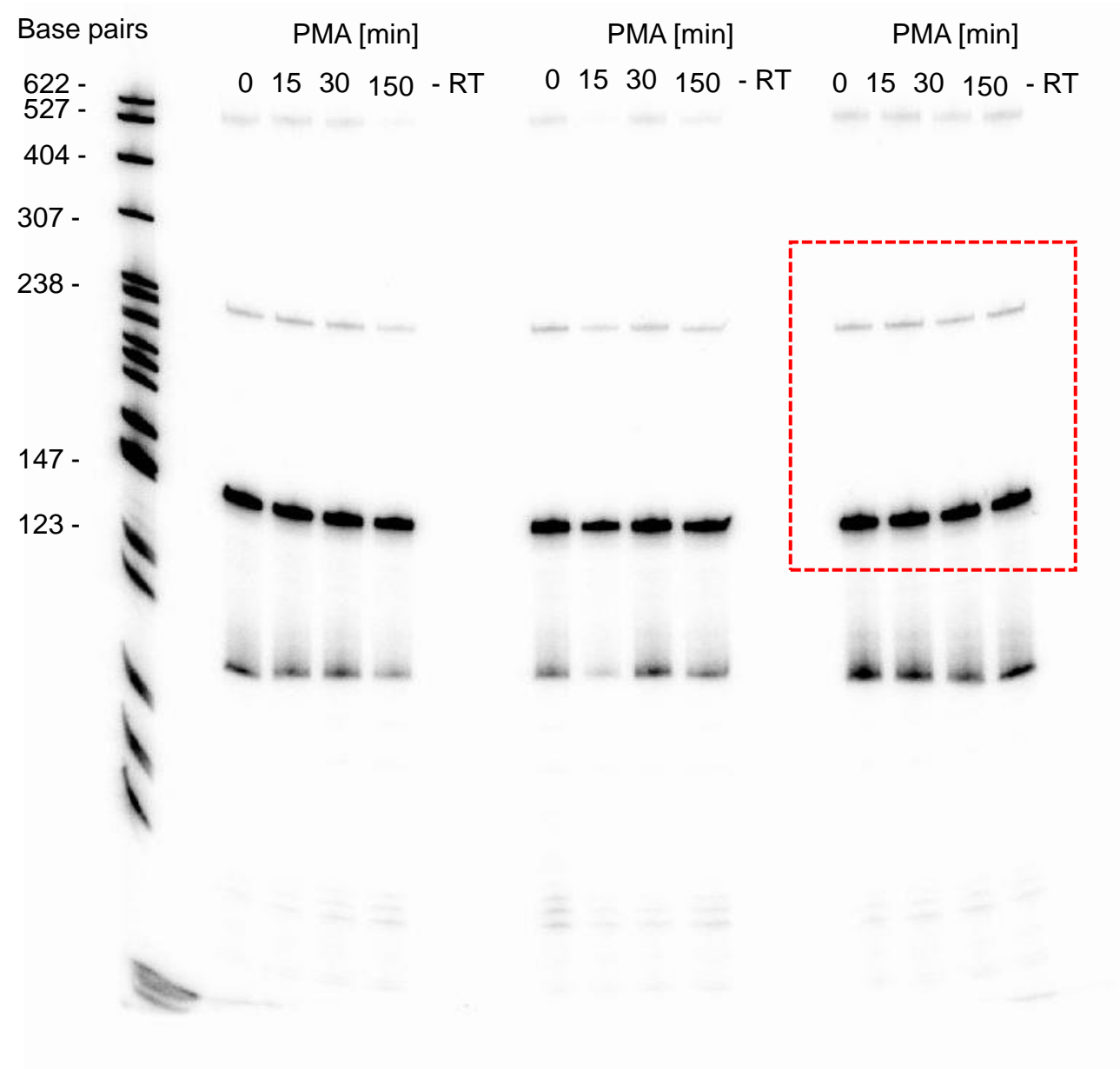

Supplement: Supplementary file 9 — Source data Fig. 5 [file 44318_2025_374_MOESM9_ESM.zip › EMBOJ-2024-118552_Source data_Fig. 5/5D/5D.pdf]

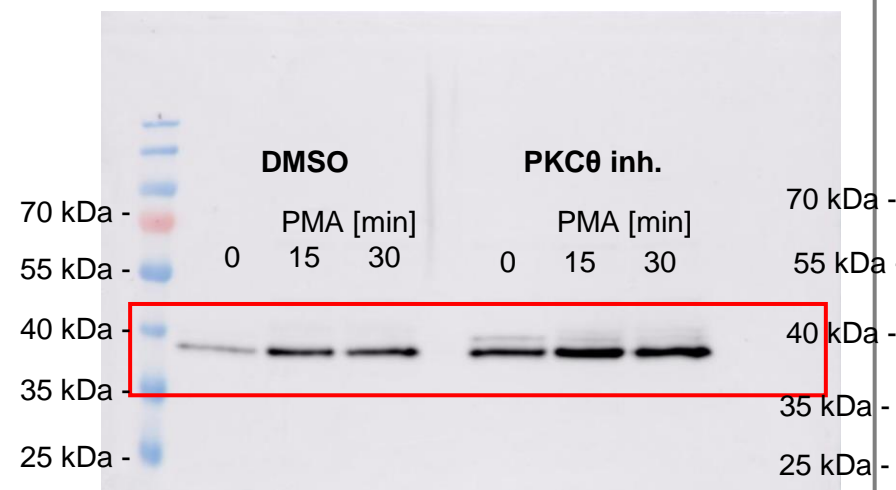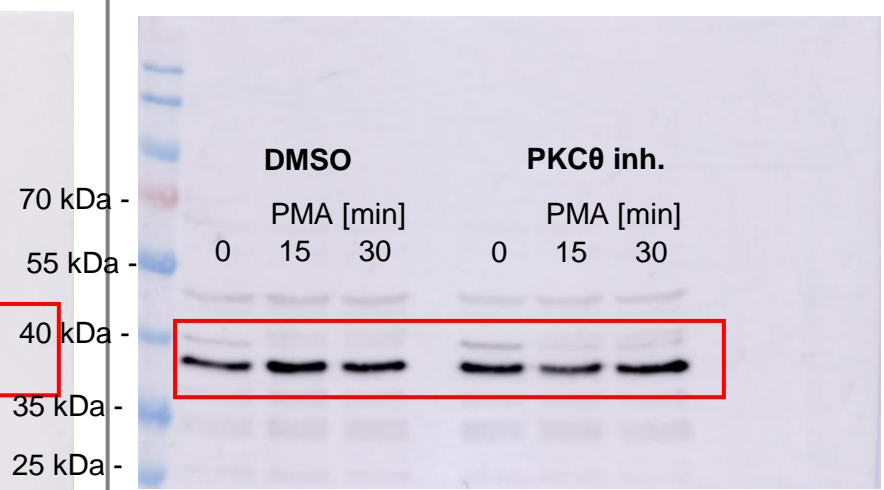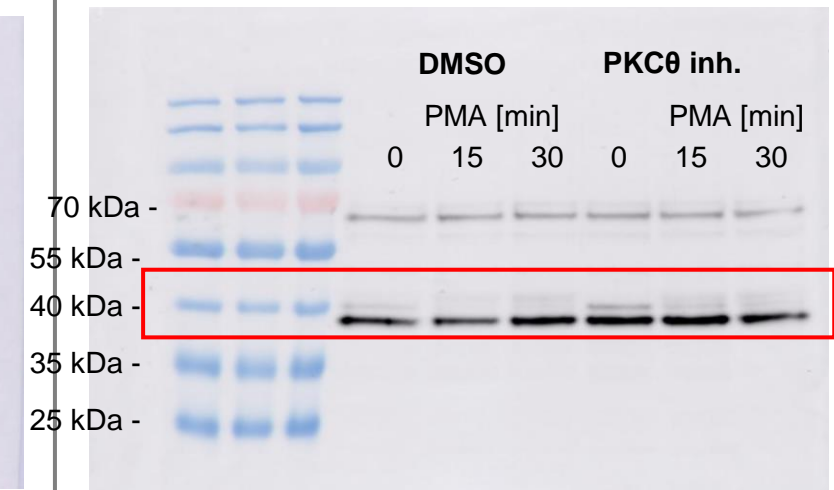

Supplement: Supplementary file 9 — Source data Fig. 5 [file 44318_2025_374_MOESM9_ESM.zip › EMBOJ-2024-118552_Source data_Fig. 5/5C/5C.pdf]

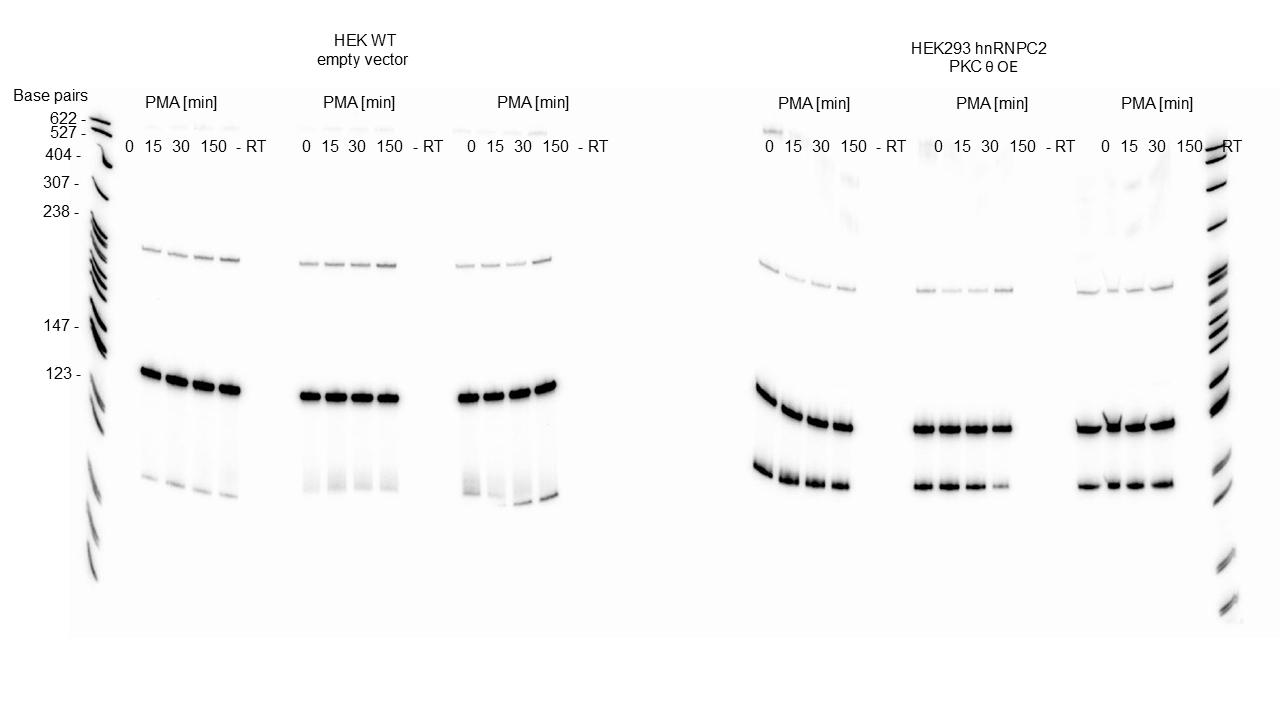

Supplement: Supplementary file 9 — Source data Fig. 5 [file 44318_2025_374_MOESM9_ESM.zip › EMBOJ-2024-118552_Source data_Fig. 5/5E/5E/Slide2.TIF]

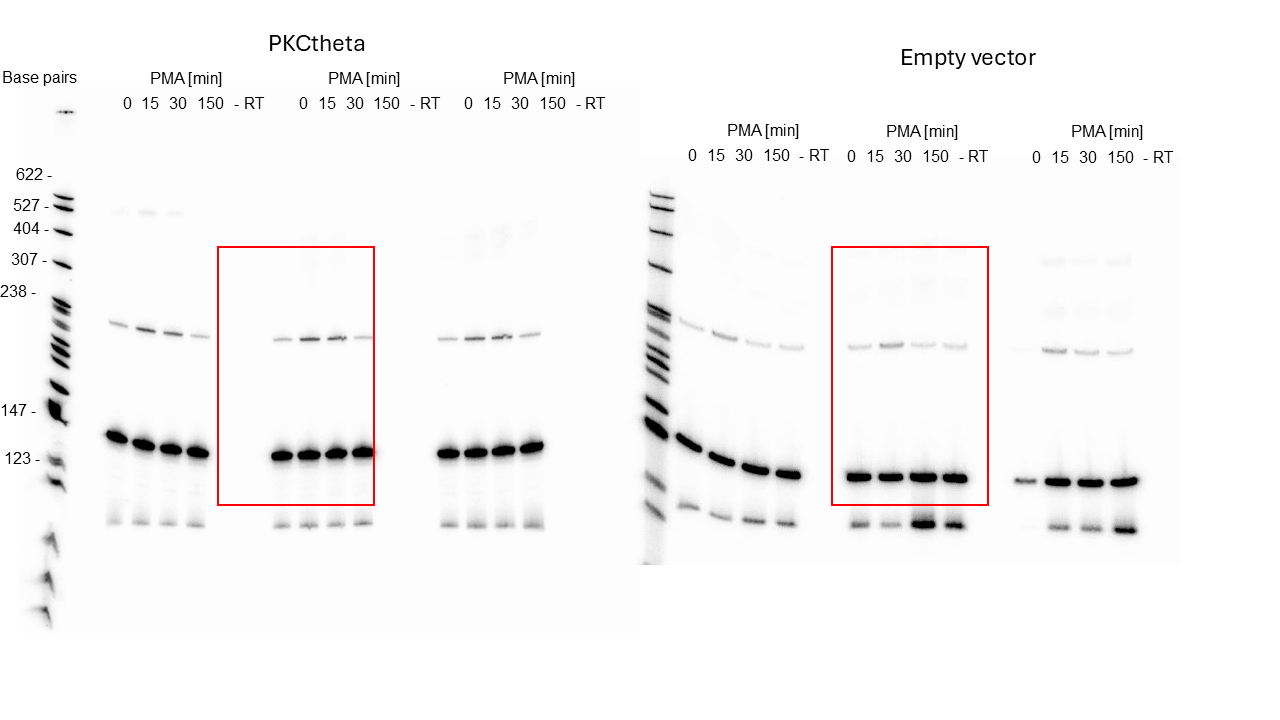

Supplement: Supplementary file 9 — Source data Fig. 5 [file 44318_2025_374_MOESM9_ESM.zip › EMBOJ-2024-118552_Source data_Fig. 5/5F/5F/Slide1.TIF]

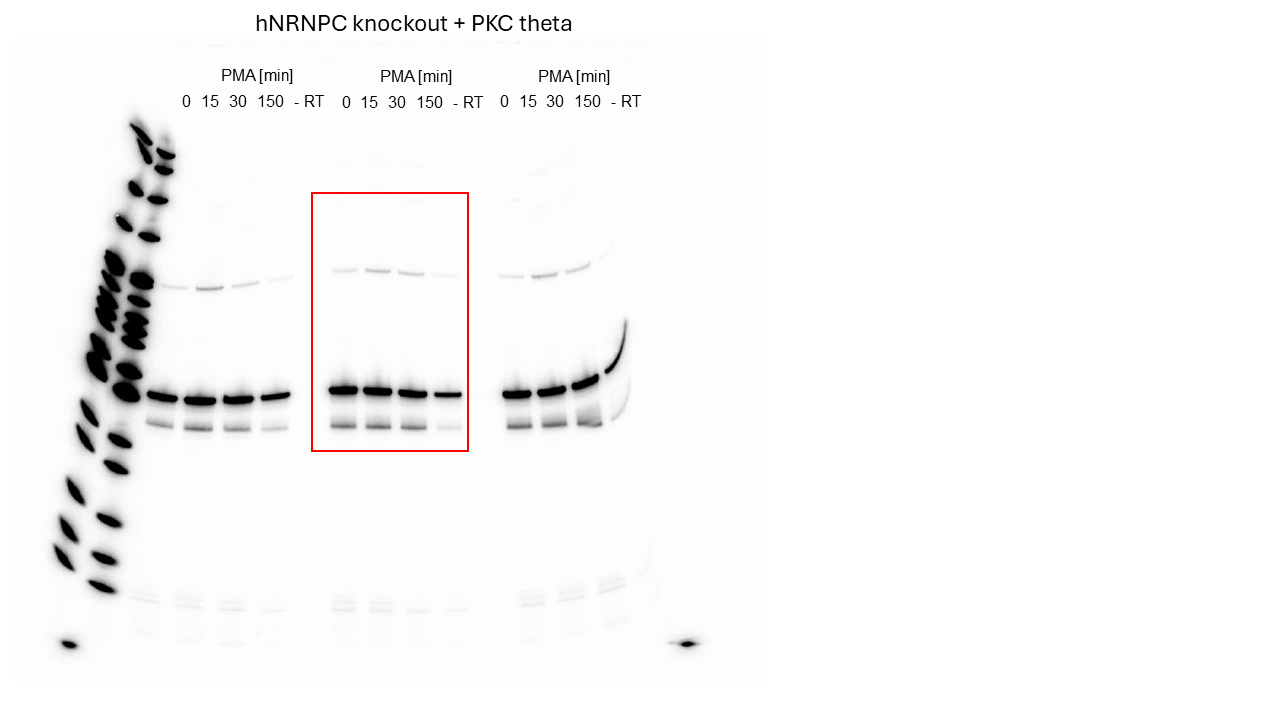

Supplement: Supplementary file 9 — Source data Fig. 5 [file 44318_2025_374_MOESM9_ESM.zip › EMBOJ-2024-118552_Source data_Fig. 5/5F/5F/Slide2.TIF]

PKC  $\theta$  OE

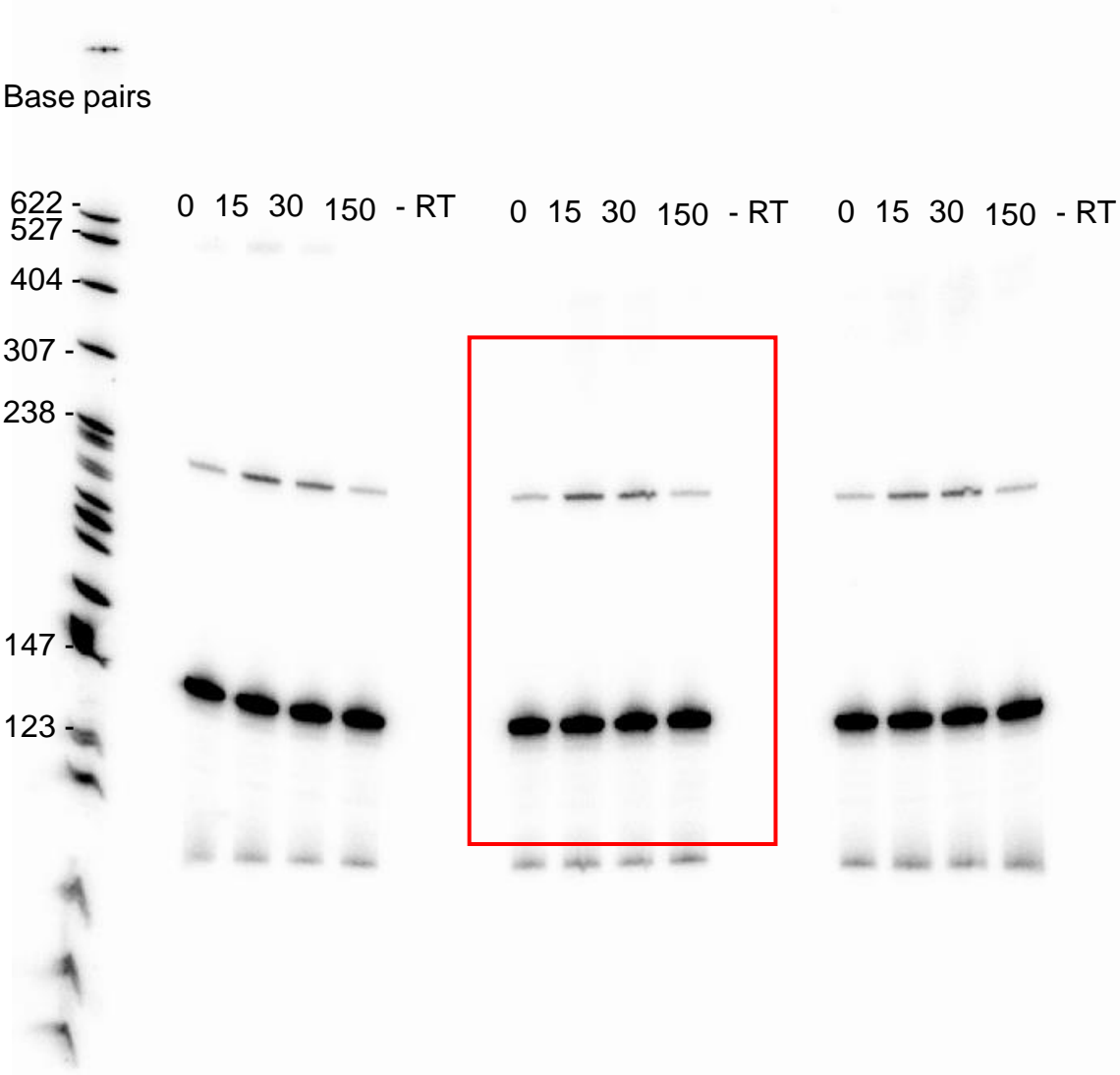

Empty vector

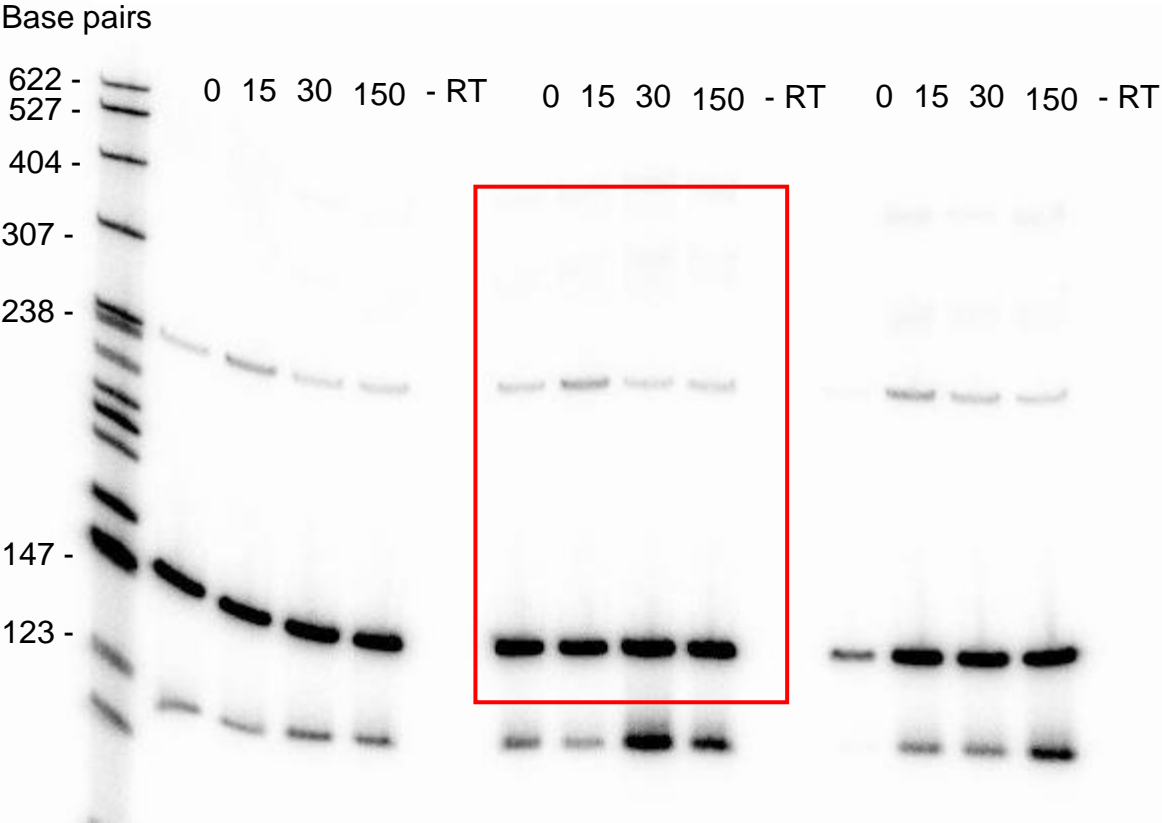

Base pairs

HEK293\_ΔhnRNP2  
PKC θ OE

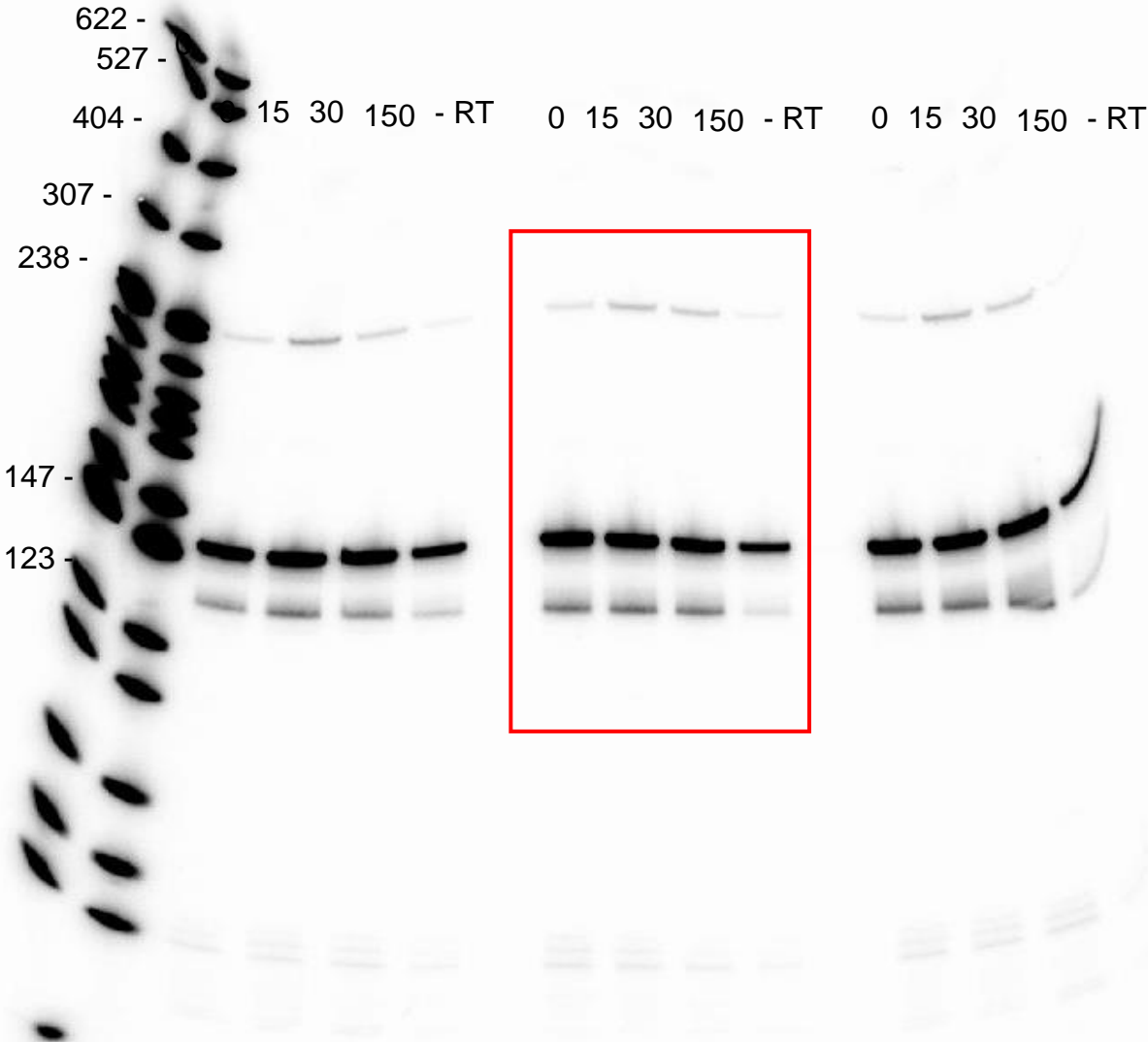

Supplement: Supplementary file 9 — Source data Fig. 5 [file 44318_2025_374_MOESM9_ESM.zip › EMBOJ-2024-118552_Source data_Fig. 5/5F/5F.pdf]

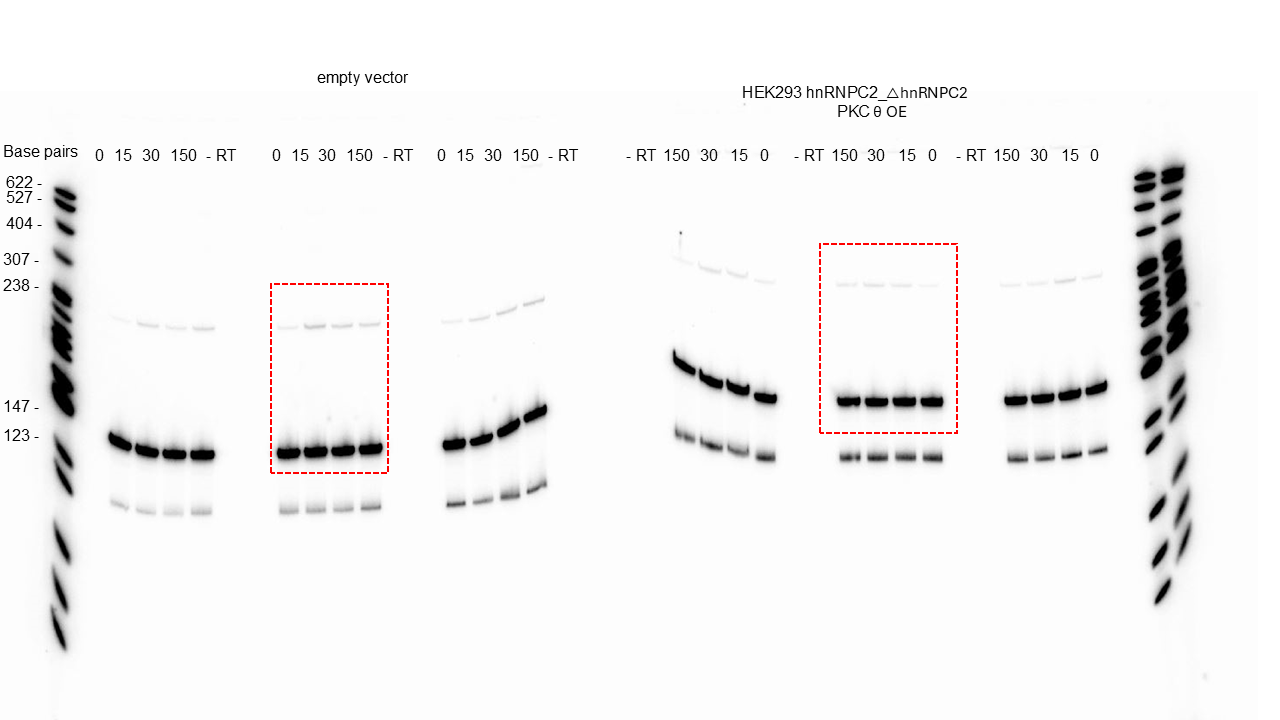

Supplement: Supplementary file 9 — Source data Fig. 5 [file 44318_2025_374_MOESM9_ESM.zip › EMBOJ-2024-118552_Source data_Fig. 5/5G/5G/Slide2.TIF]

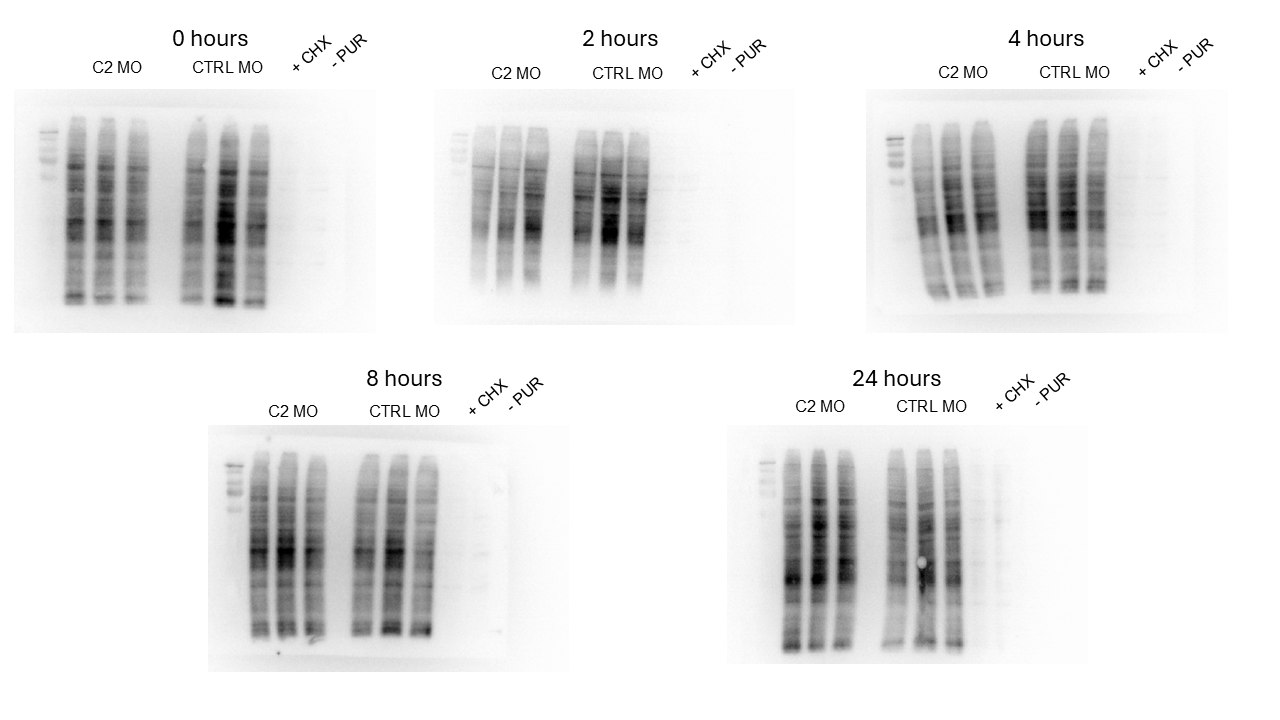

Supplement: Supplementary file 10 — Source data Fig. 6 [file 44318_2025_374_MOESM10_ESM.zip › EMBOJ-2024-118552_Source data_Fig. 6/6B/6B.tif]

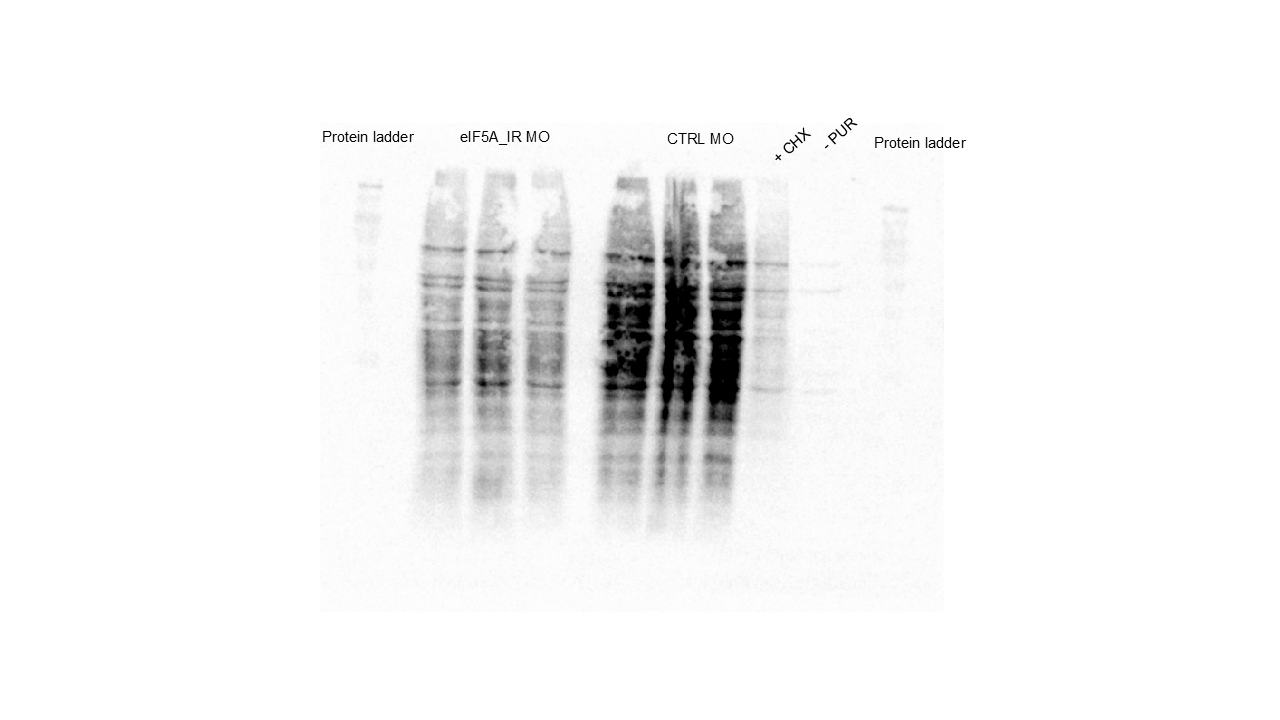

Supplement: Supplementary file 10 — Source data Fig. 6 [file 44318_2025_374_MOESM10_ESM.zip › EMBOJ-2024-118552_Source data_Fig. 6/6F/6F.tif]

EIF5A\_IR

EIF5A\_WT

Empty vector

70kDa -

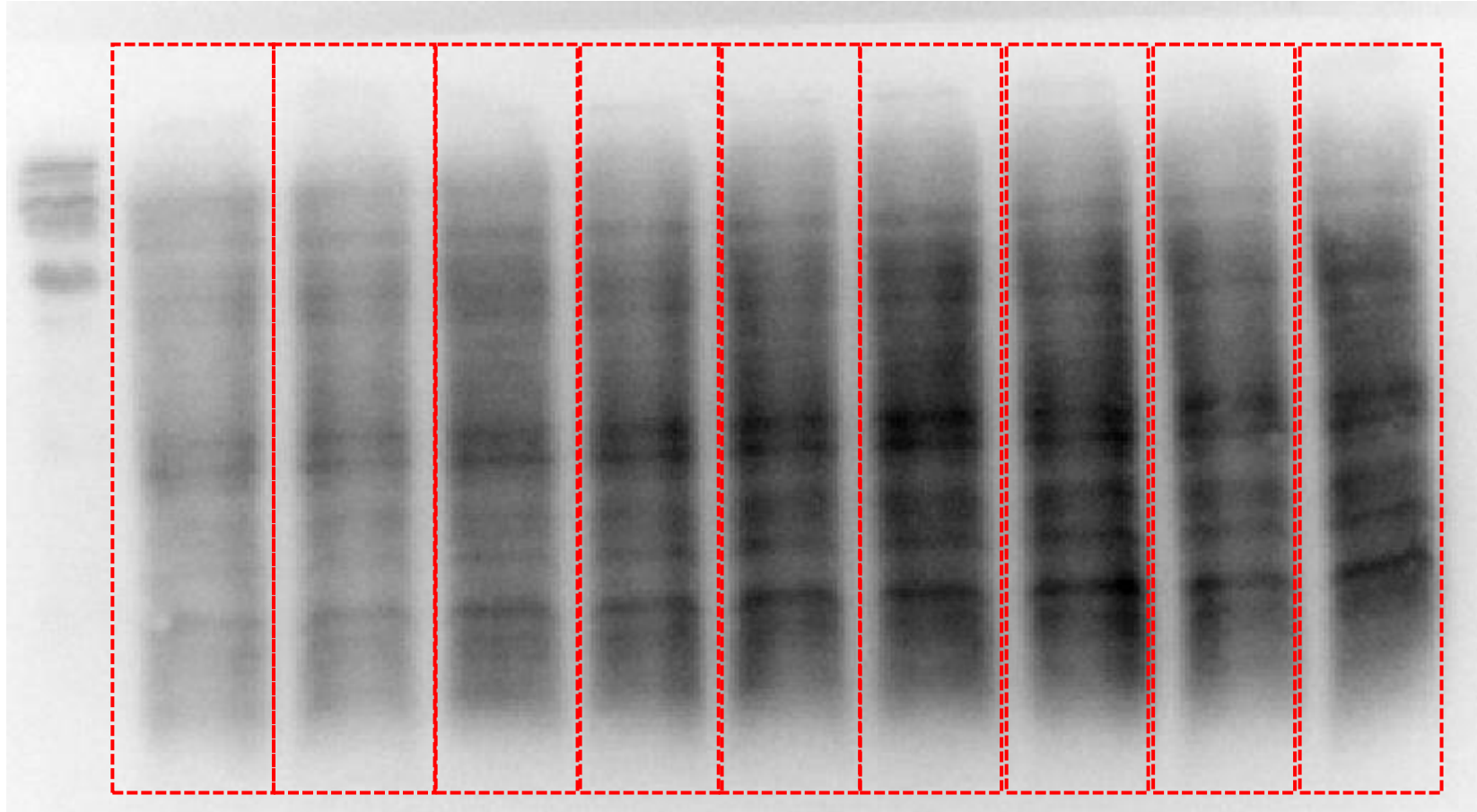

Supplement: Supplementary file 10 — Source data Fig. 6 [file 44318_2025_374_MOESM10_ESM.zip › EMBOJ-2024-118552_Source data_Fig. 6/6D/6D.pdf]

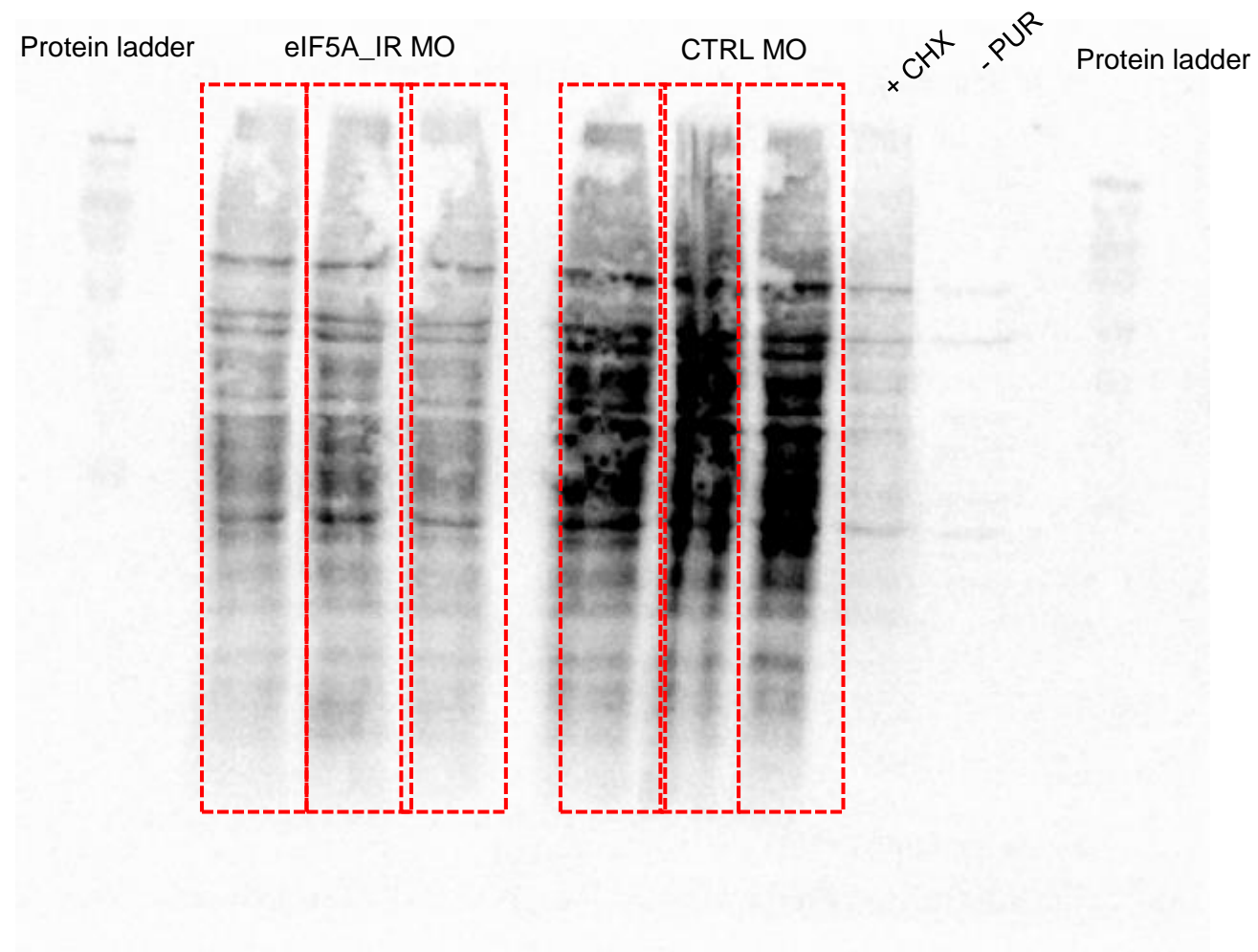

Supplement: Supplementary file 10 — Source data Fig. 6 [file 44318_2025_374_MOESM10_ESM.zip › EMBOJ-2024-118552_Source data_Fig. 6/6F/6F.pdf]

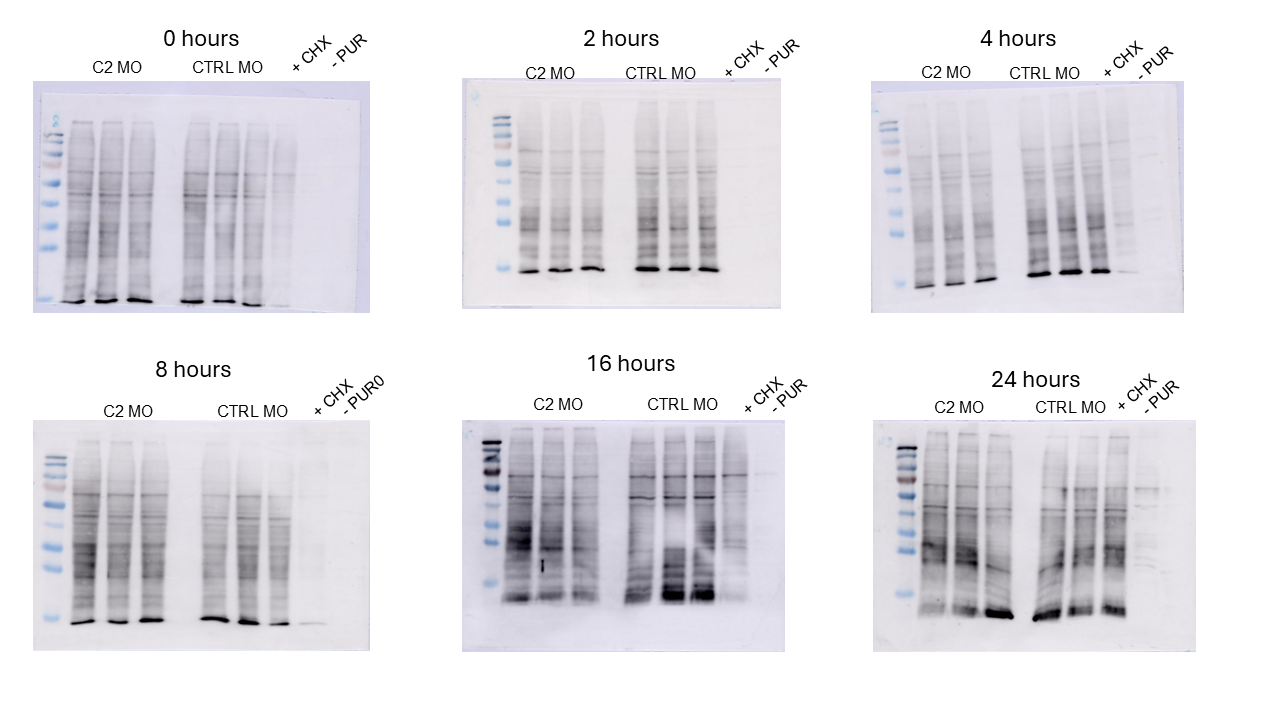

Supplement: Supplementary file 10 — Source data Fig. 6 [file 44318_2025_374_MOESM10_ESM.zip › EMBOJ-2024-118552_Source data_Fig. 6/6A/6A.TIF]

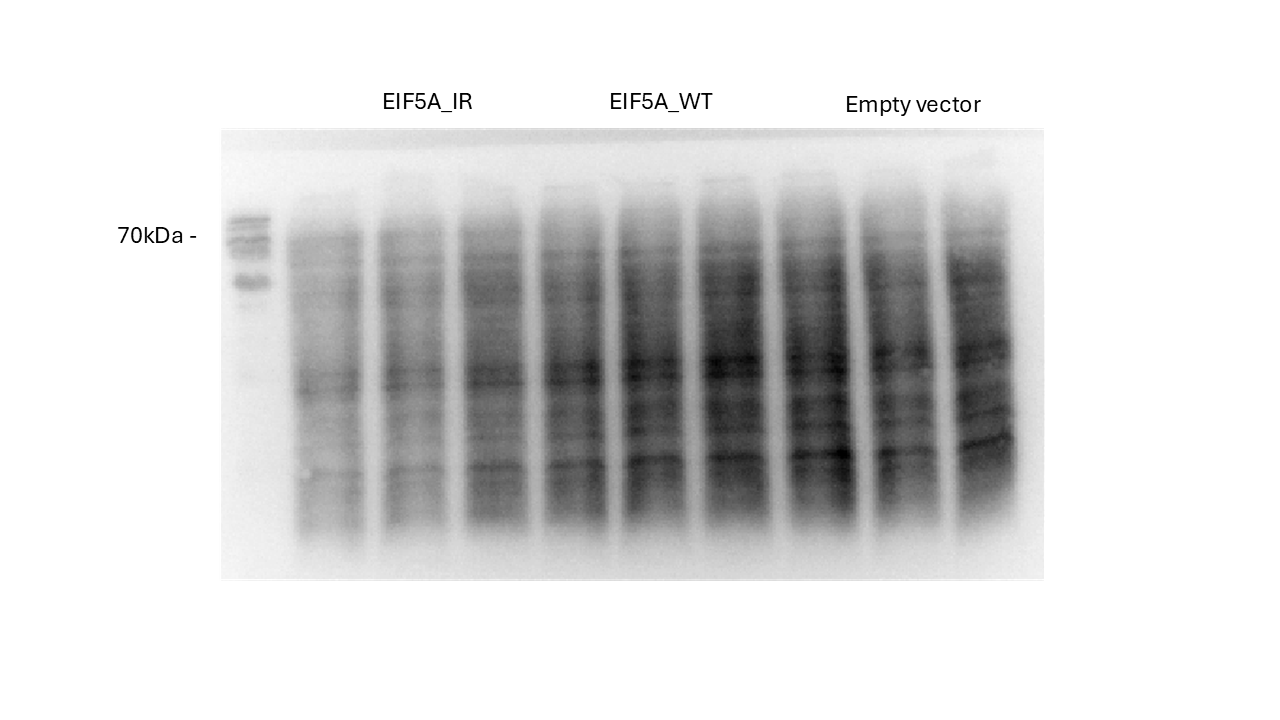

Supplement: Supplementary file 10 — Source data Fig. 6 [file 44318_2025_374_MOESM10_ESM.zip › EMBOJ-2024-118552_Source data_Fig. 6/6D/6D.tif]

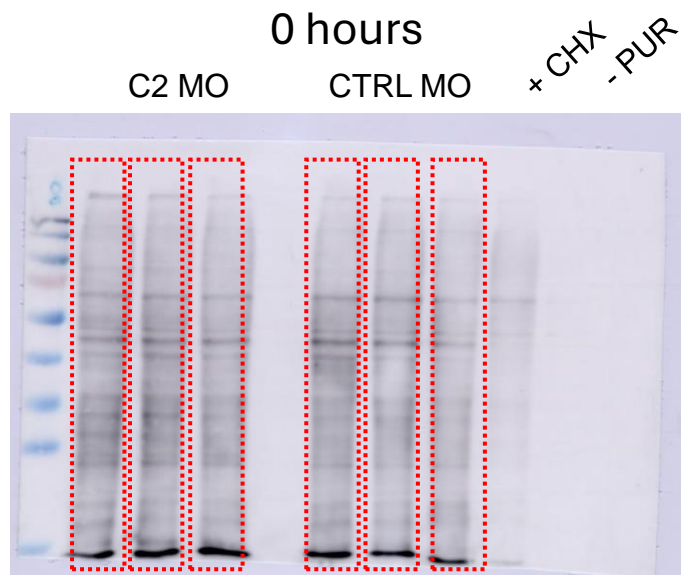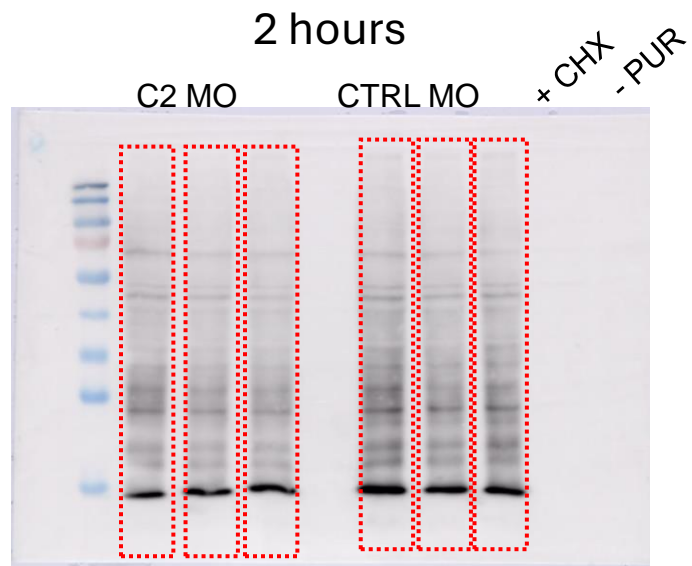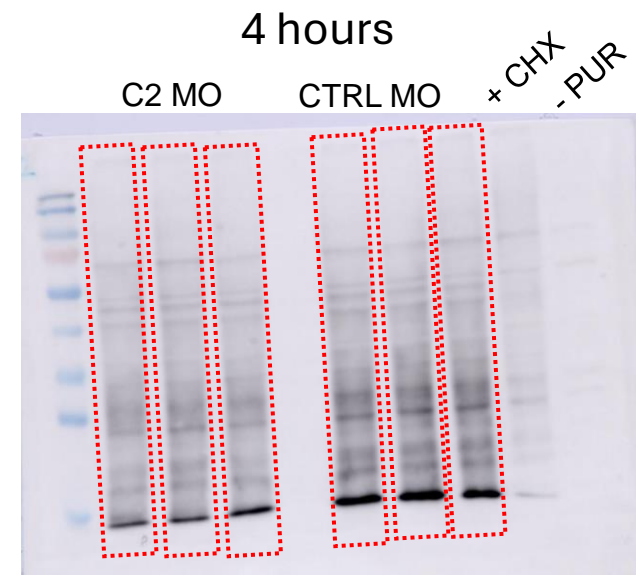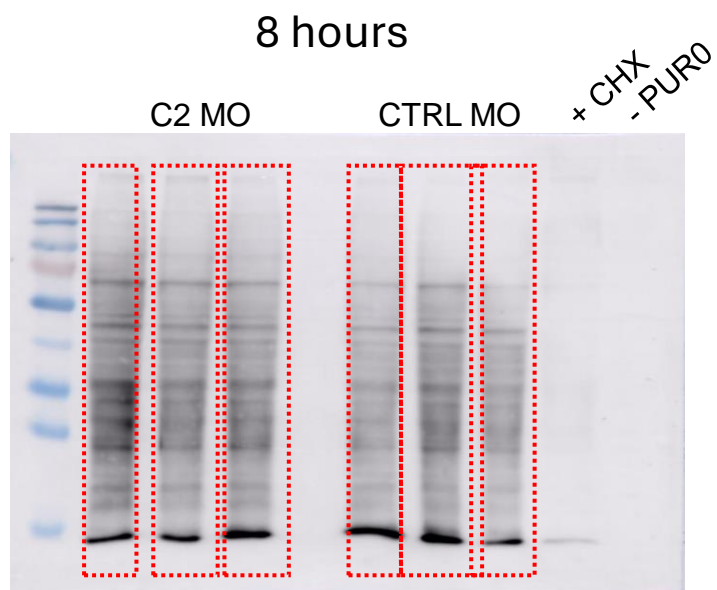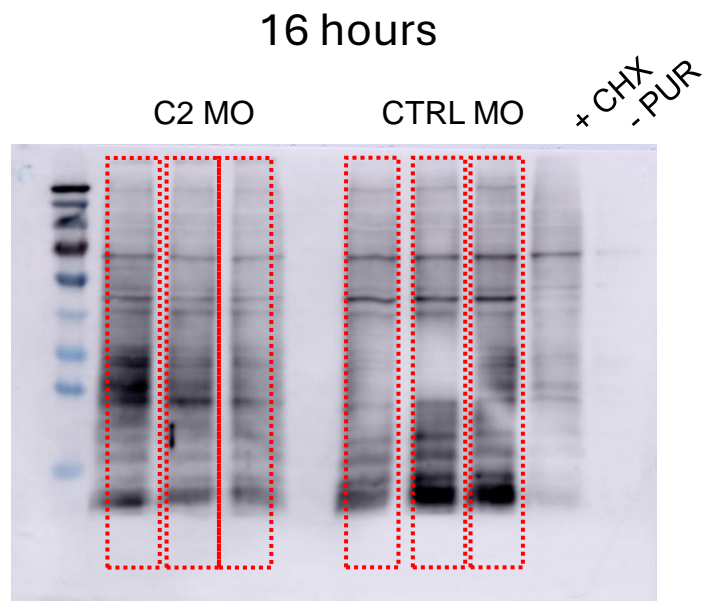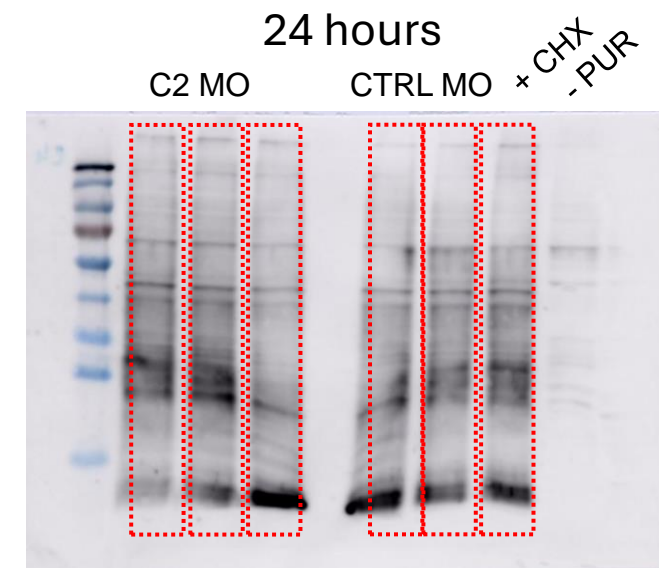

Supplement: Supplementary file 10 — Source data Fig. 6 [file 44318_2025_374_MOESM10_ESM.zip › EMBOJ-2024-118552_Source data_Fig. 6/6A/6A.pdf]

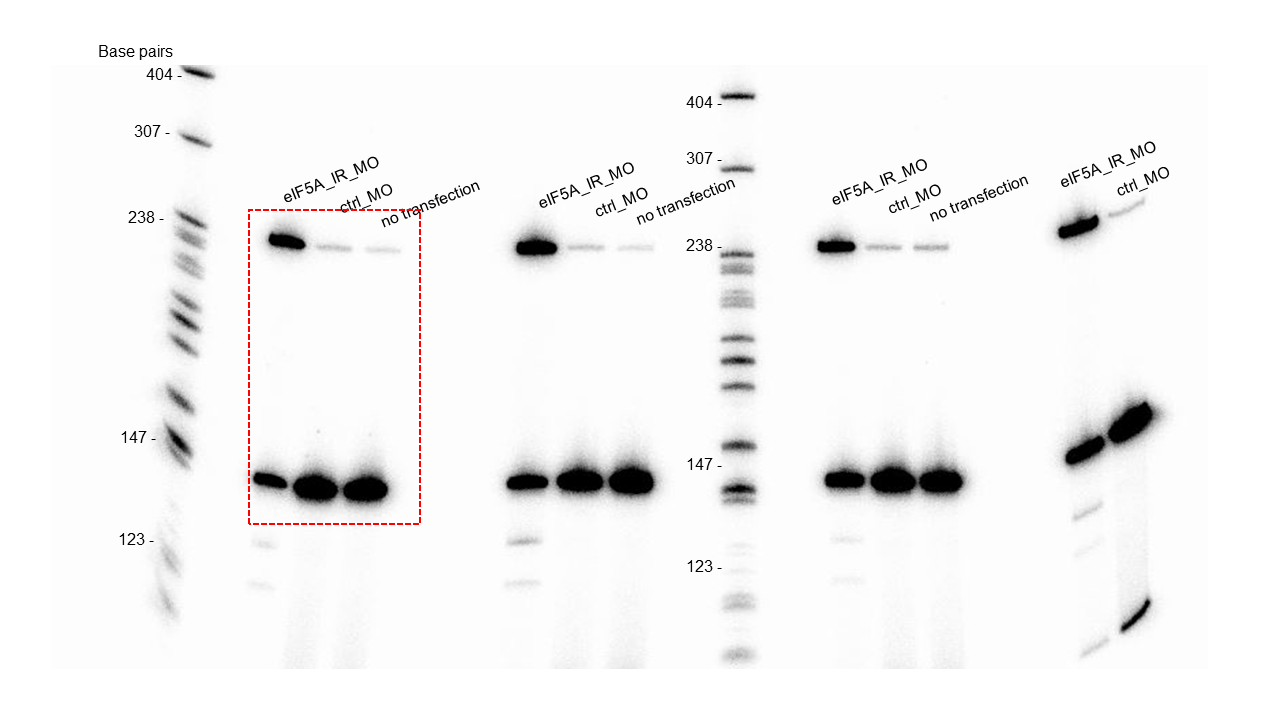

Supplement: Supplementary file 10 — Source data Fig. 6 [file 44318_2025_374_MOESM10_ESM.zip › EMBOJ-2024-118552_Source data_Fig. 6/6E/6E.tif]
