# Supplementary material for: Immediate early splicing controls translation in activated T-cells and is mediated by hnRNPC2 phosphorylation
Source: EMBO J. 2025 Feb 13;44(6):1692–723. doi: 10.1038/s44318-025-00374-8 (PMC11914300; doi:10.1038/s44318-025-00374-8)
Supplement: Supplementary file 6 — Source data Fig. 2 [file 44318_2025_374_MOESM6_ESM.zip › EMBOJ-2024-118552_Source data_Fig. 2/2E/2E.pdf]

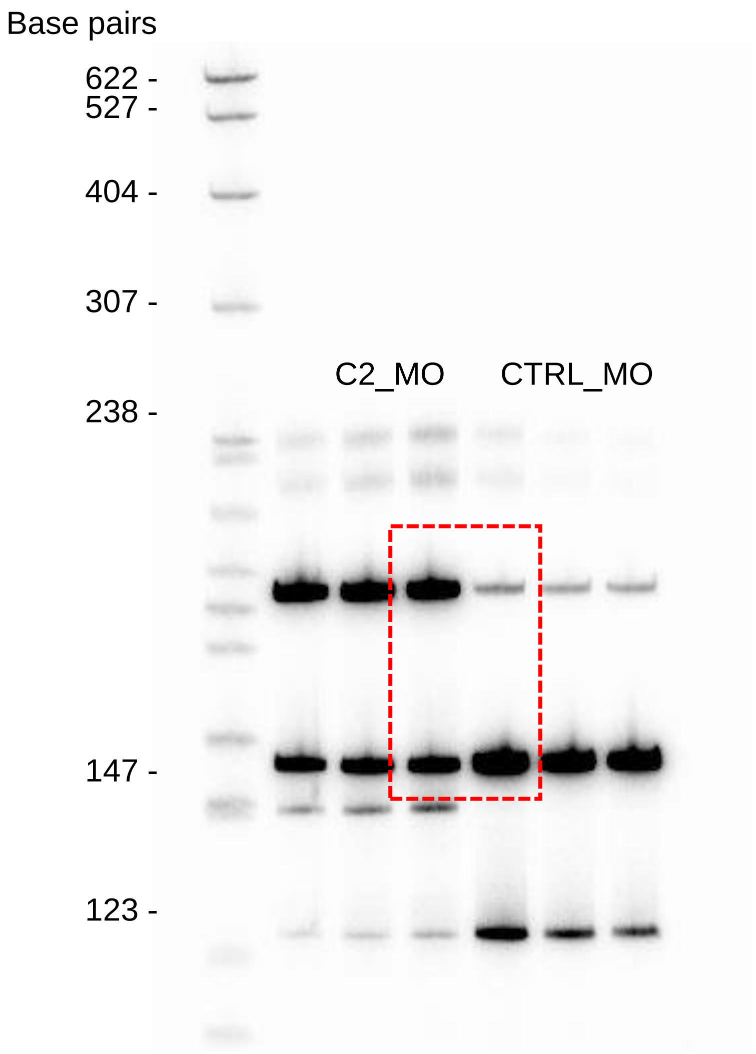
Base pairs
622 -
527 -
404 -
307 -
238 -
147 -
123 -
C2_MO
CTRL_MO
