# Supplementary material for: Immediate early splicing controls translation in activated T-cells and is mediated by hnRNPC2 phosphorylation
Source: EMBO J. 2025 Feb 13;44(6):1692–723. doi: 10.1038/s44318-025-00374-8 (PMC11914300; doi:10.1038/s44318-025-00374-8)
Supplement: Supplementary file 9 — Source data Fig. 5 [file 44318_2025_374_MOESM9_ESM.zip › EMBOJ-2024-118552_Source data_Fig. 5/5B/5B.pdf]

DMSO

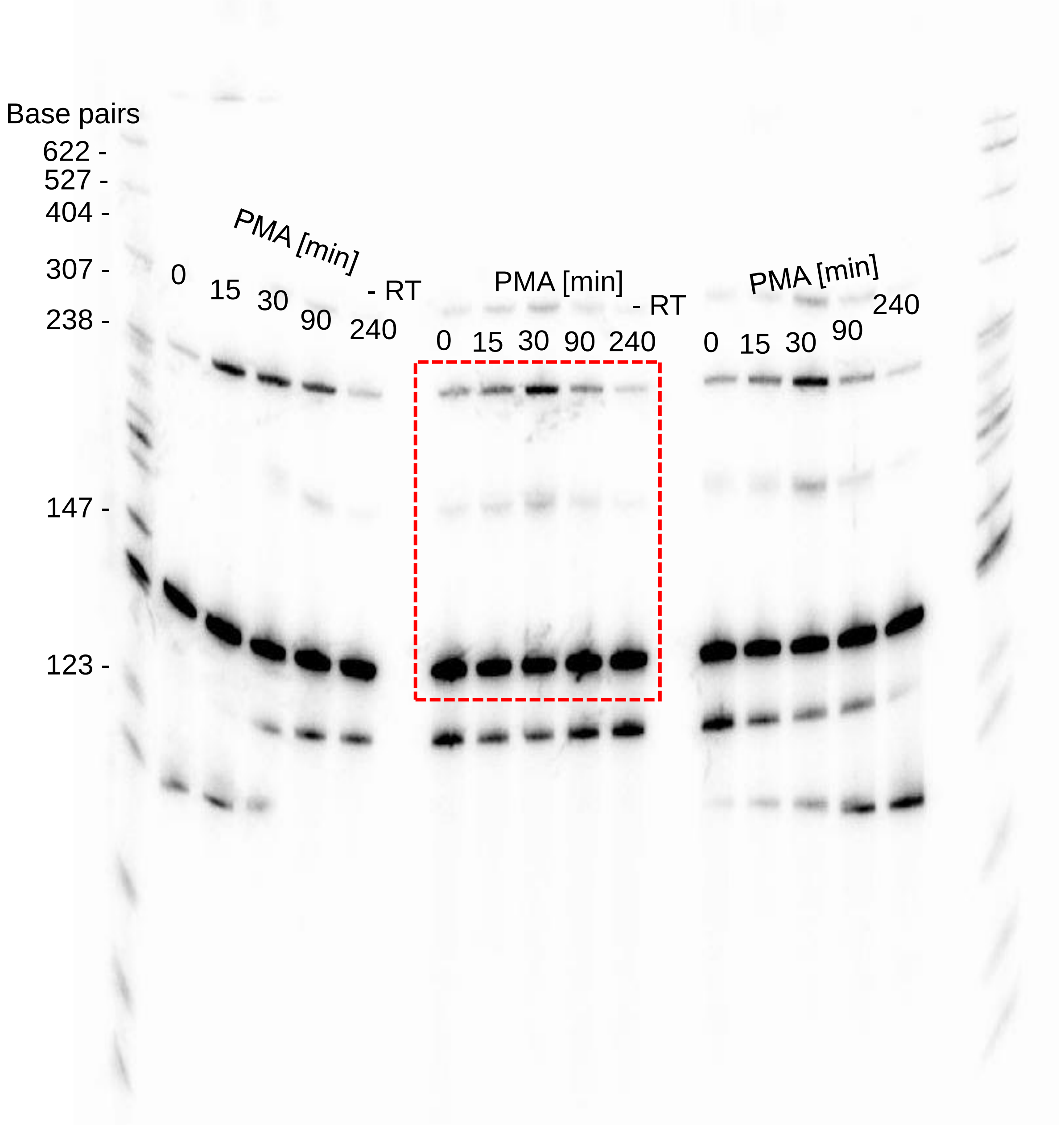

Base pairs
622 -
527 -
404 -
307 -
238 -
147 -
123 -
PMA [min]
0
15
30
90
240
- RT
PMA [min]
0
15
30
90
240
- RT
PMA [min]
0
15
30
90
240

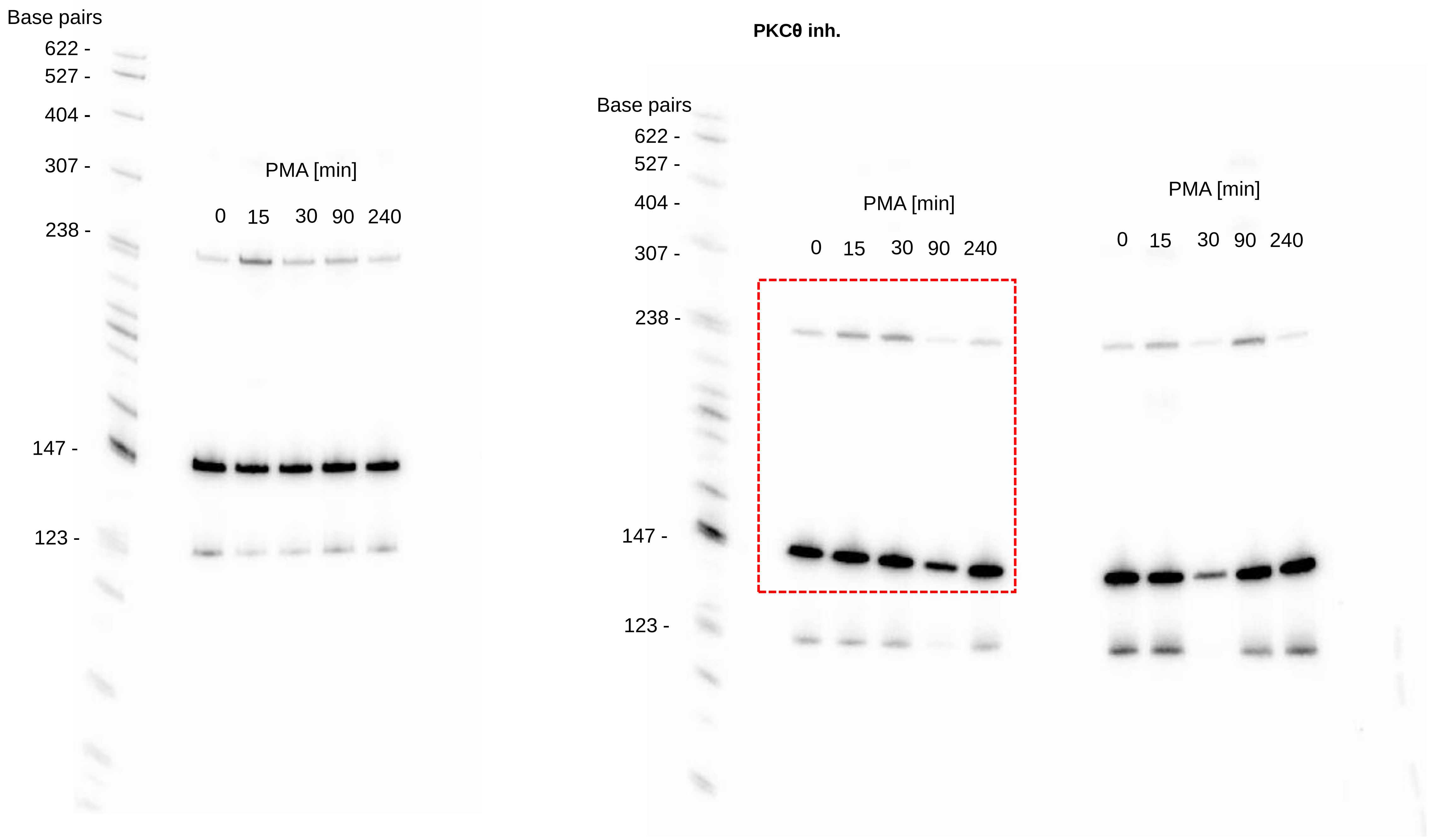
Base pairs
622 -
527 -
404 -
307 -
238 -
147 -
123 -
PMA [min]
0 15 30 90 240
PKCθ inh.
Base pairs
622 -
527 -
404 -
307 -
238 -
147 -
123 -
PMA [min]
0 15 30 90 240
PMA [min]
0 15 30 90 240
