# Supplementary material for: Immediate early splicing controls translation in activated T-cells and is mediated by hnRNPC2 phosphorylation
Source: EMBO J. 2025 Feb 13;44(6):1692–723. doi: 10.1038/s44318-025-00374-8 (PMC11914300; doi:10.1038/s44318-025-00374-8)
Supplement: Supplementary file 10 — Source data Fig. 6 [file 44318_2025_374_MOESM10_ESM.zip › EMBOJ-2024-118552_Source data_Fig. 6/6B/6B.pdf]

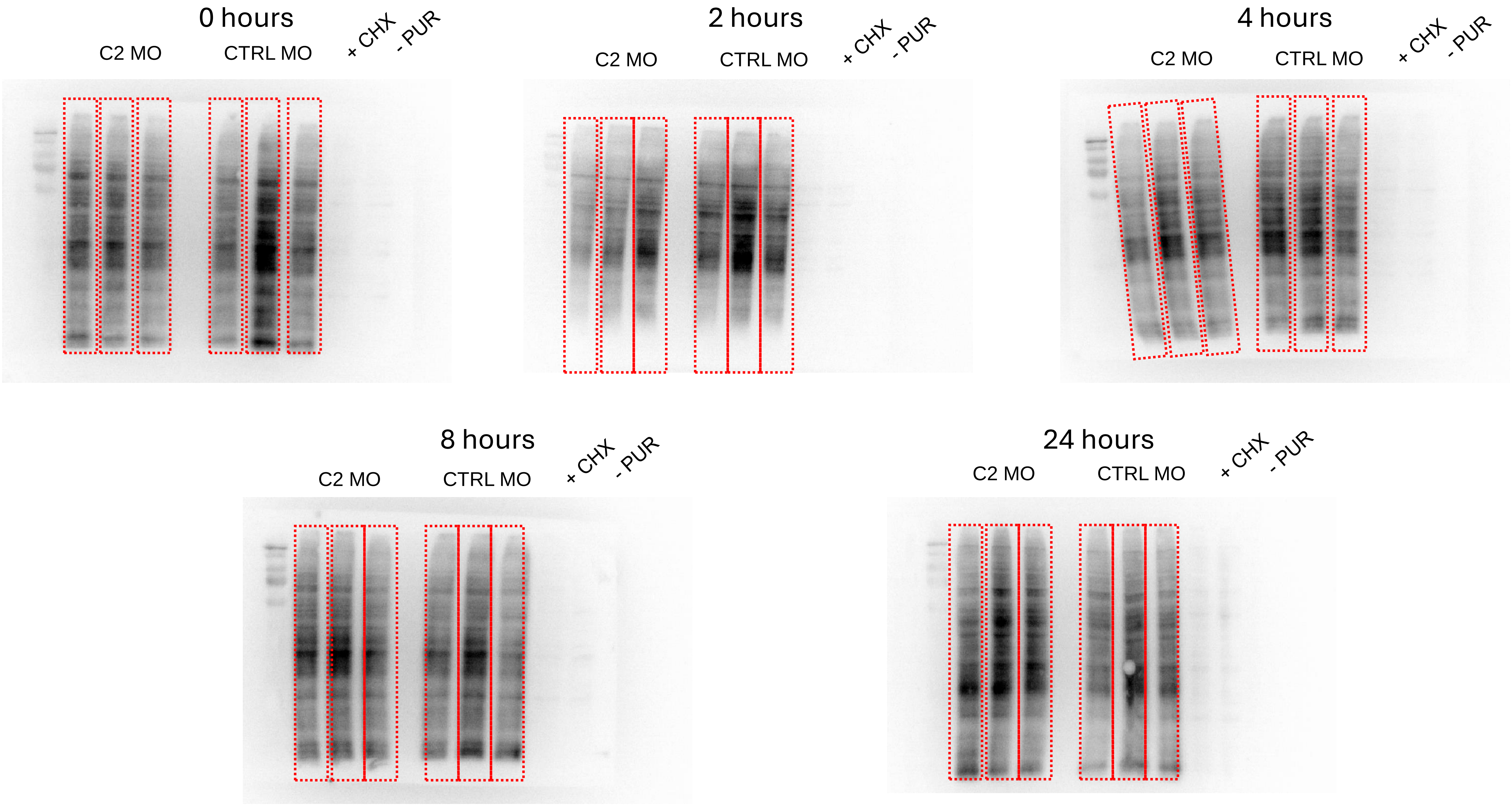
0 hours
C2 MO
CTRL MO
+ CHX
- PUR
2 hours
C2 MO
CTRL MO
+ CHX
- PUR
4 hours
C2 MO
CTRL MO
+ CHX
- PUR
8 hours
C2 MO
CTRL MO
+ CHX
- PUR
24 hours
C2 MO
CTRL MO
+ CHX
- PUR
