# Supplementary material for: Immediate early splicing controls translation in activated T-cells and is mediated by hnRNPC2 phosphorylation
Source: EMBO J. 2025 Feb 13;44(6):1692–723. doi: 10.1038/s44318-025-00374-8 (PMC11914300; doi:10.1038/s44318-025-00374-8)
Supplement: Supplementary file 11 — Expanded View Figures [file 44318_2025_374_MOESM11_ESM.pdf]

Expanded View Figures

Figure EV1. IES during T cell activation is independent of de novo protein synthesis.

(**A**) Jurkat cells were PMA-stimulated for the indicated times. Cells were harvested, total protein was extracted, and western blot was performed. A representative graph shows the activation of ERK1/2 upon PMA stimulation. (**B**) Jurkat cells were stimulated as in A. Cells were harvested and total RNA was extracted. Gene expression was analyzed by RT-qPCR (number of biological replicates: $n = 3$, mean ± SD). (**C**) rMATS analysis identifying significant changes (see methods) in alternative splicing of the types skipped exon (SE), retained introns (RI) alternative 5′ splice site (A5SS) or 3′ splice site (A3SS). Pairwise comparisons for 0 vs 30, 30 vs 150 and 0 vs 150 min are shown (from left to right). Increased RI events were observed when non-stimulated cells (0 min) were compared with PMA-stimulated cells for 30 min. (**D**) Box-whisker plots comparing intron length (left), MaxEntScan 5′ss (middle) and 3′ss (right) for introns retained after 30 min (IR_30) and introns more efficiently spliced (spliced_30) with all introns quantified by rMATS (all introns). Statistical significance was analyzed by Student's unpaired *t* tests and is indicated by asterisks (ns: non-significant, ***$P < 0.001$). Line represents median, box covers the interquartile interval and whiskers min to max. (**E**) Frequency distribution of IR changes. The maximal IR change (0 vs 30 or 30 vs 150 min) was calculated for all 173 significant introns. The histogram summarizes the frequency of IR changes in bin's differing by 5% (the number on the x-axis represents the center of each Bin). (**F**) Protein coding potential of IR isoforms. Our analysis shows that 169 of the 173 IR events occur in protein-coding genes, with 140 of these situated within the open reading frame (ORF). Of the 140 introns within the ORF, 136 introduce stop codons, either through frameshifts (93 cases) or by directly introducing a stop codon (43 cases). In 10 of these cases, the stop codon occurs in the last intron of the ORF, potentially producing an alternative C-terminus. In the remaining 126 cases, the introns would encode premature termination codons (PTCs). This leaves 4 cases that maintain in-frame sequences, including the translation initiation factor eIF5A. The other three genes are IRF3 (Interferon Regulatory Factor 3), MFSD10 (Major Facilitator Superfamily Domain-containing Protein 10), and TNFRSF1A (Tumor Necrosis Factor Receptor Superfamily Member 1A). Notably, IRF3, a key regulator of interferon alpha/beta transcription and other interferon-induced genes and has an alternative intron close to its DNA-binding domain, which might affect its transcriptional activity. TNFRSF1A, with an IR isoform, could potentially alter its TNF-alpha binding domain.

© The Author(s)

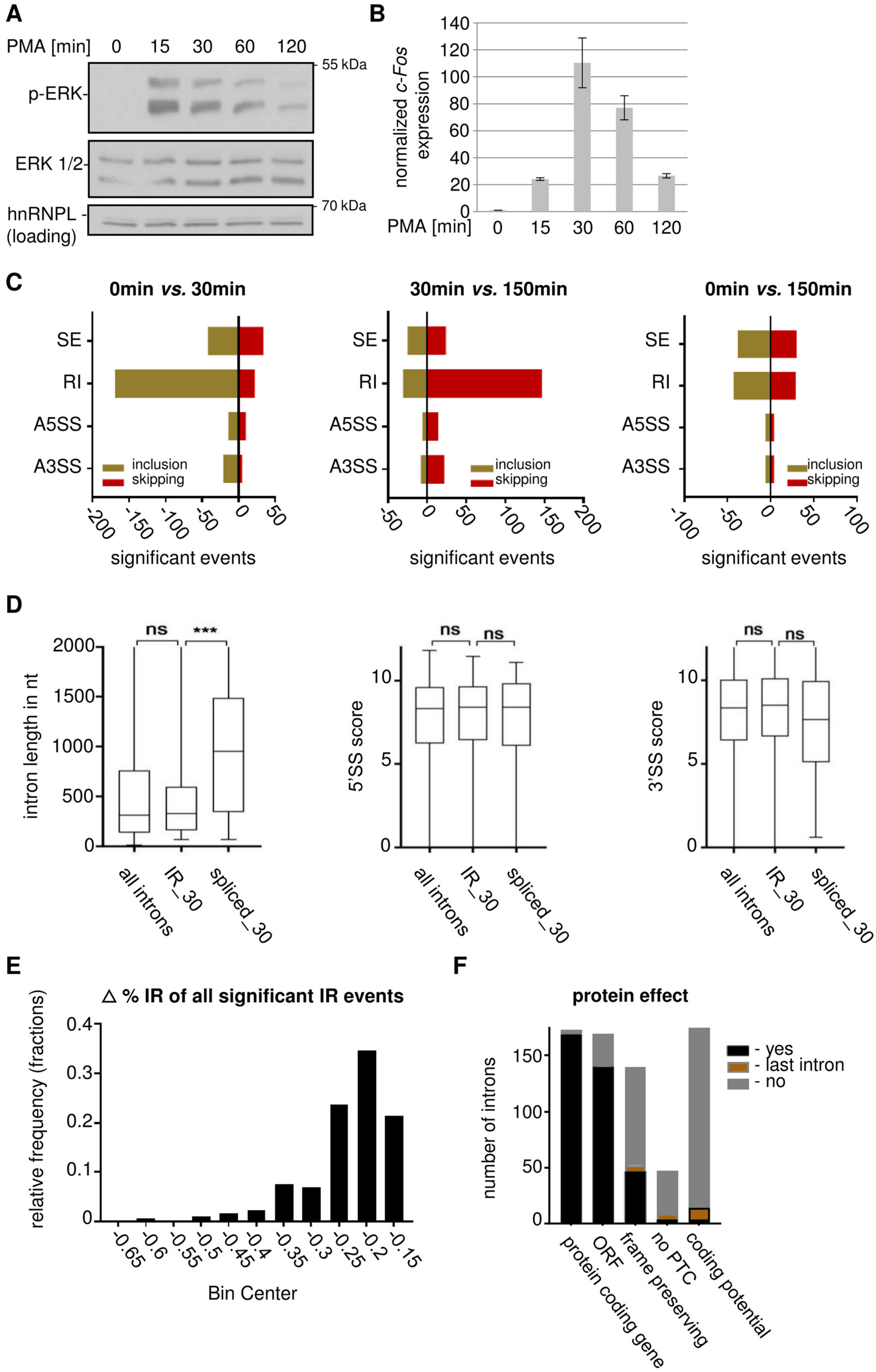
A
PMA [min] 0 15 30 60 120
p-ERK
ERK 1/2
hnRNPL (loading)
55 kDa
70 kDa
B
normalized c-Fos expression
PMA [min] 0 15 30 60 120
C
0min vs. 30min
30min vs. 150min
0min vs. 150min
SE
RI
A5SS
A3SS
inclusion
skipping
significant events
D
intron length in nt
5'SS score
3'SS score
ns
***
all introns
IR_30
spliced_30
E
Δ % IR of all significant IR events
relative frequency (fractions)
Bin Center
F
protein effect
number of introns
- yes
- last intron
- no
protein coding gene
ORF
frame preserving
no PTC
coding potential

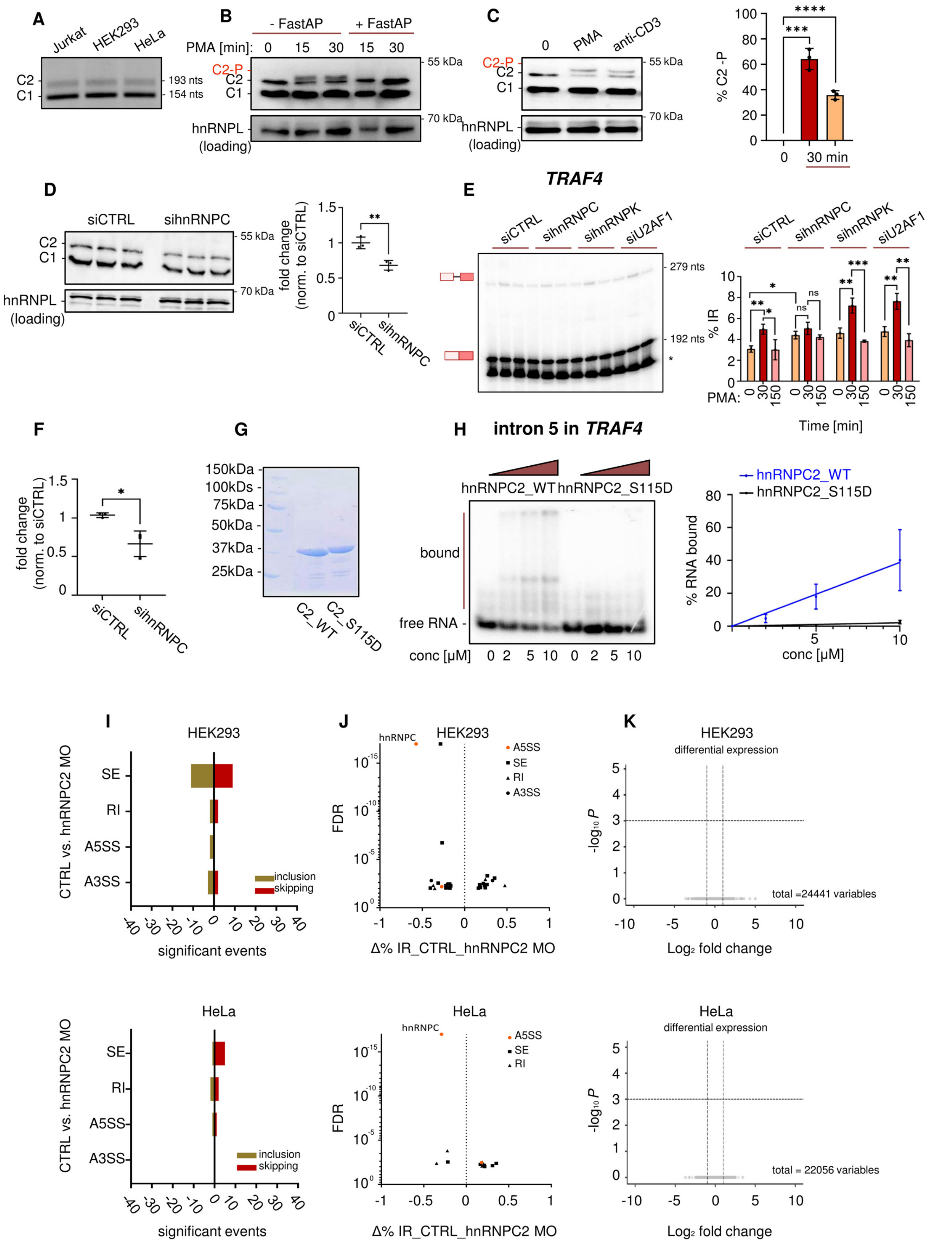
A
Jurkat
HEK293
HeLa
C2
C1
193 nts
154 nts
B
- FastAP
+ FastAP
PMA [min]: 0 15 30 15 30
C2-P
C2
C1
55 kDa
70 kDa
hnRNPL (loading)
C
0 PMA anti-CD3
% C2 -P
0 30 min
D
siCTRL sihnRNPC
fold change (norm. to siCTRL)
E
TRAF4
siCTRL sihnRNPC sihnRNPK siU2AF1
279 nts
192 nts
% IR
PMA: 0 30 150
Time [min]
F
G
150kDa 100kDs 75kDa 50kDa 37kDa 25kDa
C2_WT C2_S115D
H
intron 5 in TRAF4
hnRNPC2_WT hnRNPC2_S115D
bound
free RNA
conc [µM] 0 2 5 10 0 2 5 10
% RNA bound
conc [µM]
I
HEK293
CTRL vs. hnRNPC2 MO
SE RI A5SS A3SS
inclusion
skipping
significant events
J
HEK293
hnRNPC
A5SS SE RI A3SS
FDR
Δ% IR_CTRL_hnRNPC2 MO
K
HEK293
differential expression
-log₁₀ P
total =24441 variables
Log₂ fold change
HeLa
CTRL vs. hnRNPC2 MO
SE RI A5SS A3SS
inclusion
skipping
significant events
HeLa
hnRNPC
A5SS SE RI
FDR
Δ% IR_CTRL_hnRNPC2 MO
HeLa
differential expression
-log₁₀ P
total = 22056 variables
Log₂ fold change

 © The Author(s)

Figure EV2. IES is regulated by hnRNPC2.

(**A**) hnRNPC1/C2 isoform expression was detected in Jurkat cells, HEK293, and HeLa cells by standard splicing sensitive RT-PCR. (**B**) Jurkat cells were stimulated by PMA for the indicated times. FastAP was added to the indicated protein lysate for 1 h at 37 °C and lysates were analyzed by western blot. hnRNPL serves as loading control (number of biological replicates: $n = 3$). (**C**) Jurkat T cells were stimulated for 30 min by PMA and 5 µg anti-CD3 Ab. Left: Western blot presents increased phosphorylation of hnRNPC2 that was detected in PMA and anti-CD3 stimulated cells. hnRNPL serves as a loading control. Right: corresponding quantification (data are presented as % hnRNPC2 phosphorylation of total hnRNPC2, mean ± SD, number of biological replicates: $n = 3$, 0 vs. 30 min (PMA: dark red), $P = 0.0002$ vs. 30 min (anti-CD3: bright yellow), $P < 0.0001$, Student's unpaired t test). (**D**) Jurkat cells were transfected with siRNA against hnRNPC or control. Total protein was extracted. The efficiency of hnRNPC knockdown was analyzed by western blot (left, the gel shows triplicate samples) and quantified (right, data are presented as fold change normalized to siCTRL, mean ± SD, number of biological replicates: $n = 3$, $P = 0.0069$, Student's unpaired t test). hnRNPL serves as loading control. (**E**) IES in *TRAF4* depends on hnRNPC. Jurkat cells were transfected with siRNA against hnRNPC, hnRNPK or U2AF1 and, 48 h later, stimulated with PMA. Left: *TRAF4* IR was analyzed by radioactive, splicing-sensitive RT-PCR and quantified (right, data are presented as % IR, mean ± SD, number of biological replicates: $n = 3$, 0 vs. 30 min (siCTRL), $P = 0.0078$, siCTRL vs. sihnRNPC (0 min), $P = 0{,}007$, 30 vs. 150 min (siCTRL), $P = 0.0074$, 0 vs. 30 min (sihnRNPC), $P = 0.558$, 30 vs. 150 min (sihnRNPC), $P = 0.654$, 0 vs. 30 min (sihnRNPK), $P = 0.008$, 30 vs. 150 min (sihnRNPK), $P = 0.0003$, 0 vs. 30 min (U2AF1), $P = 0.0068$, 30 vs. 150 min (U2AF1), $P = 0.0059$, Student's unpaired t test). (**F**) HEK293 cells were transfected with siRNA against hnRNPC and control. After 48 h, cells were harvested, and RNA was extracted. The efficiency of hnRNPC knockdown was determined by RT-qPCR. mRNA expression is relative to hHPRT (data are presented as fold change normalized to siCTRL, mean ± SD, number of biological replicates: $n = 3$, $P = 0.0175$, Student's unpaired t test). (**G**) Coomassie-stained SDS-gel showing the amount of purified hnRNPC2 proteins used for EMSAs. (**H**) Increasing amounts of either hnRNPC_WT or S115D (0 µM, 2 µM, 5 µM, 10 µM) were complexed with 10 pmol of radioactively labeled RNA corresponding to a part of the *TRAF4* intron 5 that includes the polypyrimidine tract and the 3′ splice site. Left: representative native gel shows a reduction of RNA binding in the S115D phosphomimetic-mutant. Data are representative of at least three independent experiments. Right: corresponding quantification of experiment performed in triplicates. (**I**) HEK293 cells (top) and HeLa cells (bottom) were transfected with an hnRNPC2-inducing morpholino (hnRNPC2 MO), or control MO (CTRL MO). After 48 hours, cells were harvested, total RNA was extracted and RNA-seq was performed. Plots represent rMATs splicing analysis in HEK293 and HeLa cells after hnRNPC2 MO treatment. Only significant events are shown. Note that only very few splicing events are affected. (**J**) Volcano plots from HEK293 (top) and HeLa (bottom) cells show that hnRNPC alternative splicing is the most (and almost only) highly significant splicing event detected after MO treatment. This shows high efficiency of the MO, and basically no effect of shifting the hnRNPC1:C2 ratio on other splicing events. (**K**) Volcano plots as in J showing no difference in gene expression between hnRNPC MO samples and CTRL.

© The Author(s)

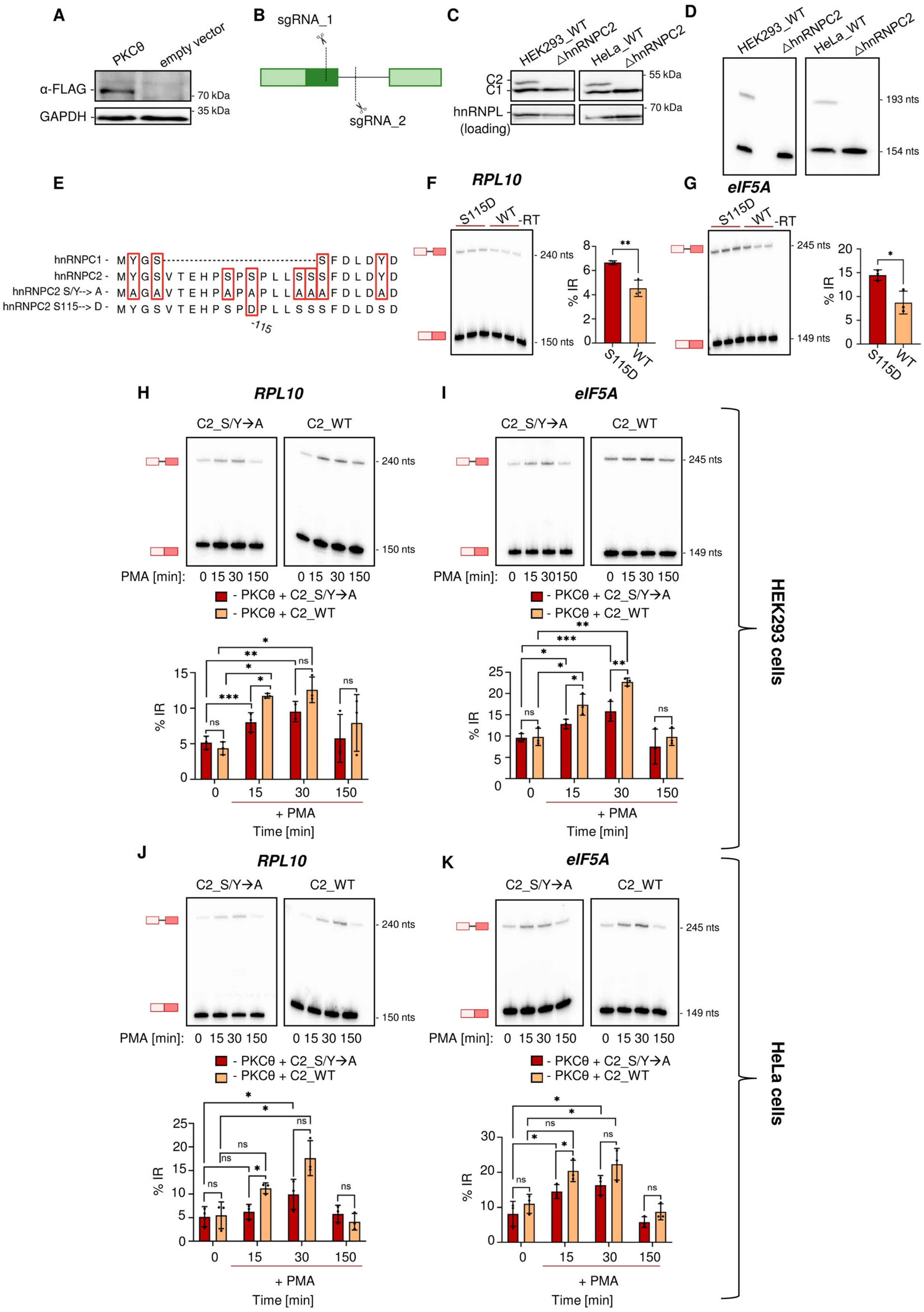
A
PKCθ
empty vector
α-FLAG
GAPDH
70 kDa
35 kDa
B
sgRNA_1
sgRNA_2
C
HEK293_WT
△hnRNPC2
HeLa_WT
△hnRNPC2
C2
C1
hnRNPL (loading)
55 kDa
70 kDa
D
HEK293_WT
△hnRNPC2
HeLa_WT
△hnRNPC2
193 nts
154 nts
E
hnRNPC1 - M Y G S S F D L D Y D
hnRNPC2 - M Y G S V T E H P S P S P L L S S S F D L D Y D
hnRNPC2 S/Y--> A - M A G A V T E H P A P A P L L A A A F D L D A D
hnRNPC2 S115--> D - M Y G S V T E H P S P D P L L S S S F D L D S D
115
F
RPL10
S115D
WT
-RT
240 nts
150 nts
% IR
G
eIF5A
245 nts
149 nts
H
RPL10
I
eIF5A
C2_S/Y→A
C2_WT
PMA [min]: 0 15 30 150
- PKCθ + C2_S/Y→A
- PKCθ + C2_WT
+ PMA
Time [min]
HEK293 cells
J
RPL10
K
eIF5A
HeLa cells

Figure EV3. PKCθ and hnRNPC2 are required for IES.

(**A**) HEK293 cells were transfected with an overexpression vector for PKCθ and empty FLAG vector. After 48 h, total protein was extracted. Western blot confirms PKCθ (anti-FLAG) overexpression with GAPDH as loading control. (**B**) Schematic view of the deletion of the hnRNPC2-generating 5′ splice site of exon 4 using CRISPR/Cas9. Scissors show the position of the sgRNAs. Created with BioRender.com. (**C**) HEK293_WT and CRISPR/Cas9-edited HEK293 with a deletion of hnRNPC2 (left) were harvested and total protein was extracted. Western Blot shows the deletion of hnRNPC2. hnRNPL acts as loading control. The same approach was used in HeLa cells shown on the right. (**D**) Clones of HEK293_WT, HeLa_WT and CRISPR/Cas9-edited HEK293 and HeLa cells with a deletion of hnRNPC2 were harvested and RNA was extracted. Radioactive, splicing-sensitive RT-PCR confirms the absence of hnRNPC2. (**E**) Alignment of hnRNPC1, hnRNPC2, a nonphosphorylatable version of hnRNPC2 and phosphomimetic version of hnRNPC2 (S115→D). All potential phosphorylation sites in hnRNPC2, including Ser115 and some surrounding residues in hnRNPC1 were mutated. Mutated residues are highlighted in red frames. (**F**, **G**) hnRNPC2 WT and its phosphomimetic version S115D were overexpressed in HEK293 cells. After 48 h, cells were harvested, and chromatin-associated RNA was extracted. *RPL10* (**F**) and *eIF5A* (**G**) IR were analyzed by radioactive, splicing-sensitive RT-PCR (left) and quantified (right, data are presented as % IR, mean ± SD, number of biological replicates: $n = 3$, *RPL10* (**F**): $P = 0.006$, *eIF5A* (G): $P = 0.0192$, Student's unpaired *t* test). (**H**, **I**) PKCθ-induced IES depends on hnRNPC2 phosphorylation. HEK293 cells were co-transfected with PKCθ and either hnRNPC2_WT or the nonphosphorylatable version. Cells were treated and analyzed as in Fig. EV3F, G (*RPL10* (**H**) and *eIF5A* (**I**), data are presented as % IR, mean ± SD, number of biological replicates: $n = 3$, *RPL10* (**H**): PKCθ + C2_S/Y→A vs. PKCθ + C2_WT (0 min), $p = 0.3541$, PKCθ + C2_S/Y→A vs. PKCθ + C2_WT (15 min), $P = 0.0102$, PKCθ + C2_S/Y→A vs. PKCθ + C2_WT (30 min), $P = 0.141$, PKCθ + C2_S/Y→A vs. PKCθ + C2_WT (150 min), $P = 0.8609$, 0 vs. 15 min (PKCθ + C2_S/Y), $P = 0.0397$, 0 vs. 30 min (PKCθ + C2_S/Y), $P = 0.023$, 0 vs. 15 min (PKCθ + C2_WT), $P = 0.0002$, 0 vs. 30 min (PKCθ + C2_WT), $P = 0.0021$, *eIF5A* (**I**): PKCθ + C2_S/Y→A vs. PKCθ + C2_WT (0 min), $P = 0.881$, PKCθ + C2_S/Y→A vs. PKCθ + C2_WT (15 min), $P = 0.0309$, PKCθ + C2_S/Y→A vs. PKCθ + C2_WT (30 min), $P = 0.0016$, PKCθ + C2_S/Y→A vs. PKCθ + C2_WT (150 min), $P = 0.4382$, 0 vs. 15 min (PKCθ + C2_S/Y), $P = 0.0185$, 0 vs. 30 min (PKCθ + C2_S/Y), $P = 0.0079$, 0 vs. 15 min (PKCθ + C2_WT), $P = 0.0208$, 0 vs. 30 min (PKCθ + C2_WT), $P = 0.0006$, Student's unpaired *t* test). (**J**, **K**) Experiments as in (**H**, **I**) using HeLa cells. Data are presented as % IR, mean ± SD, number of biological replicates: $n = 3$, *RPL10* (**J**): PKCθ + C2_S/Y→A vs. PKCθ + C2_WT (0 min), $P = 0.8923$, PKCθ + C2_S/Y→A vs. PKCθ + C2_WT (15 min), $P = 0.1341$, PKCθ + C2_S/Y→A vs. PKCθ + C2_WT (30 min), $P = 0.1245$, PKCθ + C2_S/Y→A vs. PKCθ + C2_WT (150 min), $P = 0.3176$, 0 vs. 15 min (PKCθ + C2_S/Y), $P = 0.1341$, 0 vs. 30 min (PKCθ + C2_S/Y), $P = 0.0353$, 0 vs. 15 min (PKCθ + C2_WT), $P = 0.3416$, 0 vs. 30 min (PKCθ + C2_WT), $P = 0.0107$, *eIF5A* (**K**): PKCθ + C2_S/Y→A vs. PKCθ + C2_WT (0 min), $P = 0.327$, PKCθ + C2_S/Y→A vs. PKCθ + C2_WT (15 min), $P = 0.0488$, PKCθ + C2_S/Y→A vs. PKCθ + C2_WT (30 min), $P = 0.1224$, PKCθ + C2_S/Y→A vs. PKCθ + C2_WT (150 min), $P = 0.1382$, 0 vs. 15 min (PKCθ + C2_S/Y), $P = 0.0526$, 0 vs. 30 min (PKCθ + C2_S/Y), $P = 0.035$, 0 vs. 15 min (PKCθ + C2_WT), $P = 0.0163$, 0 vs. 30 min (PKCθ + C2_WT), $P = 0.0206$, Student's unpaired *t* test).

© The Author(s)

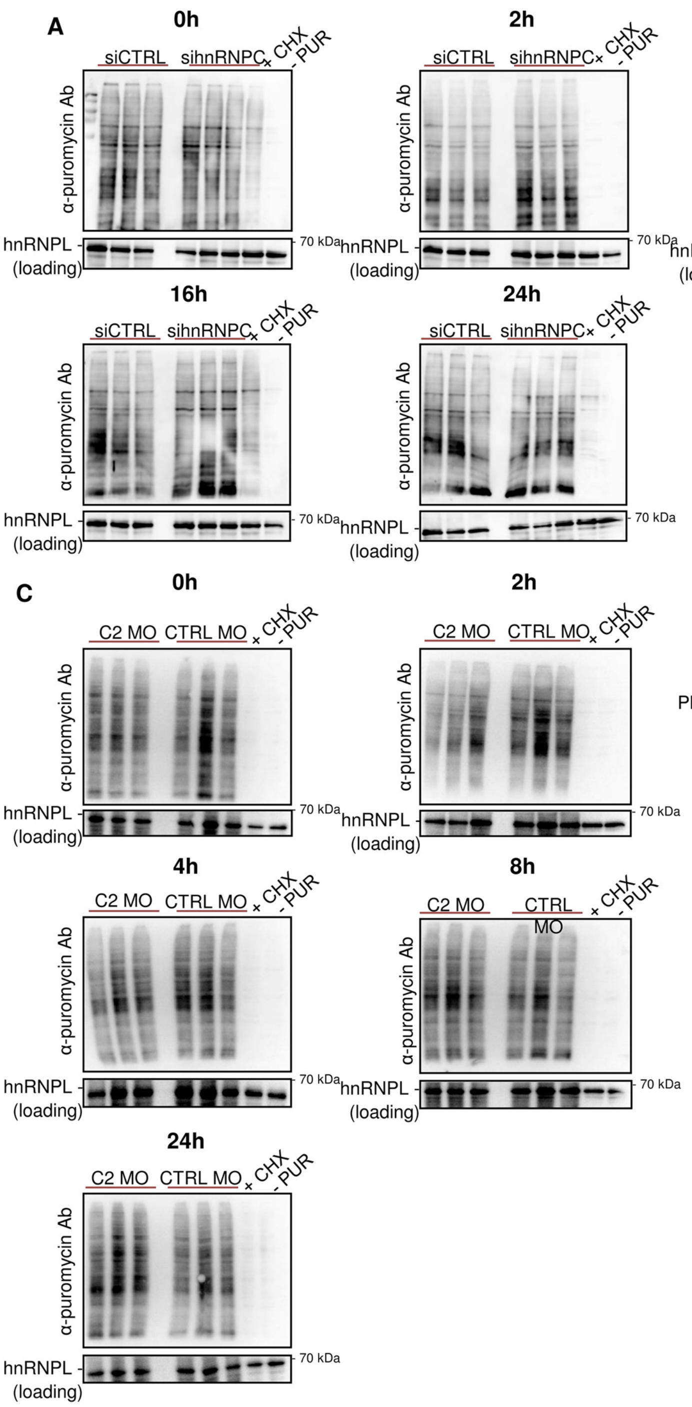

Figure EV4. IES reduces global translation.

(**A**) Triplicate samples for the analysis in Fig. 6A. (**B**) De novo translation in Jurkat cells after PMA activation in control and hnRNPC knockdown conditions at indicated time points analyzed using ^{35}S-Met incorporation. Upper gels show commassie-loading control, bottom gels show autoradiographs (representative gels). Bottom: corresponding quantification (data are presented as fold change normalized to 0 h, mean ± SD, number of biological replicates: $n = 3$, 0 vs. 1 h (siCTRL), $P = 0.0005$, 0 vs. 2 h (siCTRL), $P = 0.0004$, 0 vs. 4 h (siCTRL), $P = 0.0035$, 0 vs. 16 h (siCTRL), $P < 0.0001$, sihnRNPC vs. siCTRL (1 h), $P = 0.002$, sihnRNPC vs. siCTRL (2 h), $P = 0.0043$, sihnRNPC vs. siCTRL (4 h), $P = 0.023$ (Student's unpaired t test). (**C**) Triplicate samples for the analysis in Fig. 6B.

 © The Author(s)

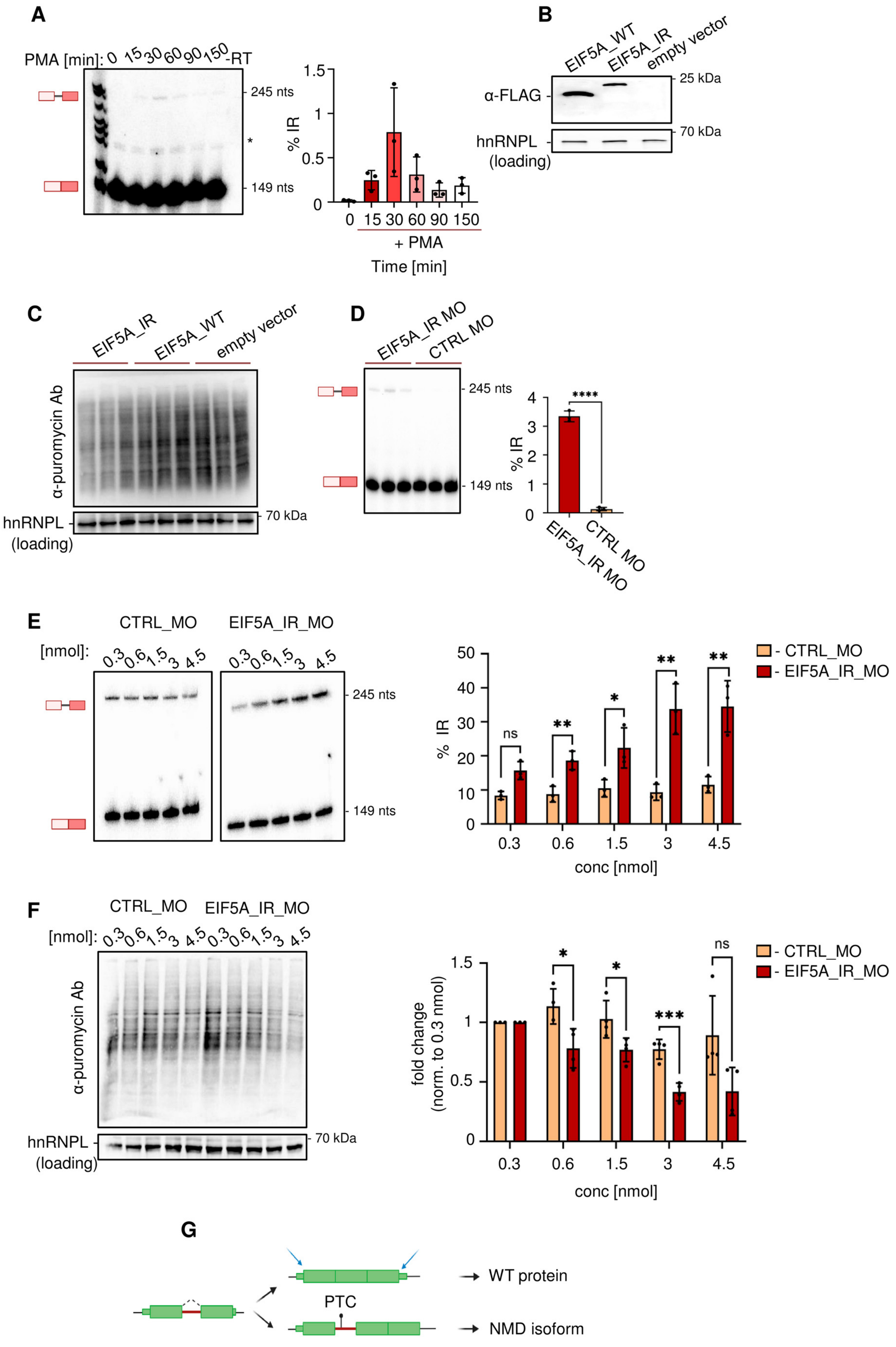
A
PMA [min]: 0 15 30 60 90 150 -RT
245 nts
*
149 nts
% IR
1.5
1
0.5
0
0 15 30 60 90 150
+ PMA
Time [min]
B
EIF5A_WT
EIF5A_IR
empty vector
α-FLAG
25 kDa
hnRNPL
(loading)
70 kDa
C
EIF5A_IR
EIF5A_WT
empty vector
α-puromycin Ab
hnRNPL
(loading)
70 kDa
D
EIF5A_IR MO
CTRL MO
245 nts
149 nts
% IR
****
4
3
2
1
0
EIF5A_IR MO
CTRL MO
E
CTRL_MO
EIF5A_IR_MO
[nmol]: 0.3 0.6 1.5 3 4.5 0.3 0.6 1.5 3 4.5
245 nts
149 nts
% IR
50
40
30
20
10
0
ns
**
*
**
**
0.3
0.6
1.5
3
4.5
conc [nmol]
- CTRL_MO
- EIF5A_IR_MO
F
CTRL_MO
EIF5A_IR_MO
[nmol]: 0.3 0.6 1.5 3 4.5 0.3 0.6 1.5 3 4.5
α-puromycin Ab
hnRNPL
(loading)
70 kDa
fold change
(norm. to 0.3 nmol)
1.5
1
0.5
0
*
*
***
ns
0.3
0.6
1.5
3
4.5
conc [nmol]
- CTRL_MO
- EIF5A_IR_MO
G
WT protein
PTC
NMD isoform

Figure EV5. The EIF5A IES isoform is sufficient to globally reduce translation.

(**A**) The EIF5A IES product is exported to cytoplasm. Jurkat cells were PMA stimulated for the indicated times and cytoplasmic RNA was extracted. Left: representative gel from radioactive, splicing-sensitive PCR showing IR events in cytoplasm after T cell activation. * - degradation product, -RT; without reverse transcriptase. Right: corresponding quantification (data are presented as % IR, number of biological replicates: $n = 3$). (**B**) HEK293 cells were transfected with eIF5A expression constructs (WT and IR). After 48 h, cells were harvested, and total protein was extracted. Western blot confirms overexpression with hnRNPL as loading control. (**C**) Triplicate samples for experiments in Fig. 6D. (**D**) Jurkat cells were electroporated with either eIF5A_IR MO to induce intron retention or control MO for 48 h. Cells were harvested and cytoplasmic RNA was extracted. Left: gel from radioactive, splicing-sensitive PCR showing IR events in cytoplasm after T cell activation. Gel shows triplicate samples. Right: corresponding quantification (data are presented as % IR, mean ± SD, number of biological replicates: $n = 3$, $P < 0.0001$, Student's unpaired t test). (**E**) Titration experiment with increasing concentrations of EIF5A_IR_MO. Jurkat cells were electroporated with increasing concentrations of EIF5A_IR_MO (from 0.3 nmol to 4.5 nmol). Left: representative gel from radioactive, splicing-sensitive PCR; * degradation product. Right: corresponding quantification (data are presented as % IR, mean ± SD, number of biological replicates: $n = 3$, CTRL_MO vs. EIF5A_IR_MO (0.3 nmol), $P = 0.0511$, CTRL_MO vs. EIF5A_IR_MO (0.6 nmol), $P = 0.0089$, CTRL_MO vs. EIF5A_IR_MO (1.5 nmol), $P = 0.0333$, CTRL_MO vs. EIF5A_IR_MO (3 nmol), $P = 0.0056$, CTRL_MO vs. EIF5A_IR_MO (4.5 nmol), $P = 0.0073$ (Student's unpaired t test). (**F**) Titration experiment with increasing concentrations of EIF5A_IR_MO. Jurkat cells were electroporated with increasing concentrations of EIF5_IR_MO as in (**E**). After 48 h, cells were treated with 10 µg/ml of puromycin 10 min before harvesting. Total protein was extracted. Left: representative blot of the WB-SUnSET experiment. hnRNPL serves as loading control. Right: corresponding quantification (data are presented as fold change normalized to 0.3 nmol, mean ± SD, number of biological replicates: $n = 3$, CTRL_MO vs. EIF5A_IR_MO (0.6 nmol), $P = 0.0187$, CTRL_MO vs. EIF5A_IR_MO (1.5 nmol), $P = 0.0318$, CTRL_MO vs. EIF5A_IR_MO (3 nmol), $P = 0.0007$, CTRL_MO vs. EIF5A_IR_MO (4.5 nmol), $P = 0.0511$ (Student's unpaired t test). (**G**) Schematic representation of the pre-mRNAs of *RPL10*. Due to the presence of a PTC, IR likely induces NMD. Green boxes represent exons, dashed lines show introns, blue arrows show start and stop codon. Created with BioRender.com.

 © The Author(s)
